# Autoimmune diseases and adverse pregnancy outcomes: an umbrella review

**DOI:** 10.1186/s12916-024-03309-y

**Published:** 2024-03-05

**Authors:** Megha Singh, Steven Wambua, Siang Ing Lee, Kelvin Okoth, Zhaonan Wang, Fathima Fazla Ahamed Fayaz, Kelly-Ann Eastwood, Catherine Nelson-Piercy, John A. Reynolds, Krishnarajah Nirantharakumar, Francesca Crowe, on behalf of MuM-PreDiCT

**Affiliations:** 1 Institute of Applied Health Research, University of Birmingham, Birmingham, UK; 2 Centre for Public Health, University of Belfast, Belfast, Queen BT7 1NN UK; 3 Michael’s Hospital, University Hospitals Bristol NHS Foundation Trust, Bristol, BS2 8EG UK; 4 Guy’s and St, Thomas’ NHS Foundation Trust, London, SE1 7EH UK; 5 Institute of Inflammation and Ageing, University of Birmingham, Birmingham, UK

**Keywords:** Autoimmune diseases, Pregnancy complications, Pregnancy

## Abstract

**Background:**

There is a high prevalence of autoimmune conditions in women specially in the reproductive years; thus, the association with adverse pregnancy outcomes has been widely studied. However, few autoimmune conditions/adverse outcomes have been studied more than others, and this umbrella review aims to consolidate existing knowledge in this area with the aim to provide new knowledge and also identify gaps in this research area.

**Methods:**

Medline, Embase, and Cochrane databases were searched from inception to December 2023. Screening, data extraction, and quality appraisal (AMSTAR 2) were done by two independent reviewers. Data were synthesised narratively and quantitatively. Relative risks (RR)/odds ratio (OR) with 95% confidence intervals were reported.

**Results:**

Thirty-two reviews were included consisting of 709 primary studies. The review reported the association between 12 autoimmune conditions and 16 adverse pregnancy outcomes. Higher risk of miscarriage is reported in women with Sjögren’s syndrome RR 8.85 (95% CI 3.10–25.26) and systemic lupus erythematosus (SLE) OR 4.90 (3.10–7.69). Pre-eclampsia was reported higher in women with type 1 diabetes mellitus (T1DM) OR 4.19 (3.08–5.71) and SLE OR 3.20 (2.54–4.20). Women reported higher risk of diabetes during pregnancy with inflammatory bowel disease (IBD) OR 2.96 (1.47–5.98). There was an increased risk of intrauterine growth restriction in women with systemic sclerosis OR 3.20 (2.21–4.53) and coeliac disease OR 1.71 (1.36–2.14). Preterm birth was associated with T1DM OR 4.36 (3.72–5.12) and SLE OR 2.79 (2.07–3.77). Low birth weight babies were reported in women with women with SLE or systemic sclerosis OR 5.95 (4.54–7.80) and OR 3.80 (2.16–6.56), respectively. There was a higher risk of stillbirth in women with T1DM OR 3.97 (3.44–4.58), IBD OR 1.57 (1.03–2.38), and coeliac disease OR 1.57 (1.17–2.10). T1DM in women was associated with 32% lower odds of small for gestational age baby OR 0.68 (0.56–0.83).

**Conclusions:**

Pregnant women with autoimmune conditions are at a greater risk of developing adverse pregnancy outcomes. Further research is required to develop better preconception to postnatal care for women with autoimmune conditions.

**Supplementary Information:**

The online version contains supplementary material available at 10.1186/s12916-024-03309-y.

## Background

There are over 80 different types of autoimmune conditions with about 80% of those diagnosed with these conditions being women [1–3]. The prevalence of autoimmune conditions is higher in women by almost twofold and is often associated with the X chromosome [4–7]. For many individual autoimmune conditions, there is a higher female-to-male ratio, e.g. systemic lupus erythematosus (SLE) 7:1, Sjogren’s syndrome 9:1, and rheumatoid arthritis or systemic sclerosis 3:1 [8–10]. Women undergo various hormonal changes throughout their life: during puberty, pregnancy, and then in menopause [11]. These endocrine transitions in women may increase their susceptibility to autoimmune conditions, and many autoimmune conditions such as SLE, systemic sclerosis, rheumatoid arthritis, and psoriasis develop during the female reproductive age influenced by the T cell cytokine-mediated response and hormonal, immunological, and bodily changes [11, 12]. Earlier literature also reported autoimmune conditions as the seventh most frequent underlying cause of death among females in age groups below 75 years in the UK and USA [5, 13].

There has been an increasing trend of autoimmune conditions worldwide. Lerner et al. estimated the net % increase per year for the incidence and prevalence of autoimmune conditions is 19.1% and 12.5%, respectively, in the last 30 years [14, 15]. This increasing trend is due to many environmental factors like lifestyle changes, changes in diet, and exposure to certain infections and drugs [16–23]. With this increase in the prevalence and thereby an increased number of pregnancies presenting with autoimmune conditions, it is pertinent to have a clear idea of the adverse pregnancy outcomes associated with the specific autoimmune conditions and to identify whether these outcomes are unique to specific conditions or are shared across the spectrum of autoimmunity.

Symptoms of autoimmune conditions could improve, worsen, or remain unchanged when a woman becomes pregnant depending upon her specific autoimmune condition. For example, improvement in the symptoms of rheumatoid arthritis during pregnancy has been observed in some women whereas worsening of symptoms is a common feature in SLE [24, 25]. Autoimmune conditions may complicate pregnancy as antibodies that the mother produces can enter the foetus’s system, e.g. anti-Ro/SSA antibodies crossing the placenta and impacting development of the foetal heart [26]. These conditions have variable course and are episodic in nature, making it harder to determine the impact of pregnancy in triggering and/or progressing the condition and thereafter its impact on the pregnancy outcome [25]. Clinical management of pregnancies with autoimmune condition requires interdisciplinary care with clear preconception care protocol/recommendations and clear understanding of the risk of adverse pregnancy outcomes [27, 28].

Many systematic reviews have examined the role of autoimmune conditions on both maternal and foetal outcomes [29–31]. It has been well established that autoimmune conditions like SLE, and inflammatory bowel disease (IBD) may have adverse pregnancy outcomes like miscarriage and preterm birth [32–35]. Conditions like multiple sclerosis are known to have an adverse effect on pregnancy outcomes, but evidence is sparse to support this [29, 36]. Some autoimmune conditions have been studied more than others with inconsistent findings [37, 38]. This umbrella review aims to consolidate evidence from systematic reviews of the association of common autoimmune conditions with pregnancy outcomes in order to identify the strength and precision of these associations. Also, if foetal or maternal outcomes are shared between autoimmune disease, this might suggest a common pathological process which could be targeted across conditions, which will help identify the potential gaps in current research to help prioritise the future research in autoimmune conditions with limited evidence in this area [39].

## Methods

This umbrella review aims to summarise the evidence available in the form of systematic reviews studying the adverse pregnancy outcomes in women with autoimmune conditions. This umbrella review has been conducted in accordance with Joanna Briggs Institute (JBI) umbrella review methodology [40], and the PRIOR (Preferred Reporting Items for Overviews of Reviews) checklist was used to report the review [41]. The protocol has been registered to PROSPERO (registration number CRD4202233499). Deviations from the protocol are listed in Additional file 1: Table S2.

### Inclusion and exclusion criteria

Systematic reviews reporting the associations between autoimmune conditions and adverse pregnancy outcomes were included. No language restriction was applied. The population considered were pregnant women without any age restriction. We did not include reviews where all women who were pregnant were as a result of assisted reproductive treatment because this presents with its own set of risks. The autoimmune conditions that were selected were those that were more common in women of reproductive age and after consultation with experts in the subject [1, 42]. Furthermore, a scoping search was conducted before finalising the list of exposures and outcomes. Autoimmune conditions included Addison’s disease, alopecia areata, axial spondyloarthropathy (AxSpA), coeliac disease, IBD including Crohn’s disease and ulcerative colitis, multiple sclerosis (MS), myasthenia gravis, psoriatic diseases (including psoriasis and psoriatic arthritis), rheumatoid arthritis, Sjögren’s syndrome, SLE, systemic sclerosis, and thyroid autoimmunity (including Grave’s disease and Hashimoto's thyroiditis), and type 1 diabetes mellitus (T1DM) and vitiligo.

The outcomes included were adverse pregnancy outcomes which were considered after consultations with experts (obstetricians and epidemiologists) and after input from patient public involvement and engagement (PPIE) group members. The outcomes definitions were defined prior [43], and definitions were compared between the reviews. The outcomes are listed in Table 1.

**Table 1:** Outcomes—adverse pregnancy outcomes

**Maternal outcomes**
1. Miscarriage/recurrent miscarriage/spontaneous pregnancy loss
2. Hypertensive disorders of pregnancy (gestational hypertension pre-eclampsia- early or late onset, recurrent pre-eclampsia, HELLP (haemolysis, elevated liver enzymes, and low platelet) syndrome)
3. Placental disorders (placenta previa, placental abruption, placenta accreta, placenta percreta)
4. Hyperemesis gravidarum
5. Gestational diabetes mellitus (GDM)
6. Ectopic pregnancy
7. Molar pregnancy/choriocarcinoma
8. Obstetric cholestasis
9. Obstetric haemorrhage
10. Mode of birth: caesarean, instrumental
11. Perineal trauma—3rd and 4th degree tear
12. Postpartum depression
13. Puerperal psychosis
**Foetal/neonatal outcomes**
1. Intrauterine growth restriction (IUGR)
2. Small for gestational age (SGA)
3. Stillbirth
4. Preterm birth/recurrent preterm birth
5. Low birth weight
6. Neonatal death

Systematic reviews were included with or without a meta-analysis. The identified reviews were carefully examined to determine whether the review qualified as systematic review [44]. The reviews were excluded (1) if the review was not qualified as systematic review, e.g. scoping reviews, reviews, protocols, conference abstracts; (2) if they did not report the associations of the specified autoimmune conditions and adverse pregnancy outcome/s or (3) if studying the association of drugs for autoimmune conditions and the pregnancy outcome/s; (4) if comparing the effects of one autoimmune condition to another; and (5) duplicates.

### Search strategy

Medline, Embase, and Cochrane database were systematically searched from the inception to 15 December 2023. A robust search strategy was used, and the systematic review filter was used to limit the searches. The search was repeated periodically to identify the latest published reviews. The Medical Subject Headings and free text search for autoimmune conditions (exposure) and pregnancy outcomes were used. The detailed search strategy for Medline is presented in Additional file 1: Table S3. This search strategy was adapted for use in other databases.

### Study selection

Once the literature search was completed, a reference management software (EndnoteV.X9) was used to manage the studies. After removing duplicate studies, two independent reviewers (MS, SW) conducted the title and abstract screening, and ineligible studies were excluded. Full-text screening of eligible studies was conducted by two independent reviewers (MS, SW), and a third senior reviewer (FC, KN) was consulted to resolve any discrepancy. The list of excluded studies with reasons for excluding them is shown in Additional file 1: Table S4 [31, 45–103]. We found four non-English reviews. One was in Spanish, and three were in Mandarin. Fellow researchers with expertise in these languages were consulted. They translated the reviews and performed the data extraction and quality assessment for these reviews. The review in Spanish was excluded after the full text screening, but the three reviews in Mandarin were included.

### Data extraction

Two independent reviewers (MS, SW) extracted the data from the reviews, and in the case of discrepancy, a third reviewer (FC, KN) was consulted. Data was extracted under the following headings: aim of the review; database searched; search period; exposures; comparator; outcomes; study design(s); definition of exposure; definition of outcome; data synthesis method; quality assessment tool; quality of the included primary studies as assessed by review authors; various characteristics for example year of publication, geographical area, type of studies included, etc., of the included reviews; effect sizes; and conclusion of the review. The authors of the reviews were contacted where further information was required. The data extraction form used is shown in Additional file 1: Table S5.

### Quality assessment

Assessment of multiple systematic reviews version 2 (AMSTAR 2) checklist was completed by two reviewers independently (MS, SW) to assess the methodological quality of the included reviews [104]. In case of any disagreements, a third reviewer (FC, KN) was consulted to resolve these. The reviews were rated in four categories: high, moderate, low, and critically low. Out of the 16 items on the AMSTAR 2 checklist, seven were considered critical. These were as follows: registration of the protocol before starting the reviews, conduct of an adequate search of the literature, providing justification for the exclusion of individual studies, satisfactory assessment of risk of bias in the studies included in the reviews, use of appropriate statistical methods in performing a meta-analysis, accounting for risk of bias when interpreting the results. If any of these critical domains were not fulfilled, then the review was rated as low quality. The reviews rated as critically low quality were excluded. Details of quality assessment are presented in Additional file 1: Table S6.

### Reviews with overlapping primary studies and update of reviews

When two or more reviews studied the association of the same exposure and outcome, it is important to measure the extent of overlap in the primary studies included in the reviews. We used the corrected covered area (CCA) measure to establish the percentage of overlap [105, 106]. CCA less than 5 indicates low overlap, and CCA 10 or above indicates moderate/high overlap. Where reviews had a high overlap, Cochrane review was selected over a non-Cochrane review. If there were no Cochrane reviews, then a review was selected if it had higher AMSTAR quality rating, was conducted recently, included a meta-analysis, or had a larger sample size [105–107]. Further detailed information on dealing with overlapping reviews and update of reviews is provided in Additional file 1.

### Data synthesis

We firstly categorised the outcomes as maternal and foetal/neonatal outcomes. We reported the basic characteristics of the included reviews in tables and indicated which outcomes the review reported and which were retained for the umbrella review after estimating the overlap. The overlap of the reviews was quantified through CCA, and the percentage was calculated. (a) CCA > 10% = two reviews with high overlap (CCA > 10%)—one review was selected to present the results based on the criteria mentioned earlier. The effect estimates from that review were presented as it is or meta-analysed where it was presented separately for cohort and case control studies to get a pooled effect of that outcome. (b) CCA < 10% = (few overlapping primary studies between the reviews). Data from the unique primary studies of the overlapping reviews is extracted, and meta-analysis (random effect) is performed. Data were then extracted (number of exposed, unexposed, and total numbers or odds ratio or risk ratio) from these primary studies. Using a random effect model, meta-analysis was conducted to obtain a pooled effect estimate of the outcome in question. (c) CCA = 0% = (no overlapping primary studies between the reviews). Data from the primary studies of these reviews is extracted, and meta-analysis (random effect) is performed to get the combined effect estimate. The results were synthesised in narrative form, forest plots, and tables. For consistency, the effect sizes were converted to odds ratio where possible [108]. For certain outcomes, the summary estimates could not be converted to odds ratio due to missing data. R and STATA were used for the analysis.

## Results

### Literature search

**Fig. 1:**
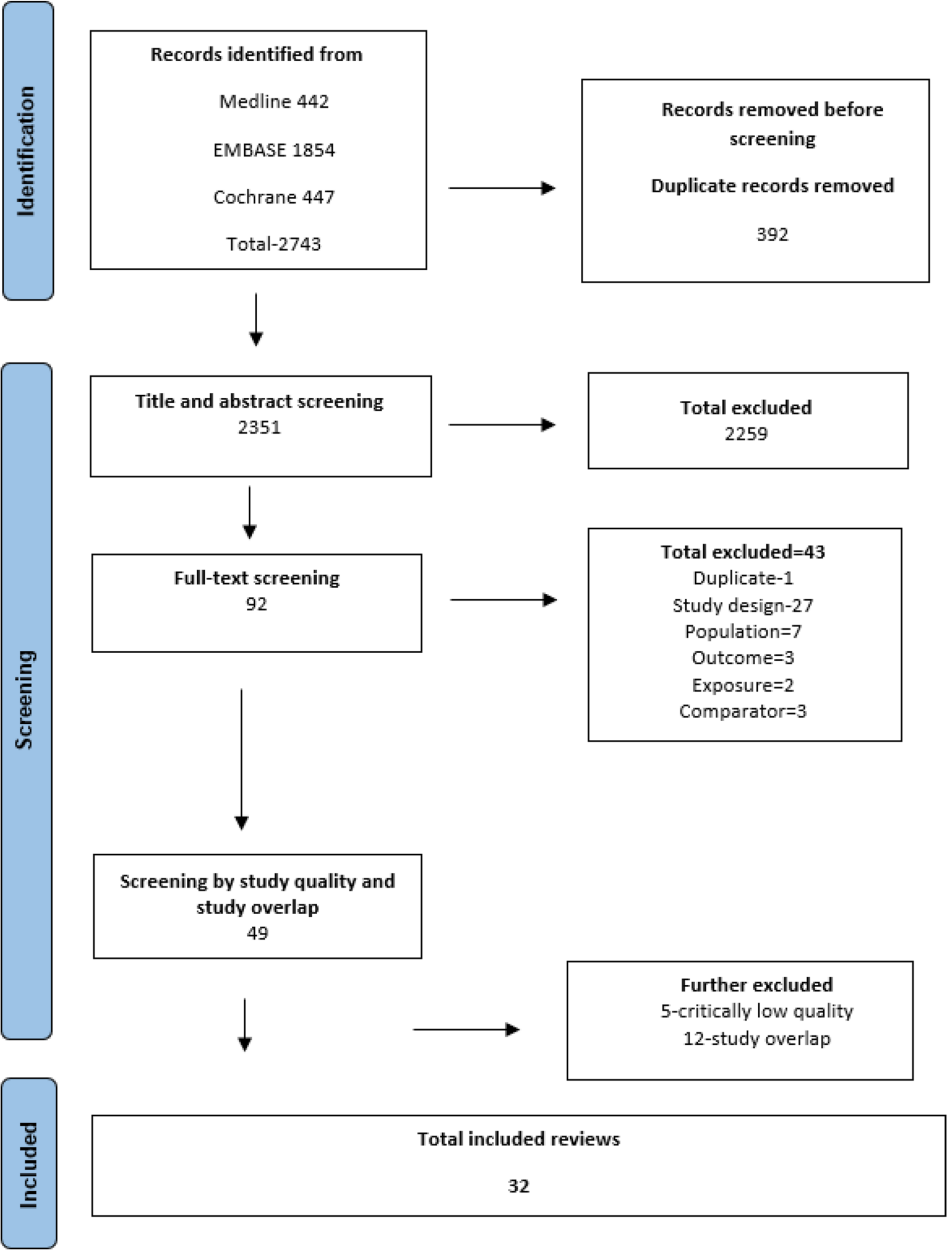
Preferred reporting items for systematic review and meta-analysis (PRISMA) flow diagram

The literature search from Medline, Embase, and Cochrane library of systematic reviews identified 2743 potential reviews. We excluded 392 duplicates. After screening 2351 titles and abstracts, 92 full texts were screened, of which 43 were excluded, with reason for exclusion documented. Forty-nine reviews were initially included for appraisal of the study quality and study overlap. A further 17 reviews were excluded due to critically low study quality and overlaps in the included primary studies. Finally, 32 reviews were included in this umbrella review [29, 30, 32–38, 109–131]. Figure 1 shows the selection process in accordance with the PRISMA flow diagram.

### Quality assessment

Of the 49 reviews that were initially included, five reviews were excluded due to being critically low in quality (assessed using the AMSTAR2 tool). The critical domains—meaning that a review would be rated as high quality review—were as follows: protocol registered before the commencement of the review (item 2), adequacy of the literature search (item 4), justification for excluding individual studies (item 7), risk of bias from individual studies being included in the review (item 9), appropriateness of meta-analytical methods (item 11), consideration of risk of bias when interpreting the results of the review (item 13), and assessment of presence and likely impact of publication bias (item 15) [132]. The five reviews which were excluded did not qualify on at least three of these critical domains [45, 57, 87, 93, 95]. The five reviews which were excluded did not qualify on at least three of these critical domains. In the included systematic reviews, the quality of the primary studies was assessed through the Newcastle–Ottawa scale [132]. The majority of reviews reported moderate to high quality rating of studies.

### Overlapping and non-overlapping association and update of reviews

The overlapping association was noted with most autoimmune conditions reporting various pregnancy outcomes except for myasthenia gravis, multiple sclerosis, and systemic sclerosis, as only one review for each of these conditions was identified. The degree of overlap (CCA) ranged from 0 to 66%. Out of 41 overlapping associations calculated, 34 high overlapping associations with CCA ranging from 12 to 66%. Review was selected in these cases to report the results based on the above-mentioned criteria. Eleven reviews were excluded owing to high overlap [31, 48, 53, 55, 63, 78, 88, 96, 97, 101, 103]. For six associations, the CCA was between 0 and 7%. Random effect meta-analysis was conducted to obtain the pooled estimate from the primary studies from these reviews without double counting. Details of how the citation matrix was created and the CCA calculated are given in Additional file 1: Text S1 [29, 30, 33–35, 38, 41, 45, 48, 53, 55, 63, 66, 78, 88, 96, 97, 103, 106, 108, 110–113, 115–117, 119, 120, 122–125, 127–130, 132–142] and Additional file 1: Tables S7 and S8. After consultation with experts, we decided that none of the reviews required an update. More information is in Additional file 1: Text S1 and Additional file 1: Table S9.

### Summary of the results

Of the 32 systematic reviews included, 30 had completed a meta-analysis, and two synthesised the findings narratively. The characteristics of the included systematic reviews are presented in Table 2, and details are in Additional file 1: Table S11. Figure 3 presents the heatmap of the association of each autoimmune conditions with pregnancy outcomes, highlighting areas with evidence gap, especially for multiple sclerosis and Sjogren’s syndrome (Table 3).

**Table 2:** Formula for corrected cover area

CCA (%) = *N* − *r*/*rc* − *r*	

*CCA* corrected covered area, *N* number of included publications (sum of checked boxes), *r* number of rows (primary studies), *c* number of columns (number of systematic reviews)

**Table 3:** Characteristics of the included systematic reviews reporting the association of autoimmune conditions and adverse pregnancy outcomes

Exposure	Author and year	Outcomes	Number of included primary studies	Quality assessment AMSTAR 2
Axial spondyloarthropathy	Maguire 2020	Pre-eclampsia, gestational diabetes mellitus, caesarean section, small for gestational age, intrauterine growth restriction, preterm birth, low birth weight	18	Moderate
Coeliac disease	Saccone 2016	Small for gestational age, low birth weight	10	Low
	Tersigni 2014	Recurrent miscarriage, small for gestational age	24	Low
	Arvanitakis 2022	Miscarriage, pre-eclampsia caesarean section, postpartum haemorrhage, stillbirth, intrauterine growth restriction, preterm birth	18	Moderate
Inflammatory bowel disease	Cornish 2007	Caesarean section, small for gestational age, stillbirth, preterm birth	13	Moderate
	O’Toole 2015	Caesarean section, small for gestational age, preterm birth, low birth weight	23	Moderate
	Leung 2021	Preterm birth, small for gestational age, low birth weight	72	Moderate
	Talavera 2021	Ectopic pregnancy	5	Moderate
	Tandon 2020	Ectopic pregnancy, miscarriage, caesarean section, gestational hypertension, pre-eclampsia, placenta previa, placental abruption, termination of pregnancy	53	Moderate
Rheumatoid arthritis	Jiamin 2023	Miscarriage, pre-eclampsia, gestational diabetes mellitus, caesarean section, small for gestational age, intrauterine growth restriction, preterm birth, low birth weight, stillbirth	41	Moderate
Multiple sclerosis	Arafa 2021	Pre-eclampsia	8	Moderate
	*Modrego 2021	Preterm birth, low birth weight	17	Low
Myasthenia gravis	*Banner 2021	Caesarean section, preterm birth	32	Low
Psoriasis and psoriatic arthritis	Xie 2021	Psoriasis—pre-eclampsia or eclampsia, gestational hypertension, gestational diabetes mellitus, caesarean section, small for gestational age, intrauterine growth restriction, preterm birth, low birth weight antepartum and postpartum haemorrhage, neonatal mortality Psoriatic arthritis—ectopic pregnancy, pre-eclampsia or eclampsia, gestational hypertension, gestational diabetes mellitus, caesarean section, preterm birth	16	Moderate
Sjögren’s syndrome	Upala 2015	Miscarriage, preterm birth, stillbirth, small for gestational age, intrauterine growth restriction, foetal loss	7	Moderate
	Geng 2022	Miscarriage, preterm birth, low birth weight	9	Moderate
Systemic lupus erythematosus	Bundhun 2017	Ectopic pregnancy, miscarriage, pre-eclampsia, caesarean section, low birth weight, small for gestational age, preterm birth	11	Low
	Dong 2020	Pre-eclampsia	10	Moderate
	He W 2020	Miscarriage, pre-eclampsia, gestational diabetes mellitus, caesarean section, small for gestational age, intrauterine growth restriction, stillbirth, preterm birth, low birth weight, foetal loss	6	Moderate
	Wei 2017	Preterm birth	24	Moderate
Systemic sclerosis	Blagojevic 2020	Miscarriage, pre-eclampsia, intrauterine growth restriction, preterm birth, low birth weight	16	Low
Thyroid autoimmunity (both thyroid peroxidase antibody, and thyroglobulin antibody)	Chen 2011	Miscarriage	22	Low
	HeX 2012	Preterm birth	11	Moderate
	Li M 2016	Preterm birth	18	Low
	Lou 2020	Gestational diabetes mellitus	44	Low
	Dong 2020	Recurrent miscarriage, miscarriage	17	Low
Thyroid peroxidase antibody	Korevaar 2020	Preterm birth	19	Moderate
	Milandi 2020	Postpartum depression	5	Low
	Thangaratinam 2011	Miscarriage, preterm birth	36	Moderate
	Tong 2016	Intrauterine growth restriction, small for gestational age, low birth weight	7	Moderate
	Zhang, 2016	Adverse obstetric outcomes, miscarriage, preterm birth, gestational hypertension, placental abruption, intrauterine growth restriction	7	Low
Type 1 diabetes mellitus	Yu 2017	Pre-eclampsia or eclampsia, gestational hypertension, caesarean section, small for gestational age, intrauterine growth restriction, preterm birth, low birth weight	100	Low

All the studies conducted a meta-analysis except the studies marked (*) are narrative analysis

More information about heterogeneity and publication bias is reported in Additional file 1: Text S1 and Additional file 1: Table 11.

The effect sizes (odds ratios/risk ratios) of the included meta-analysis are shown in Additional file 1: Table S12, and the narrative synthesis conducted by the included reviews is in Additional file 1: Table S13.

### Maternal outcomes

**Fig. 2:**
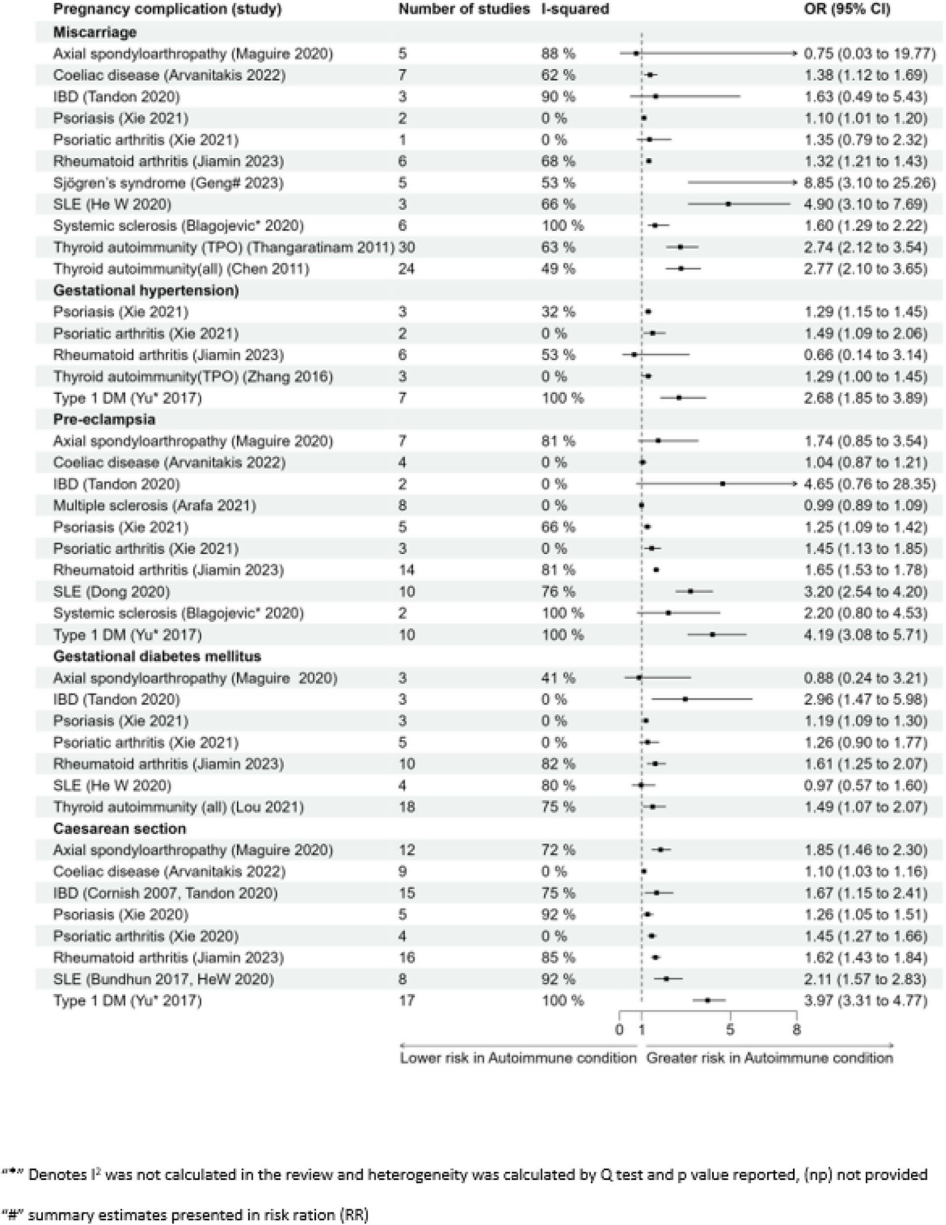
Forest plot for association of pregnancy complications and maternal outcomes

Figure 2 presents the forest plots of effect sizes from the included systematic reviews for maternal outcomes.

#### Ectopic pregnancy

Significant risk of ectopic pregnancy was reported in women with IBD (odds ratio (OR) 1.26 (95% confidence intervals 1.11–1.44)). It was also reported that the risk is similar for women with Crohn’s disease (OR 1.51 (1.21–1.88)) and ulcerative colitis (OR 1.50 (1.00–2.23)) [123]. No significant association was observed in women with coeliac disease (OR 1.21 (0.85–1.71)) or in women with SLE (OR 1.79 (0.57–5.59)) [110].

#### Miscarriage

Higher risk of miscarriage was reported in women with Sjögren’s syndrome relative risk (RR) 8.85 (3.10–25.26) or SLE OR 4.90 (3.10–7.69) respectively [32, 114]. In the presence of thyroid autoimmunity (all antibodies), the risk was almost threefold (OR 2.77 (2.10–3.65)) [111]. Similar risk was reported with only thyroid peroxidase antibody (OR 2.74 (2.12–3.54)) [125]. Significant association of miscarriage was also observed with coeliac disease (OR 1.38 (1.12–1.69)), rheumatoid arthritis (OR 1.32 (1.21, 1.43)), psoriasis (OR 1.10 (1.01–1.20)), and systemic sclerosis (OR 1.60 (1.29–2.22)) [29, 31, 109, 128]. No significant association was reported for miscarriage in women with IBD (OR 1.63 (0.49–5.43)) or psoriatic arthritis (OR 1.35 (0.79–2.32)) [34, 128, 131]. One of the studies reported composite outcome of abortion (spontaneous and therapeutic) in pregnant women with SLE (OR 1.40 (1.20–1.60)) [110].

#### Recurrent pregnancy loss

Women with coeliac disease had almost sixfold higher odds of recurrent pregnancy loss (OR 5.82 (2.3–14.74)). The risk was also reported as almost twofold higher in the presence of thyroid autoimmunity (all) (OR 1.94 (1.43–2.86)) [113, 124].

#### Gestational hypertension

The odds of gestational hypertension in women with T1DM was more than twofold (OR 2.68 (1.85–3.89)) [129]. There were also almost 20–50% higher odds of gestational hypertension in women with psoriatic diseases (psoriasis OR 1.29 (1.15–1.45), psoriatic arthritis OR 1.49 (1.09–2.06), and presence of thyroid autoimmunity (TPO) OR 1.29 (1.00–1.45)) [128, 130].

#### Pre-eclampsia

The odds of developing pre-eclampsia were more than four times greater in women with T1DM (OR 4.19 (3.08–5.71)) [129]. High risk was also reported in women with SLE (OR 3.20 (2.54–4.20)) or systemic sclerosis (OR 2.20 (2.21–4.53)) [29, 30]. Some association was observed in women with rheumatoid arthritis (OR 1.65 (1.53, 1.78)) or psoriatic diseases (psoriasis OR 1.25 (1.09–1.42), psoriatic arthritis OR 1.45 (1.13–1.85)) [31, 128, 131]. No significant association was noted in women with AxSpA (OR 1.74 (0.85–3.54)), coeliac disease (OR 1.04 (0.87–1.21)), or IBD (OR 4.65 (0.76–28.35)) [34, 38, 109, 122]. Another review which collectively evaluated the outcome as the risk of eclampsia and pre-eclampsia reported an increased risk in women with psoriatic diseases (psoriasis OR 1.25 (1.09–1.42), psoriatic arthritis OR 1.45 (1.13–1.85)) [128]. No association was seen between women with multiple sclerosis and occurrence of pre-eclampsia (OR 0.99 (0.89–1.09)) [121].

#### Gestational diabetes mellitus (GDM)

The risk of women developing GDM was almost threefold in women with IBD (OR 2.96 (1.47–5.98)) [34]. Some association was also reported in women with thyroid autoimmunity (all) (OR 1.49 (1.07–2.07)) or psoriasis (OR 1.19 (1.09–1.30)) [119, 128]. Lou et al. reported similar risk of GDM in both women with only thyroglobulin antibodies (OR 1.88 (1.13–3.12)) and only thyroid peroxidase antibodies (OR 1.65 (1.13–2.40)). No significant association was reported for developing GDM in women with AxSpA (OR 0.88 (0.24–3.21)), psoriatic arthritis (OR 1.26 (0.90–1.77)), rheumatoid arthritis (OR 1.61 (1.25, 2.07)), and SLE (OR 0.97 (0.57–1.60)) [31, 32, 38, 128, 131].

#### Placenta previa and placental abruption

No risk of placenta previa and placental abruption was reported in women with thyroid autoimmunity (OR 0.42 (0.12–1.43)) [130].

#### Antepartum haemorrhage/postpartum haemorrhage (APH/PPH)

No significant association was reported for APH/PPH in women with coeliac disease (OR 1.11 (0.96–1.28)) or psoriatic diseases (psoriasis OR 1.22 (0.74–01), psoriatic arthritis OR 0.82 (0.37–1.82) when compared to women without psoriatic diseases [109, 128].

#### Caesarean section (CS)

Risk of delivering via CS is almost twofold to threefold greater in the presence of T1DM (OR 3.97 (3.31–4.77)) and SLE (OR 2.11 (1.57–2.83)) [129]. Women with all other reported autoimmune conditions were reported to have increased chances of delivering via CS compared to women without the autoimmune conditions. The odds are as follows: AxSpA OR 1.85 (1.46–2.30), coeliac disease OR 1.10 (1.03–1.16), IBD OR 1.67 (1.15–2.41), psoriasis OR 1.26 (1.05–1.51), psoriatic arthritis OR 1.45 (1.27–1.66), rheumatoid arthritis OR 1.62 (1.43, 1.84) [31, 34, 38, 109, 110, 112, 128, 131]. It was further reported that there was a significant risk of women delivering through CS with Crohn’s disease (OR 1.65 (1.19–2.29)) but not as much with ulcerative colitis (OR 1.30 (0.86 to 1.96)) [33, 34, 112]. One of the reviews conducted a narrative analysis and reported women with myasthenia gravis are at increased risk of requiring assisted vaginal delivery or CS compared to women without the condition (population size = 854) [37].

#### Postpartum depression

Almost a twofold increased risk of developing postpartum depression in the presence of thyroid autoimmunity (TPO) (OR 2.00 (1.62 to 2.66)) was reported [120].

#### Foetal/neonatal outcomes

**Fig. 3:**
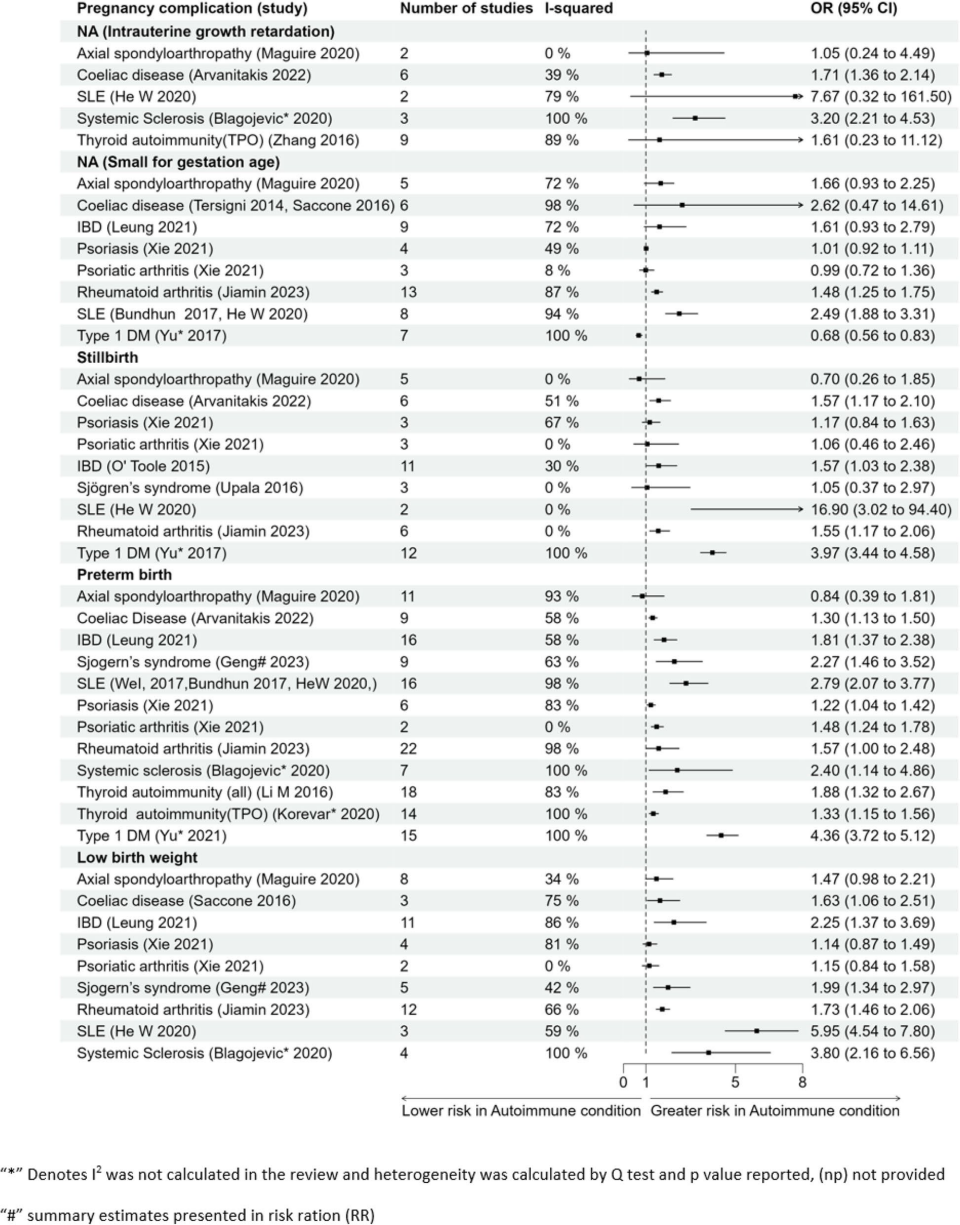
Forest plot for association of pregnancy complications and foetal/neonatal outcomes

It has been observed that there is a higher risk of IUGR, stillbirth, preterm birth, or low birth weight in women with SLE and women with T1DM associated with 32% lower odds of small for gestational age baby as shown in Fig. 3.

#### Intrauterine growth restriction (IUGR)

The evidence is suggestive of almost twofold and threefold risk of IUGR in women with systemic sclerosis or coeliac disease (OR 3.20 (2.21–4.53) OR 1.71 (1.36–2.14) respectively) [29, 109, 122], whereas no significant association was observed in women with AxSpA (OR 1.05 (0.24–4.49)), SLE (OR 7.67 (0.32–161.50)), thyroid autoimmunity (TPO) (OR 1.61 (0.23–11.12)) [32, 38, 130]. Tong et al. reported composite outcome IUGR (including IUGR, foetal growth restriction, and low birth weight) in women with thyroid autoimmunity (TPO) which was reported as OR 1.57 (0.77–3.18) [126] when compared to women without thyroid autoimmunity [126].

#### Small for gestational age (SGA)

There is an increased risk of small for gestational age in women with SLE (OR 2.49 (1.88–3.31)) [32, 110]. A significant association is reported with rheumatoid arthritis (OR 1.48 (1.25, 1.75)) [131]. No significant association was reported in women with AxSpA (OR 1.66 (0.93–2.25)), coeliac disease (OR 2.62 (0.47–14.61)), IBD (OR 1.61 (0.93–2.79)), psoriatic diseases (psoriasis OR 1.01 (0.92–1.11), psoriatic arthritis OR 0.99 (0.72–1.36)) [38, 122, 124, 128], whereas women with T1DM have a lower effect with OR 0.68 (0.56–0.83) [129].

#### Stillbirth

There was a very high risk of stillbirth in women with SLE, although with wide confidence intervals (OR 16.90 (3.02–94.40)) [32] and almost a fourfold higher risk of stillbirth for women with T1DM (OR 3.97 (3.44–4.58)) [129]. There was a significantly higher odds for women with rheumatoid arthritis (OR 1.99 1.55 (1.17, 2.06)), coeliac disease (OR 1.57 (1.17–2.10)), and IBD (OR 1.57 (1.03–2.38)) [33, 97, 109, 131]. There was no significant association with stillbirth in women with and AxSpA (OR 0.70 (0.26–1.85)), psoriatic diseases (psoriasis OR 1.17 (0.84–1.63), psoriatic arthritis OR 1.06 (0.46–2.46)), or Sjögren’s syndrome (OR 1.05 (0.37–2.97)) [38, 127, 128]. Two separate reviews reported significantly higher odds of stillborn/neonatal/perinatal death as a composite outcome in women with SLE (OR 1.75 (1.47–2.37)) and in women with rheumatoid arthritis (OR 1.38 (1.09–1.74)) [31, 110].

#### Preterm birth

There were more than fourfold higher odds of preterm birth for women with T1DM (OR 4.36 (3.72–5.12)). The odds of preterm birth were also higher in women with SLE (OR 2.79 (2.07–3.77)) and in women with Sjögren’s syndrome (RR 2.27 (1.46–3.52)) [32, 110, 114, 129]. There was a significant association for preterm birth in women with coeliac disease (OR 1.30 (1.13–1.50)), IBD (OR 1.81 (1.37–2.38)), psoriatic diseases (psoriasis OR 1.22 (1.04–1.42), psoriatic arthritis OR 1.48 (1.24–1.78)), rheumatoid arthritis (OR 1.57 (1.00, 2.48)), and systemic sclerosis (OR 2.40 (1.14–4.86)) and for thyroid autoimmunity (all) (OR 1.88 (1.32–2.67) and thyroid autoimmunity (TPO) (OR 1.33 (1.15–1.56)) when compared to women without these conditions [29, 31, 32, 35, 97, 109, 110, 117, 128, 131]. Korevar et al. further reported higher odds of preterm birth for thyroid autoimmunity (TPO) positive women (OR 1.33 (1.15–1.56)) and no significant association with thyroid autoimmunity (TgAb) positive women (OR 0.88 (0.64 to 1.20)) when studied separately [116]. Two separate reviews analysed the odds of preterm birth with multiple sclerosis (sample size *n* = 6230) and myasthenia gravis (*n* = 854) in a narrative analysis and did not find any significant association [37, 121].

#### Low birth weight

The odds of giving birth to a baby with a low birth weight (< 2500 g) was 4–6 times higher in women with SLE or systemic sclerosis (OR 5.95 (4.54–7.80) and OR 3.80 (2.16–6.56), respectively) [29, 32]. There was a significant association for women with IBD (OR 2.25 (1.37–3.69)), coeliac disease (OR 1.63 (1.06–2.51)), rheumatoid arthritis (OR 1.73 (1.46, 2.06)), or Sjögren’s syndrome (RR 1.99 (1.34–2.97)) [31, 114, 117, 122, 131]. There were higher odds of having a low-birth-weight infant for women with Crohn’s disease (OR 2.82 (1.42–5.60)), but the association was not significant for women with ulcerative colitis (OR 1.66 (0.48–5.66)) [33, 34, 112]. The association for women with AxSpA (OR 1.47 (0.98–2.21)) or psoriatic diseases (psoriasis OR 1.14 (0.87–1.49), psoriatic arthritis OR 1.15 (0.84–1.58)) [38, 128] and low birth weight was not significant. Another review reported the odds of low birth weight with multiple sclerosis in a narrative analysis (*n* = 635), and only one study reported a higher risk of low birth weight in women with multiple sclerosis [121].

#### Neonatal mortality

SLE was associated with a much greater odds of neonatal mortality (OR 8.32 (5.23–13.22)). There was also a significantly higher odds of neonatal mortality for women with T1DM OR 2.26 (1.74–2.95) or Sjögren’s syndrome OR 1.77 (1.28–2.46). Psoriasis was not significantly related to neonatal mortality (OR 1.13 (0.90–1.43)) (Table 4) [5, 42, 43].

**Table 4:**
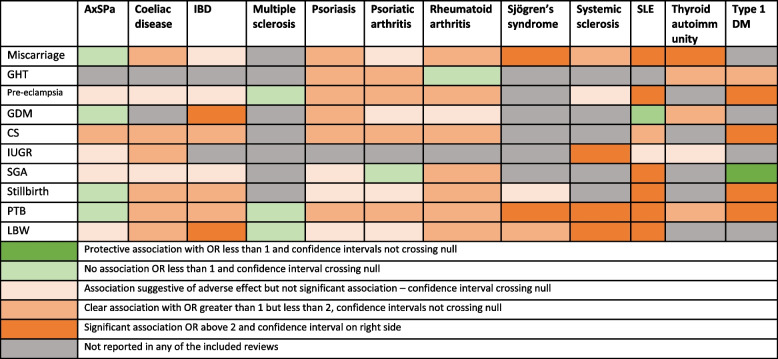
Heat map of the association of autoimmune conditions and adverse pregnancy outcomes

*GHT* gestational hypertension, *GDM* gestational diabetes mellitus, *CS* caesarean section, *IUGR* intrauterine growth restriction, *SGA* small for gestational age, *PTB* preterm birth, *LBW* low birth weight, *AxSpA* axial spondyloarthropathy, *IBD* inflammatory bowel disease, *SLE* systemic lupus erythematosus, *T1DM* type 1diabetes mellitus

## Discussion

### Main findings

Results from this umbrella review agree with the previous literature and showed that women with SLE, T1DM, and systemic sclerosis have a higher risk for a number of adverse pregnancy outcomes and also that women with T1DM were less likely to have a baby that was small for gestational age. Women with SLE had a greater risk of miscarriage, pre-eclampsia, small for gestational age, preterm birth, stillbirth, and low birth weight. Women with T1DM were more likely to develop pre-eclampsia, caesarean section, preterm birth, and still birth, and women with systemic sclerosis had a higher risk of pre-eclampsia, IUGR, low birth weight, and preterm birth. There was also a greater risk of miscarriage for women with thyroid autoimmunity and for women with Sjögren’s syndrome. Women with IBD had a higher risk of GDM and low birth weight babies. The associations of AxSpA, coeliac disease, psoriatic diseases, multiple sclerosis, and myasthenia gravis with certain adverse pregnancy outcomes were not clearly evident. There was no evidence of significant association of multiple sclerosis with risk of pre-eclampsia and low birth weight, for SLE with risk of GDM, and for AxSpA with preterm birth, stillbirth, or GDM. There are various knowledge gaps identified. No systematic reviews identified for few autoimmune conditions (Grave’s disease, Hashimoto’s thyroiditis, vitiligo, Addison’s disease, alopecia areata). Further few adverse pregnancy outcomes like ectopic pregnancy, obstetric haemorrhage, perineal tears, postpartum depression, or psychosis are not widely studied.

### Strengths and limitations

The main strength of this umbrella review is the comprehensive search strategy with no language or time restrictions and searches that were repeated periodically to include newly published systematic reviews. Secondly, we included a wide range of autoimmune conditions and adverse pregnancy outcomes which were identified by scoping searches of the literature and after consultation with subject experts, making this review more relevant and useful. Definitions of the outcomes for inclusion were predefined so that these were uniform across the systematic reviews [43]. The summary estimates have been converted to odds ratios where possible to facilitate the interpretation of the results.

The main limitation of this umbrella review was that we were not able to include certain autoimmune conditions and their effects on pregnancy outcomes if there were no systematic reviews. For example, there were primary studies reporting the association of vitiligo with adverse pregnancy outcome, but no systematic review has been conducted on this topic [143]. None of the included reviews were rated high quality following the AMSTAR2 checklist and were all either moderate or low quality, which affected the overall certainty of the evidence in this umbrella review. There was high heterogeneity between the reviews. Most of the included reviews had multiple outcomes, and in many instances, the sample size of the primary studies used for measuring the outcome in the systematic review was small, thus providing large variation in the effect size. Some reviews restricted the search strategy (e.g. time period or type of study), and the number of studies included in the meta-analysis was small meaning there could be some publication bias. Even though we attempted to include the majority of common autoimmune conditions, not all of them could be included (i.e. antiphospholipid syndrome and dermatomyositis) [144, 145]. Another limitation is that this review cannot address the effects of co-morbidities on outcomes, for example, increased risk of antiphospholipid syndrome (APS) in SLE which might be the cause of the outcomes rather than SLE directly [146]. For the outcome caesarean delivery, the reviews did not specify or analysed the indications, and therefore, it remains unclear if the increased risk was caused by the disease activity, medications, or other factors including patient or physician preference. This umbrella review provides comprehensive evidence on the adverse pregnancy outcomes in women with autoimmune conditions. No systematic reviews identified for thyroid diseases Grave’s disease or Hashimoto’s thyroiditis; however, there was an extensive research for thyroid autoimmunity and effects on pregnancy outcome. Hence, this review has incorporated thyroid autoimmunity as a whole and consolidate the findings in this area. Other diseases not included are vitiligo, Addison’s disease, or alopecia areata for which there are no prior systematic reviews. Two of the retrospective cohort studies identified exploring the association of vitiligo with spontaneous miscarriage reported significant association with OR 1.25 (1.14–1.36) and aHR 1.16 (1.09–1.25) when compared with women without vitiligo [147, 148]. Other studies showed no significant association of vitiligo with risk of preterm birth, GDM, or stillbirth [143, 147, 148]. Two different studies reported Addison’s disease was associated with almost twofold increase in the risk of preterm birth and caesarean section compared to women without the condition [149, 150]. One of the cohort studies showed that women with alopecia areata had a greater risk of adverse pregnancy outcomes including spontaneous miscarriage or ectopic pregnancy with OR 1.09 (1.00–1.18) and OR 1.28 (1.13–1.46) respectively [151]. There are also other rare autoimmune conditions that were beyond the scope of this review (e.g. myositis, vasculitis) which may be associated with increased risk of adverse pregnancy outcomes [152]. Small for gestational age, pregnancy loss, IUGR, preeclampsia, and preterm birth and miscarriage has been associated with the connective tissue diseases [153–155]. The drugs used for the treatment of these autoimmune conditions such as biological and conventional synthetic disease-modifying antirheumatic drugs (DMARDs), oral glucocorticoids, and non-steroidal anti-inflammatory drugs can also affect the pregnancy outcomes, and various studies have been conducted to estimate which medication pose higher risk than others [156–158]. However, active inflammatory disease has been shown to increase the risk of miscarriage, preterm delivery, small for gestational age babies, and preterm delivery [159]. Understanding the contribution of disease activity and the effect of pregnancy on the disease and vice versa has not been addressed in this review and will require further research [160]. Similarly, understanding the risk of these medications and how they contribute towards adverse pregnancy outcomes need to be researched and guidelines need to be formulated [161]. Furthermore, the effect of the autoimmunity in women during pregnancy also might affect the offspring, and they have been linked with learning disabilities, dyslexia, and autism [162–164]. Further research of the associations of these conditions with pregnancy outcomes is therefore warranted.

### Clinical and research implications

The primary objective of this review is to consolidate the findings from the systematic reviews, present what is known and to identify gaps in the research. This review is aimed primarily at a range of health professionals who are directly involved with managing women with these autoimmune conditions who are planning a pregnancy or are pregnant. This includes specialists like rheumatologists, endocrinologists, gynaecologists, obstetricians, rheumatology nurses/allied health professionals, and general practitioners as well as the patients themselves. This document may also be a useful resource for policymakers to evaluate the present guidelines and propose further recommendations and help to service provider to risk stratify women and decide on the referral threshold or best care pathway. Since many adverse pregnancy outcomes are shared across conditions, then this provides argument for (i) research to identify if there is a common mechanism which could lead to newer treatments and (ii) guidelines which are relevant across different conditions. This review not only identify gaps in the research but also is especially of value to non-specialist obstetricians who might see a number of different autoimmune diseases within the same clinic.

There are currently limited guidelines for preconception and pregnancy care for a limited number of autoimmune conditions. For example, there is a best practice guideline from a UK multispecialty working group for myasthenia in pregnancy [165] and the National Institute for Health and Care Excellence (NICE) guidelines for T1DM in pregnancy, UK consensus on pregnancy in multiple sclerosis: ‘Association of British Neurologists’ guidelines [166, 167]. There are few guidelines on prescribing medication in pregnancy for women with rheumatic diseases and other musculoskeletal diseases like SLE or antiphospholipid syndrome [168–170]. There are guidelines around prescribing in pregnancy but less so around other aspects of autoimmune disease. Most guidelines focus on safety of medication (which is applicable to all diseases), but the unmet need is in management of these patients in general. But there is a need for the development of more evidence-based standardised guidelines for a wider range of autoimmune conditions since they have shared outcomes to help clinicians and women with decision making for managing these conditions before planning a pregnancy and while pregnant [12, 25, 28, 171–173]. It has been established that preconception care for chronic disease can help improve pregnancy outcome such as miscarriages in women with autoimmune rheumatic conditions and low birth weight in women with IBD [174]. Preconception counselling and risk stratification are possible tools that may help reduce the risk of complications during pregnancy by ensuring disease stability/control prior to conception. Antenatal care, for example, use of low dose aspirin to prevent and predictive/diagnostic biomarkers for pre-eclampsia in women with SLE or T1DM, for example, predictive biomarkers for pre-eclampsia in women with SLE or T1DM, and identification of placental insufficiency with foetal growth restriction in order to decide the best timing for delivery may also lead to improved outcomes [171, 175–177]. Many autoimmune conditions like Addison’s disease, alopecia areata, and vitiligo could not be reported due to no systematic reviews conducted is reported as a gap in research. Future research should address the evidence gaps identified in this umbrella review, for example, for women with multiple sclerosis and Sjogren’s syndrome.

## Conclusions

This review has provided comprehensive summary of the current evidence of the association of pregnancy outcomes in women with autoimmune conditions and identified gaps that need further research. Given the potential adverse outcomes, more clinical guidelines need to be developed to guide the preconception and maternity care for pregnant women with autoimmune conditions.

### Supplementary Information

**Additional file 1: Text S1.** Details of the overlappling reviews, quality assessment and heterogeneity. **Table S1.** PRIOR checklist (Preferred Reporting Items for Overviews of Reviews. **Table S2.** Deviations from protocol. **Table S3.** Search Strategy OVID MEDLINE. **Table S4.** List of excluded studies. **Table S5.** Data extraction form. **Table S6. **Quality Assessment of included studies using AMSTAR 2 Tool. **Table S7.1-7.9.** Citation matrices for reviews with overlapping association. **Table S8.** Overlapping and non-overlapping association. **Table S9.** Forest plots of the combined review. **Table S10.** Evaluation of need to update the reviews. **Table S11.** General characteristics of systematic reviews included in the umbrella review. **Table S12.** Tabular presentation of findings: Meta-analysis. **Table S13.** Tabular presentation of findings: Narrative synthesis.

## Data Availability

All data generated or analysed during this study are included in this published article and its supplementary information files.

## Abbreviations

aHR: Adjusted hazard ratio
AMSTAR 2: Assessment of multiple systematic reviews version 2
APH: Antepartum haemorrhage
APS: Antiphospholipid syndrome
AxSpA: Axial spondyloarthropathy
CCA: Corrected covered area
CS: Caesarean section
DMARDs: Disease-modifying antirheumatic drugs
GDM: Gestational diabetes mellitus
GHT: Gestational hypertension
HELLP: Haemolysis, elevated liver enzymes and low platelet syndrome
IBD: Inflammatory bowel disease
IUGR: Intrauterine growth retardation
JBI: Joanna Briggs Institute
LBW: Low birth weight
MESH: Medical Subject Headings
MS: Multiple sclerosis
NICE: National Institute for Health and Care Excellence
OR: Odds ratio
PPH: Postpartum haemorrhage
PPIE: Patient and public involvement and engagement
PRIOR: Preferred Reporting Items for Overviews of Reviews
PRISMA: Preferred reporting items for systematic review and meta-analysis
RR: Risk ratio
SGA: Small for gestational age
SLE: Systemic lupus erythematosus
T1DM: Type 1 diabetes mellitus
TgAb: Thyroglobulin antibody
TPO: Thyroid peroxidase antibody

## References

[1] Invernizzi P, Pasini S, Selmi C, Gershwin ME, Podda M (2009). Female predominance and X chromosome defects in autoimmune diseases. J Autoimmun.

[2] Jacobson DL, Gange SJ, Rose NR, Graham NM (1997). Epidemiology and estimated population burden of selected autoimmune diseases in the United States. Clin Immunol Immunopathol.

[3] Eaton WW, Rose NR, Kalaydjian A, Pedersen MG, Mortensen PB (2007). Epidemiology of autoimmune diseases in Denmark. J Autoimmun.

[4] Angum F, Khan T, Kaler J, Siddiqui L, Hussain A (2020). The prevalence of autoimmune disorders in women: a narrative review. Cureus..

[5] Thomas SL, Griffiths C, Smeeth L, Rooney C, Hall AJ (2010). Burden of mortality associated with autoimmune diseases among females in the United Kingdom. Am J Public Health.

[6] Gleicher N, Barad DH (2007). Gender as risk factor for autoimmune diseases. J Autoimmun.

[7] Ngo ST, Steyn FJ, McCombe PA (2014). Gender differences in autoimmune disease. Front Neuroendocrinol.

[8] Smith-Bouvier DL, Divekar AA, Sasidhar M, Du S, Tiwari-Woodruff SK, King JK (2008). A role for sex chromosome complement in the female bias in autoimmune disease. J Exp Med.

[9] Talal N (1992). Sjögren’s syndrome: historical overview and clinical spectrum of disease. Rheum Dis Clin North Am.

[10] Van Den Hoogen F, Khanna D, Fransen J, Johnson SR, Baron M, Tyndall A (2013). 2013 classification criteria for systemic sclerosis: an American College of Rheumatology/European League against Rheumatism collaborative initiative. Arthritis Rheum.

[11] Desai MK, Brinton RD (2019). Autoimmune disease in women: endocrine transition and risk across the lifespan. Front Endocrinol.

[12] De Carolis S, Moresi S, Rizzo F, Monteleone G, Tabacco S, Salvi S (2019). Autoimmunity in obstetrics and autoimmune diseases in pregnancy. Best Pract Res Clin Obstet Gynaecol.

[13] Walsh SJ, Rau LM (2000). Autoimmune diseases: a leading cause of death among young and middle-aged women in the United States. Am J Public Health.

[14] Wang L, Wang FS, Gershwin ME (2015). Human autoimmune diseases: a comprehensive update. J Intern Med.

[15] Davidson A, Diamond B (2001). Autoimmune diseases. N Engl J Med.

[16] Persson L, Carney Almroth BM, Collins CD, Cornell S, de Wit CA, Diamond ML (2022). Outside the safe operating space of the planetary boundary for novel entities. Environ Sci Technol.

[17] Arnaud L, Mertz P, Gavand P-E, Martin T, Chasset F, Tebacher-Alt M (2019). Drug-induced systemic lupus: revisiting the ever-changing spectrum of the disease using the WHO pharmacovigilance database. Ann Rheum Dis.

[18] Thorburn AN, Macia L, Mackay CR (2014). Diet, metabolites, and “western-lifestyle” inflammatory diseases. Immunity.

[19] Inter-Organization Programme for the Sound Management of Chemicals, World Health Organization. Principles and methods for assessing autoimmunity associated with exposure to chemicals. World Health Organization; 2006.

[20] Sener AG, Afsar I (2012). Infection and autoimmune disease. Rheumatol Int.

[21] Garbarino S, Lanteri P, Bragazzi NL, Magnavita N, Scoditti E (2021). Role of sleep deprivation in immune-related disease risk and outcomes. Communications biology.

[22] Dellaripa PF, Bush T, Miller FW, Feldman CH. The climate emergency and the health of our patients: the role of the rheumatologist. Arthritis Rheumatol (Hoboken, NJ). 2023;75(1):1. 10.1002/art.42279 PMC9794628 35762821

[23] Vojdani A (2014). A Potential link between environmental triggers and autoimmunity. Autoimmune Diseases.

[24] Piccinni M-P, Lombardelli L, Logiodice F, Kullolli O, Parronchi P, Romagnani S (2016). How pregnancy can affect autoimmune diseases progression?. Clin Mol Allergy.

[25] Adams Waldorf KM, Nelson JL (2008). Autoimmune disease during pregnancy and the microchimerism legacy of pregnancy. Immunol Invest.

[26] Popescu MR, Dudu A, Jurcut C, Ciobanu AM, Zagrean AM, Panaitescu AM (2020). A broader perspective on anti-Ro antibodies and their fetal consequences-a case report and literature review. Diagnostics (Basel)..

[27] Bove RM, Houtchens MK (2022). Pregnancy management in multiple sclerosis and other demyelinating diseases. Continuum (Minneap Minn).

[28] Stransky OM, Wolgemuth T, Kazmerski T, Chodoff A, Borrero S, Talabi MB (2021). Contraception decision-making and care among reproductive-aged women with autoimmune diseases. Contraception.

[29] Blagojevic J, AlOdhaibi KA, Aly AM, Bellando-Randone S, Lepri G, Bruni C (2020). Pregnancy in systemic sclerosis: results of a systematic review and metaanalysis. J Rheumatol.

[30] Dong Y, Yuan F, Dai Z, Wang Z, Zhu Y, Wang B (2020). Preeclampsia in systemic lupus erythematosus pregnancy: a systematic review and meta-analysis. Clin Rheumatol.

[31] Huang W, Wu T, Jin T, Zhang Y, Wang J, Qi J, et al. Maternal and fetal outcomes in pregnant women with rheumatoid arthritis: a systematic review and meta-analysis. Clin Rheumatol. 2022;42(3):855–70. 10.1007/s10067-022-06436-0 36357630

[32] He WR, Wei H (2020). Maternal and fetal complications associated with systemic lupus erythematosus: an updated meta-analysis of the most recent studies (2017–2019). Medicine.

[33] O’Toole A, Nwanne O, Tomlinson T (2015). Inflammatory bowel disease increases risk of adverse pregnancy outcomes: a meta-analysis. Dig Dis Sci.

[34] Tandon P, Govardhanam V, Leung K, Maxwell C, Huang V (2020). Systematic review with meta-analysis: risk of adverse pregnancy-related outcomes in inflammatory bowel disease. Aliment Pharmacol Ther.

[35] Wei S, Lai K, Yang Z, Zeng K (2017). Systemic lupus erythematosus and risk of preterm birth: a systematic review and meta-analysis of observational studies. Lupus.

[36] Arafa A, Wang X, Dong JY, Eshak ES (2021). Does multiple sclerosis increase the risk of preeclampsia? A systematic review and meta-analysis. Hypertens Pregnancy.

[37] Banner H, Niles KM, Ryu M, Sermer M, Bril V, Murphy KE (2022). Myasthenia Gravis in pregnancy: systematic review and case series. Obstet Med.

[38] Maguire S, O’Dwyer T, Mockler D, O’Shea F, Wilson F (2020). Pregnancy in axial spondyloarthropathy: a systematic review & meta-analysis. Semin Arthritis Rheum.

[39] Papatheodorou S (2019). Umbrella reviews: what they are and why we need them. Eur J Epidemiol.

[40] Institute TJB (2014). Joanna Briggs Institute Reviewers’ Manual: 2014 edition/Supplement.

[41] Gates M, Gates A, Pieper D, Fernandes RM, Tricco AC, Moher D (2022). Reporting guideline for overviews of reviews of healthcare interventions: development of the PRIOR statement. BMJ.

[42] National stem cell foundation, https://nationalstemcellfoundation.org/glossary/autoimmune-disease/. Accessed Nov 2022.

[43] Singh M, Crowe F, Thangaratinam S, Abel KM, Black M, Okoth K (2022). Association of pregnancy complications/risk factors with the development of future long-term health conditions in women: overarching protocol for umbrella reviews. BMJ Open.

[44] Haddaway NR, Land M, Macura B (2017). A little learning is a dangerous thing”: a call for better understanding of the term ‘systematic review. Environ Int.

[45] Andersen ML, Jølving LR, Stenager E, Knudsen T, Nørgård BM (2023). Maternal multiple sclerosis and health outcomes among the children: a systematic review. Clin Epidemiol.

[46] Balsells M, Garcia-Patterson A, Gich I, Corcoy R (2009). Maternal and fetal outcome in women with type 2 versus type 1 diabetes mellitus: a systematic review and metaanalysis. J Clin Endocrinol Metab.

[47] Bansal S, Okoye O, Rajora N (2020). Pregnancy and lupus nephritis in developing countries: a systematic review. Saudi J Kidney Dis Transpl.

[48] Bobotsis R, Gulliver W, Monaghan K, Lynde C, Fleming P (2016). Psoriasis and adverse pregnancy outcomes: a systematic review of observational studies. Br J Dermatol.

[49] Bundhun PK, Soogund MZS, Huang F (2018). Arterial/venous thrombosis, fetal loss and stillbirth in pregnant women with systemic lupus erythematosus versus primary and secondary antiphospholipid syndrome: a systematic review and meta-analysis. BMC Pregnancy Childbirth.

[50] Cellini M, Santaguida MG, Stramazzo I, Capriello S, Brusca N, Antonelli A (2020). Recurrent pregnancy loss in women with Hashimoto’s thyroiditis with concurrent non-endocrine autoimmune disorders. Thyroid.

[51] Chaudhary H, Ahluwalia J, Kumar R, Kumar N, Gupta A, Suri D (2019). Pregnancy outcomes in a long-term follow-up cohort of pediatric onset systemic lupus erythematosus (PSLE) at a tertiary care center in North-West India. Ann Rheum Dis.

[52] Crawford NM, Steiner AZ (2016). Thyroid autoimmunity and reproductive function. Sem Reprod Med.

[53] Dama M, Steiner M, Lieshout RV (2016). Thyroid peroxidase autoantibodies and perinatal depression risk: a systematic review. J Affect Disord.

[54] Derakhshan A, Peeters RP, Taylor PN, Bliddal S, Carty DM, Meems M (2020). Association of maternal thyroid function with birthweight: a systematic review and individual-participant data meta-analysis. Lancet Diabetes Endocrinol.

[55] Dong Y, Dai Z, Wang Z, Wang H, Yuan F, Zhu Y (2019). Risk of gestational diabetes mellitus in systemic lupus erythematosus pregnancy: a systematic review and meta-analysis. BMC Pregnancy Childbirth.

[56] Essouma M, Nkeck JR, Bigna JJ, Nkoro GA, Ralandison S, Hachulla E (2020). Outcomes of systemic lupus erythematosus pregnancies and associated factors in sub-Saharan Africa: a systematic scoping review. Lupus Sci Med.

[57] Finkelsztejn A, Brooks JB, Paschoal FM, Fragoso YD (2011). What can we really tell women with multiple sclerosis regarding pregnancy? A systematic review and meta-analysis of the literature. BJOG..

[58] Foulon A, Dupas JL, Sabbagh C, Chevreau J, Rebibo L, Brazier F (2017). Defining the most appropriate delivery mode in women with inflammatory bowel disease: a systematic review. Inflamm Bowel Dis.

[59] Gizzo S, Patrelli TS, Rossanese M, Noventa M, Berretta R, Di Gangi S (2013). An update on diabetic women obstetrical outcomes linked to preconception and pregnancy glycemic profile: a systematic literature review. TheScientificWorldJournal.

[60] Gonzalez-Blanco C, Chico A, Gich I, Corcoy R (2010). Glycaemic control and pregnancy outcomes in women with type 1 diabetes: a systematic review and meta-analysis comparison between lispro and regular insulin. Diabetologia..

[61] Grygiel-Gorniak B, Masiero E, Nevaneeth BC, Jojy MM. Rheumatic diseases in reproductive age—the possibilities and the risks. Reprod Sci. 2023;30(1):111–23. 10.1007/s43032-022-00901-6 35359225

[62] Hage MP, Azar ST (2012). The link between thyroid function and depression. J Thyroid Res..

[63] Hamroun S, Hamroun A, Bigna JJ, Allado E, Forger F, Molto A (2020). Fertility and pregnancy outcomes in women with spondyloarthritis: a systematic review and meta-analysis. Ann Rheum Dis.

[64] Hashash JG, Kane S (2015). Pregnancy and inflammatory bowel disease. Gastroenterology and Hepatology.

[65] Hayslett JP (1992). The effect of systemic lupus erythematosus on pregnancy and pregnancy outcome. Am J Reprod Immunol.

[66] He H, Jing S, Gong F, Tan YQ, Lu GX, Lin G (2016). Effect of thyroid autoimmunity per se on assisted reproduction treatment outcomes: a meta-analysis. Taiwan J Obstet Gynecol.

[67] He H, Jing S, Lin G, Lu GX (2015). Effect of thyroid autoantibodies per se on pregnancy outcomes in euthyroid women undergoing IVF/ICSI. Hum Reprod..

[68] Houtchens MK, Edwards NC, Phillips AL (2016). A review of observational studies of women with MS and pregnancy. Mult Scler.

[69] Ideguchi H, Ohno S, Uehara T, Ishigatsubo Y (2013). Pregnancy outcomes in Japanese patients with SLE: retrospective review of 55 pregnancies at a university hospital. Clin Rev Allergy Immunol.

[70] Jaffar F, Laycock K, Huda MSB. Type 1 diabetes in pregnancy: a review of complications and management. Curr Diabetes Rev. 2022;18(7):49–63. 10.2174/1573399818666211105124829 34749617

[71] Jia M, Wu Y, Lin B, Shi Y, Zhang Q, Lin Y (2019). Meta-analysis of the association between maternal subclinical hypothyroidism and gestational diabetes mellitus. Int J Gynaecol Obstet.

[72] Kane S (2003). Inflammatory bowel disease in pregnancy. Gastroenterol Clin North Am.

[73] Kent NL, Young SL, Akison LK, Cuffe JSM (2021). Is the link between elevated TSH and gestational diabetes mellitus dependant on diagnostic criteria and thyroid antibody status: a systematic review and meta-analysis. Endocrine.

[74] Kim MA, Kim YH, Chun J, Lee HS, Park SJ, Cheon JH (2021). The influence of disease activity on pregnancy outcomes in women with inflammatory bowel disease: a systematic review and meta-analysis. J Crohn’s Colitis.

[75] Lamah M, Scott HJ (2002). Inflammatory bowel disease and pregnancy. Int J Colorectal Dis.

[76] Laube R, Tran Y, Paramsothy S, Leong RW (2021). Assisted reproductive technology in Crohn’s disease and ulcerative colitis: a systematic review and meta-analysis. Am J Gastroenterol.

[77] Leiva P, Schwarze JE, Vasquez P, Ortega C, Villa S, Crosby J (2017). There is no association between the presence of anti-thyroid antibodies and increased reproductive loss in pregnant women after ART: a systematic review and meta-analysis. JBRA Assist Reprod.

[78] Li M, Wang SW, Huang S, Mao Y (2016). Relationship between the thyroid autoimmunity and the risk of preterm birth in pregnant women: a meta-analysis. Zhonghua Fu Chan Ke Za Zhi.

[79] Lopez-Leon S, Geissbuehler Y, Sabido M, Turkson M, Wahlich C, Morris J (2020). A systematic review and meta-analyses of pregnancy and fetal outcomes in women with multiple sclerosis. IMI2 conception. Mult Scler J.

[80] McDonald EG, Bissonette L, Ensworth S, Dayan N, Clarke AE, Keeling S (2018). Monitoring of systemic lupus erythematosus pregnancies: a systematic literature review. J Rheumatol.

[81] Meissner Y, Rudi T, Fischer-Betz R, Strangfeld A (2021). Pregnancy in women with psoriatic arthritis: a systematic literature review of disease activity and adverse pregnancy outcomes. Semin Arthritis Rheum.

[82] Mintziori G, Anagnostis P, Toulis KA, Goulis DG (2012). Thyroid diseases and female reproduction. Minerva Med.

[83] Mintziori G, Tarlatzis BC, Goulis DG (2016). The impact of thyroid autoimmunity on IVF/ICSI outcome: re-evaluation of the findings. Hum Reprod Update.

[84] Mokbel A, Lawson DO, Farrokhyar F (2021). Pregnancy outcomes in women with ankylosing spondylitis: a scoping literature and methodological review. Clin Rheumatol.

[85] Moroni G, Calatroni M, Ponticelli C. The impact of preeclampsia in lupus nephritis. Exp Rev Clin Immunol. 2022;18(6):625–37. 10.1080/1744666X.2022.2074399 35510378

[86] Munoz Munoz C, Ahmed K, Thomas M, Cohen H, Alijotas-Reig J, Giles I (2022). Comparing pregnancy outcomes in patients with criteria and non-criteria autoimmune disease: a systematic review. Lupus.

[87] Nazarpour S, Ramezani Tehrani F, Simbar M, Azizi F (2016). Thyroid autoantibodies and the effect on pregnancy outcomes. J Obstet Gynaecol.

[88] Negro R (2011). Thyroid autoimmunity and pre-term delivery: brief review and meta-analysis. J Endocrinol Invest.

[89] Ogallar MAD, Cortes-Garcia N, Linares-Abad M (2018). Myasthenia gravis and pregnancy. Matronas Profesion..

[90] Pacu I, Sardescu G, Pacu O, Tarcomnicu I, Ionescu CA (2013). Thyroid antibodies and risk of preterm delivery: a meta-analysis of prospective cohort studies. Arch Balkan Med Union.

[91] Park JJ, Kim HJ, Kim MA (2016). The influence of disease activity on birth outcomes in patients with inflammatory bowel disease: meta-analysis. J Crohn’s Colitis.

[92] Petri M (1998). Pregnancy in SLE. Bailliere’s Clin Rheumatol.

[93] Piccioni MG, Tabacco S, Giannini A, Deroma M, Logoteta A, Monti M (2020). Myasthaenia gravis in pregnancy, delivery and newborn. Minerva Ginecol.

[94] Piccoli GB, Clari R, Ghiotto S, Castelluccia N, Colombi N, Mauro G (2013). Type 1 diabetes, diabetic nephropathy, and pregnancy: a systematic review and meta-study. Rev Diabet Stud.

[95] Prummel MF, Wiersinga WM (2004). Thyroid autoimmunity and miscarriage. Eur J Endocrinol.

[96] Schmidt PMS, Longoni A, Pinheiro RT, Assis AM (2022). Postpartum depression in maternal thyroidal changes. Thyroid Res.

[97] Sim BL, Daniel RS, Hong SS, Matar RH, Ganiel I, Nakanishi H (2023). Pregnancy outcomes in women with rheumatoid arthritis: a systematic review and meta-analysis. J Clin Rheumat..

[98] Smyth A, Oliveira GH, Lahr BD, Bailey KR, Norby SM, Garovic VD (2010). A systematic review and meta-analysis of pregnancy outcomes in patients with systemic lupus erythematosus and lupus nephritis. Clin J Am Soc Nephrol.

[99] Tian L, Zhang Z, Mao Y, Zong M (2023). Association between pregnant women with rheumatoid arthritis and preeclampsia: a systematic review and meta-analysis. Medicine (Baltimore).

[100] Toulis KA, Goulis DG, Venetis CA, Kolibianakis EM, Negro R, Tarlatzis BC (2010). Risk of spontaneous miscarriage in euthyroid women with thyroid autoimmunity undergoing IVF: a meta-analysis. Eur J Endocrinol.

[101] Wang P, Wang Z, He X, Xu D, Wang B (2012). Thyroid antibodies and risk of preterm delivery: a meta-analysis of prospective cohort studies. Eur J Endocrinol.

[102] Wu H, Hong T, Gao H, Wang H (2015). Effects of thyroid autoimmunity on pregnancy outcomes in euthyroid women receiving in vitro fertilization: a meta-analysis. Chung-Hua i Hsueh Tsa Chih [Chinese Medical Journal].

[103] Yang Y, Li Q, Wang Q, Ma X (2015). Thyroid antibodies and gestational diabetes mellitus: a meta-analysis. Fertil Steril.

[104] Shea BJ, Reeves BC, Wells G, Thuku M, Hamel C, Moran J (2017). AMSTAR 2: a critical appraisal tool for systematic reviews that include randomised or non-randomised studies of healthcare interventions, or both. BMJ.

[105] Hennessy EA, Johnson BT (2020). Examining overlap of included studies in meta-reviews: guidance for using the corrected covered area index. Res Synth Methods.

[106] Lunny C, Pieper D, Thabet P, Kanji S. Managing overlap of primary study results across systematic reviews: practical considerations for authors of overviews of reviews. BMC Med Res Methodol. 2021;21(1):140. 10.1186/s12874-021-01269-y PMC8265144 34233615

[107] Pollock M, Fernandes RM, Newton AS, Scott SD, Hartling L (2019). A decision tool to help researchers make decisions about including systematic reviews in overviews of reviews of healthcare interventions. Syst Rev.

[108] Fusar-Poli P, Radua J (2018). Ten simple rules for conducting umbrella reviews. Evidence Based Mental Health.

[109] Arvanitakis K, Siargkas A, Germanidis G, Dagklis T, Tsakiridis I (2023). Adverse pregnancy outcomes in women with celiac disease: a systematic review and meta-analysis. Ann Gastroenterol..

[110] Bundhun PK, Soogund MZ, Huang F (2017). Impact of systemic lupus erythematosus on maternal and fetal outcomes following pregnancy: a meta-analysis of studies published between years 2001–2016. J Autoimmun.

[111] Chen L, Hu R (2011). Thyroid autoimmunity and miscarriage: a meta-analysis. Clin Endocrinol.

[112] Cornish J, Tan E, Teare J, Teoh TG, Rai R, Clark SK (2007). A meta-analysis on the influence of inflammatory bowel disease on pregnancy. Gut.

[113] Dong AC, Morgan J, Kane M, Stagnaro-Green A, Stephenson MD (2020). Subclinical hypothyroidism and thyroid autoimmunity in recurrent pregnancy loss: a systematic review and meta-analysis. Fertil Steril.

[114] Geng B, Zhang K, Huang X, Chen Y (2022). A meta-analysis of the effect of Sjögren’ s syndrome on adverse pregnancy outcomes. Clinics.

[115] He X, Wang P, Wang Z, He X, Xu D, Wang B (2012). Thyroid antibodies and risk of preterm delivery: a meta-analysis of prospective cohort studies. Eur J Endocrinol.

[116] Korevaar TIM, Derakhshan A, Taylor PN, Meima M, Chen L, Bliddal S (2020). Association of thyroid function test abnormalities and thyroid autoimmunity with preterm birth: a systematic review and meta-analysis. Obstet Gynecol Surv.

[117] Leung KK, Tandon P, Govardhanam V, Maxwell C, Huang V (2021). The risk of adverse neonatal outcomes with maternal inflammatory bowel disease: a systematic review and meta-analysis. Inflamm Bowel Dis.

[118] Li M, Wang SW, Wu FL, Shi J, Yu PL, Peng XL (2016). Ethnic differences in preterm birth risks for pregnant women with thyroid dysfunction or autoimmunity: a meta-analysis. Biomed Environ Sci.

[119] Luo J, Wang X, Yuan L, Guo L (2021). Association of thyroid disorders with gestational diabetes mellitus: a meta-analysis. Endocrine.

[120] Minaldi E, D’Andrea S, Castellini C, Martorella A, Francavilla F, Francavilla S (2020). Thyroid autoimmunity and risk of post-partum depression: a systematic review and meta-analysis of longitudinal studies. J Endocrinol Invest.

[121] Modrego PJ, Urrea MA, De Cerio LD (2021). The effects of pregnancy on relapse rates, disability and peripartum outcomes in women with multiple sclerosis: a systematic review and meta-Analysis. J Comp Eff Res.

[122] Saccone G, Berghella V, Sarno L, Maruotti GM, Cetin I, Greco L (2016). Celiac disease and obstetric complications: a systematic review and metaanalysis. Am J Obstet Gynecol.

[123] Talavera JI, Parrill AM, Elsayad C, Fogel J, Riggs JC, Peng B (2021). The association between ectopic pregnancy and inflammatory bowel disease, irritable bowel syndrome, and celiac disease: a systematic review. J Obstet Gynaecol Res.

[124] Tersigni C, Castellani R, de Waure C, Fattorossi A, De Spirito M, Gasbarrini A (2014). Celiac disease and reproductive disorders: meta-analysis of epidemiologic associations and potential pathogenic mechanisms. Hum Reprod Update.

[125] Thangaratinam S, Tan A, Knox E, Kilby MD, Franklyn J, Coomarasamy A (2011). Association between thyroid autoantibodies and miscarriage and preterm birth: meta-analysis of evidence. Bmj..

[126] Tong Z, Xiaowen Z, Baomin C, Aihua L, Yingying Z, Weiping T (2016). The effect of subclinical maternal thyroid dysfunction and autoimmunity on intrauterine growth restriction: a systematic review and meta-analysis. Medicine.

[127] Upala S, Yong WC, Sanguankeo A. Association between primary Sjögren’s syndrome and pregnancy complications: a systematic review and meta-analysis. Clin Rheumatol. 2016;35(8):1949–55. 10.1007/s10067-016-3323-9 27271701

[128] Xie W, Huang H, Ji L, Zhang Z (2021). Maternal and neonatal outcomes in pregnant women with psoriasis and psoriatic arthritis: a systematic review and meta-analysis. Rheumatology.

[129] Yu L, Zeng XL, Cheng ML, Yang GZ, Wang B, Xiao ZW (2017). Quantitative assessment of the effect of pre-gestational diabetes and risk of adverse maternal, perinatal and neonatal outcomes. Oncotarget.

[130] Zhang SC, Wang SW, Zhao XD, Zhang JR (2016). Obstetrical complications of thyroid peroxidase antibody positive during pregnancy and effects of intervention: a meta-analysis. Zhonghua Fu Chan Ke Za Zhi..

[131] Lv J, Xu L, Mao S (2023). Association between disease activity of rheumatoid arthritis and maternal and fetal outcomes in pregnant women: a systematic review and meta-analysis. BMC Pregnancy Childbirth.

[132] Wells GA, Shea B, O’Connell D, Peterson J, Welch V, Losos M, et al. The Newcastle-Ottawa Scale (NOS) for assessing the quality of nonrandomised studies in meta-analyses. Oxford; 2000.

[133] Ahmadzai N, Newberry SJ, Maglione MA, Tsertsvadze A, Ansari MT, Hempel S (2013). A surveillance system to assess the need for updating systematic reviews. Syst Rev.

[134] Begg CB, Mazumdar M (1994). Operating characteristics of a rank correlation test for publication bias. Biometrics..

[135] Dalton JE, Bolen SD, Mascha EJ (2016). Publication bias: the elephant in the review. Anesth Analg.

[136] Egger M, Smith GD, Schneider M, Minder C (1997). Bias in meta-analysis detected by a simple, graphical test. BMJ.

[137] Garner P, Hopewell S, Chandler J, MacLehose H, Akl EA, Beyene J (2016). When and how to update systematic reviews: consensus and checklist. BMJ.

[138] Hennessy EA, Johnson BT (2020). Examining overlap of included studies in meta-reviews: guidance for using the corrected covered area index. Res Synth Methods.

[139] Page MJ, McKenzie JE, Bossuyt PM, Boutron I, Hoffmann TC, Mulrow CD (2021). The PRISMA 2020 statement: an updated guideline for reporting systematic reviews. BMJ.

[140] Smith GD, Ho KH. Systematic reviews: When should they be updated? : Wiley Online Library; 2023. p. e17–e8. 10.1111/jocn.16547 36153703

[141] van den Boogaard E, Vissenberg R, Land JA, van Wely M, van der Post JAM, Goddijn M (2011). Significance of (sub)clinical thyroid dysfunction and thyroid autoimmunity before conception and in early pregnancy: a systematic review. Hum Reprod Update.

[142] Wang L, Wang F-S, Gershwin ME (2015). Human autoimmune diseases: a comprehensive update. J Intern Med.

[143] Horev A, Weintraub AY, Sergienko R, Wiznitzer A, Halevy S, Sheiner E (2011). Pregnancy outcome in women with vitiligo. Int J Dermatol.

[144] Kolstad KD, Fiorentino D, Li S, Chakravarty EF, Chung L (2018). Pregnancy outcomes in adult patients with dermatomyositis and polymyositis. Semin Arthritis Rheum.

[145] Deguchi M, Yamada H, Sugiura-Ogasawara M, Morikawa M, Fujita D, Miki A (2017). Factors associated with adverse pregnancy outcomes in women with antiphospholipid syndrome: a multicenter study. J Reprod Immunol.

[146] Alarcón-Segovia D, Delezé M, Oria CV, Sánchez-Guerrero J, Gómez-Pacheco L, Cabiedes J (1989). Antiphospholipid antibodies and the antiphospholipid syndrome in systemic lupus erythematosus a prospective analysis of 500 consecutive patients. Medicine..

[147] Hung C-T, Huang H-H, Wang C-K, Chung C-H, Tsao C-H, Chien W-C (2021). Pregnancy outcomes in women with vitiligo: a Taiwanese nationwide cohort study. PLoS One.

[148] Park KY, Kwon HJ, Wie JH, Lee HH, Cho SB, Kim BJ (2018). Pregnancy outcomes in patients with vitiligo: a nationwide population-based cohort study from Korea. J Am Acad Dermatol.

[149] Schneiderman M, Czuzoj-Shulman N, Spence A, Abenhaim H (2017). Maternal and neonatal outcomes of pregnancies in women with Addison’s disease: a population-based cohort study on 7.7 million births. BJOG..

[150] Bjornsdottir S, Cnattingius S, Brandt L, Nordenstrom A, Ekbom A, Kampe O (2010). Addison’s disease in women is a risk factor for an adverse pregnancy outcome. J Clin Endocrinol Metab.

[151] Cho SI, Yu D-A, Kim SI, Lee SM, Kwon O (2021). Pregnancy outcomes in female patients with alopecia areata: a nationwide population-based study. J Investig Dermatol.

[152] Chen JS, Roberts CL, Simpson JM, March LM (2015). Pregnancy outcomes in women with rare autoimmune diseases. Arthritis & Rheumatology.

[153] Spinillo A, Beneventi F, Epis OM, Montanari L, Mammoliti D, Ramoni V (2008). The effect of newly diagnosed undifferentiated connective tissue disease on pregnancy outcome. Am J Obstet Gynecol..

[154] Spinillo A, Beneventi F, Caporali R, Ramoni V, Montecucco C (2017). Undifferentiated connective tissue diseases and adverse pregnancy outcomes. An undervalued association?. Am J Reprod Immunol.

[155] Mosca M, Neri R, Strigini F, Carmignani A, Totti D, Tavoni A (2002). Pregnancy outcome in patients with undifferentiated connective tissue disease: a preliminary study on 25 pregnancies. Lupus.

[156] Sperber K, Hom C, Chao CP, Shapiro D, Ash J (2009). Systematic review of hydroxychloroquine use in pregnant patients with autoimmune diseases. Pediatr Rheumatol.

[157] Mecacci F, Pieralli A, Bianchi B, Paidas MJ, editors. The impact of autoimmune disorders and adverse pregnancy outcome. Sem Perinatol. 2007;31(4):223–6. 10.1053/j.semperi.2007.05.005 17825677

[158] Tsao NW, Lynd LD, Sadatsafavi M, Hanley G, De Vera MA (2018). Patterns of biologics utilization and discontinuation before and during pregnancy in women with autoimmune diseases: a population-based cohort study. Arthritis Care Res.

[159] Götestam Skorpen C, Lydersen S, Gilboe IM, Skomsvoll JF, Salvesen K, Palm Ø (2018). Women with systemic lupus erythematosus get pregnant more easily than women with rheumatoid arthritis. Rheumatology (Oxford).

[160] Suciu N, Pop L, Panaitescu E, Suciu ID, Popp A, Anca I (2014). Fetal and neonatal outcome in celiac disease. J Matern Fetal Neonatal Med.

[161] Lopez-Leon S, Geissbuehler Y, Sabido M, Turkson M, Wahlich C, Morris J (2020). A systematic review and meta-analyses of pregnancy and fetal outcomes in women with multiple sclerosis. IMI2 conception. Mult Scler J.

[162] Ross G, Sammaritano L, Nass R, Lockshin M (2003). Effects of mothers’ autoimmune disease during pregnancy on learning disabilities and hand preference in their children. Arch Pediatr Adolesc Med.

[163] Nielsen TC, Nassar N, Shand AW, Jones H, Guastella AJ, Dale RC (2021). Association of maternal autoimmune disease with attention-deficit/hyperactivity disorder in children. JAMA pediatrics..

[164] Chen S-w, Zhong X-s, Jiang L-n, Zheng X-y, Xiong Y-q, Ma S-j (2016). Maternal autoimmune diseases and the risk of autism spectrum disorders in offspring: a systematic review and meta-analysis. Behav Brain Res.

[165] Norwood F, Dhanjal M, Hill M, James N, Jungbluth H, Kyle P (2014). Myasthenia in pregnancy: best practice guidelines from a U.K. multispecialty working group. J Neurol Neurosurg Psychiatry..

[166] Murphy HR (2021). 2020 NICE guideline update: good news for pregnant women with type 1 diabetes and past or current gestational diabetes. Diabet Med.

[167] Dobson R, Dassan P, Roberts M, Giovannoni G, Nelson-Piercy C, Brex PA (2019). UK consensus on pregnancy in multiple sclerosis: ‘Association of British Neurologists’ guidelines. Pract Neurol.

[168] Russell MD, Dey M, Flint J, Davie P, Allen A, Crossley A (2022). Executive Summary: British Society for Rheumatology guideline on prescribing drugs in pregnancy and breastfeeding: immunomodulatory anti-rheumatic drugs and corticosteroids. Rheumatology.

[169] Sammaritano LR, Bermas BL, Chakravarty EE, Chambers C, Clowse MEB, Lockshin MD (2020). 2020 American College of Rheumatology Guideline for the Management of Reproductive Health in Rheumatic and Musculoskeletal Diseases. Arthritis Care Res (Hoboken).

[170] Skorpen CG, Hoeltzenbein M, Tincani A, Fischer-Betz R, Elefant E, Chambers C (2016). The EULAR points to consider for use of antirheumatic drugs before pregnancy, and during pregnancy and lactation. Ann Rheum Dis.

[171] Gordon C (2004). Pregnancy and autoimmune diseases. Best Pract Res Clin Rheumatol.

[172] Keestra SM, Male V, Salali GD (2021). Out of balance: the role of evolutionary mismatches in the sex disparity in autoimmune disease. Med Hypotheses.

[173] Furer V, Rondaan C, Heijstek MW, Agmon-Levin N, Van Assen S, Bijl M (2020). 2019 update of EULAR recommendations for vaccination in adult patients with autoimmune inflammatory rheumatic diseases. Ann Rheum Dis.

[174] Nana M, Stannard MT, Nelson-Piercy C, Williamson C (2023). The impact of preconception counselling on maternal and fetal outcomes in women with chronic medical conditions: a systematic review. Eur J Intern Med.

[175] Andreoli L, Bertsias GK, Agmon-Levin N, Brown S, Cervera R, Costedoat-Chalumeau N (2017). EULAR recommendations for women’s health and the management of family planning, assisted reproduction, pregnancy and menopause in patients with systemic lupus erythematosus and/or antiphospholipid syndrome. Ann Rheum Dis.

[176] de Lima A, Zelinkova Z, Mulders AG, van der Woude CJ (2016). Preconception care reduces relapse of inflammatory bowel disease during pregnancy. Clin Gastroenterol Hepatol.

[177] Wotherspoon AC, Young IS, McCance DR, Holmes VA (2016). Evaluation of biomarkers for the prediction of pre-eclampsia in women with type 1 diabetes mellitus: a systematic review. J Diabetes Complications.

