# Supplementary material for: Autoimmune diseases and adverse pregnancy outcomes: an umbrella review
Source: BMC Med. 2024 Mar 5;22:94. doi: 10.1186/s12916-024-03309-y (PMC10913233; doi:10.1186/s12916-024-03309-y)
Supplement: Supplementary file 1 — Additional file 1: Text S1. Details of the overlappling reviews, quality assessment and heterogeneity. Table S1. PRIOR checklist (Preferred Reporting Items for Overviews of Reviews. Table S2. Deviations from protocol. Table S3. Search Strategy OVID MEDLINE. Table S4. List of excluded studies. Table S5. Data extraction form. Table S6. Quality Assessment of included studies using AMSTAR 2 Tool. Table S7.1-7.9. Citation matrices for reviews with overlapping association. Table S8. Overlapping and non-overlapping association. Table S9. Forest plots of the combined review. Table S10. Evaluation of need to update the reviews. Table S11. General characteristics of systematic reviews included in the umbrella review. Table S12. Tabular presentation of findings: Meta-analysis. Table S13. Tabular presentation of findings: Narrative synthesis. [file 12916_2024_3309_MOESM1_ESM.docx]

[Additional file text 1 6](#_Toc132224503)

[Additional file table 1 PRIOR checklist(Preferred Reporting Items for Overviews of Reviews…………… 3 Additional file table 2 Deviations from protocol 8](#_Toc132224504)

[Additional file table 3 Search Strategy OVID MEDLINE 11](#_Toc132224505)

[Additional file table 4 List of excluded studies 14](#_Toc132224506)

[Additional file table 5 Data extraction form 21](#_Toc132224507)

Additional file [table 6 Quality Assessment of included studies using AMSTAR 2 Tool 22](#_Toc132224508)

[Additional file table 7 Citation matrices for reviews with overlapping associations 24](#_Toc132224509)

Additional file table 7.1Axial spondyloarthropathy………………………………………….………………………..……23

Additional file table 7.2 Coeliac diseases…………………………………….………………………………….…....………...23

Additional file table 7.3 Inflammatory bowel disease…………………………………………………….………..…..…25

Additional file table 7.4 Psoriasis………………………………………………………………………………………….….………28

Additional file table 7.5 Psoriatic arthritis………………………………………………………………………………..….…..29

Additional file table 7.6 Rheumatoid arthritis………………………………………………………..………………….….…30

Additional file table 7.7Sjogren syndrome………………………………………………………………………………...…….32

Additional file table 7.8-Systemic lupus erythematosus………………………………………..…………….…..………33

Additional file table 7.7-Thyroid autoimmunity (all)………………………………………………………….……..…….34

Additional file table 7.8- Thyroid autoimmunity (TPO)………………………………………………………..………....38

Additional file table 7.9 Ulcerative colitis and Crohn’s disease…………………………………….………………….39

Additional file table 8-overlapping and non-overlapping association………………………………..……………41

Additional file table 9-Forest plots of the combined review ……………………………………….…………………45

Additional file table 10- Evaluation of need to update the reviews …………………………………………….48

Additional file table 11 General characteristics of systematic reviews included in the umbrella review……………………………………………………………………………………………………………………………………….….40

Additional file table 12: Tabular presentation of findings: Meta-analysis ……………………………..…….65

Additional file table 13.Tabular presentation of findings: Narrative synthesis ………………………..……72

Additional file text 1

Quality assessment

Of the 46 initially included reviews, four reviews were subsequently excluded due to being critically low in quality. They were quality assessed using the AMSTAR2 tool. The critical domains which are important for a review to be high quality review are Protocol registered before commencement of the review (item 2), Adequacy of the literature search (item 4), Justification for excluding individual studies (item 7), Risk of bias from individual studies being included in the review (item 9), Appropriateness of meta-analytical methods (item 11), Consideration of risk of bias when interpreting the results of the review (item 13).Assessment of presence and likely impact of publication bias (item 15). The four reviews which were excluded did not qualify on more than 3 items of the critical domain listed.

Out of the remaining 42 studies, Out of the remaining 42 reviews, 20 were rated as of moderate quality and remaining were low in quality. None of the reviews were rated high quality. The critical domains that were not met in the reviews were, firstly almost 23% of these reviews did not take in account risk of bias while interpreting the results, while almost 26% of the studies did not report the disclosure of the funding or conflict of interest. Further almost 15 % of the studies did not register protocol before commencing the review or reported the justification of the studies which were excluded. Also 5-6% of the studies did not include risk of bias assessment of the included studies .In most of the included systematic reviews, the quality of the primary studies was assessed through Newcastle-Ottawa scale (1). And most of the reviews study quality varied moderate to high quality of studies.

**Reviews with overlapping primary studies and update of reviews**

The overlapping association was noted with most autoimmune conditions reporting various pregnancy outcomes except for autoimmune conditions, myasthenia gravis, multiple sclerosis, and systemic sclerosis, as only one review for each of these conditions was identified. CCA less than 5% indicates low overlap and CCA 10 or above indicates moderate/high overlap. Where reviews had a high overlap (CCA ≥10%), Cochrane review was selected over a non-Cochrane review. If there were no Cochrane reviews, then a review was selected if: it had higher AMSTAR quality rating, reported more adverse pregnancy outcomes, was conducted recently, included a meta-analysis, or had a larger sample size (2, 3). In cases where the reviews had non-overlapping association (CCA<10%), the reviews were retained and the data from primary studies of the reviews were extracted and without double counting of the primary studies a meta-analysis was conducted to generate pooled effect sizes. Overalapping association between many reviews (4-27).The degree of overlap (CCA) ranged from 0% to 66%. Where the overlap was high with CCA ≥10% one review was retained and the others were excluded based on the criteria for exclusion or inclusion as reported earlier. Nine reviews were excluded owing to high overlap (6, 12-14, 18, 21, 25-27)

Highly overlapping reviews (CCA > 10%) High overlap was noted with two reviews reporting association of axial spondyloarthropathy and small for gestational age (n=6), preterm birth (n=13) ,pre-eclampsia (n=7) and caesarean section (n=11) (9, 14) (n=number of primary studies included).Both were reviews from same year and moderate quality, hence review with larger sample size was selected. Reviews reporting association of coeliac disease and preterm birth (n=7), Intra uterine growth retardation (n=11), stillbirth (n=5) ,low birth weight (n=6) had high overlap (20, 23). High overlap was also noted in the reviews reporting Inflammatory bowel disease and stillbirth (n=11) (11, 19), preterm birth (n=31), small for gestational (n=18) (11, 15, 19). Reviews reporting psoriasis and miscarriage (n=8), caesarean section (n=11) were also overlapping with high CCA. Since one of the review presented the result in narrative analysis the review with meta-analysis was preferred to report the results (12, 24). Reviews reporting psoriatic arthritis and pre-eclampsia (n=10), caesarean section (n=10), preterm birth(n=10), small for gestational age(n=8) (14, 24). Reviews reporting gestational diabetes in women with systemic lupus erythematosus (n=6) also reported high overlap (4, 6).The newer review though included less number of studies (n=13,883) but large sample size was selected to report the results (4).Thyroid autoimmunity (all) and preterm birth (n=23). recurrent miscarriage (n=19) or miscarriage (n=27), gestational diabetes (n=30) and postpartum depression (n=19) in presence of thyroid autoimmunity had high overlap (16, 25, 26, 28-32).Thyroid autoimmunity (TPO) and preterm birth (n=29) was reported by many reviews and high overlap was seen.(18, 26, 31, 33-35). Furthermore while evaluating the association of Crohns disease and ulcerative colitis with preterm birth high overlap was observed (11, 19).

Moderate to low overlapping reviews (CCA<10%)-Two reviews reporting association of coeliac disease and small for gestational age and pre-eclampsia had low CCA suggesting nonoverlapping association with CCA 0% because of the difference in inclusion criteria one of the reviews included cohort studies and other included case control studies.(20, 23).Two reviews reporting IBD and caesarean section had low CCA with one overlapping study. as one review was from 2007 and included older studies and another new review did not take in account the older studies. There was overlap of few studies which were counted once when meta-analysing the primary studies. Two reviews reporting IBD and caesarean section had low CCA with one overlapping study. as one review was from 2007 and included older studies and another new review did not take in account the older studies. There was overlap of few studies which were counted once when meta-analysing the primary studies. Two reviews reporting psoriasis and preterm birth and low birth weight reported weak overlap and hence results from both are presented ,one of the reviews reports summary estimates and another narrative analysis (12, 24).

.

CCA of less than 10% was observed in reviews estimating the association of small for gestational age and AxSpa or SLE, and preterm birth and SLE or rheumatoid arthritis, Caesarean section and IBD.

Three reviews reporting preterm birth in women with SLE had nonoverlapping associations(4, 7, 8). The nonoverlap was because the the reviews searched strategy was restricted to certain year and they used different databases to run the searches Due to missing data the odds ratios from the primary studies were used to menta-analyse and not the primary data. 2 primary studies (Bernardo 2014 and Yuen ) were overlapping they were counted once and all primary studies were meta-analysed to present the summary effect size appendix fig 13. Similarly 2 reviews reporting low birth weight, caesarean section and small for gestational age with SLE as exposure were non-overlapping(4, 7).The primary studies from these reviews were also meta-analysed to get a summary effect appendix.The non-overlapping association was also noted in revies reporting thyroid autoimmunity(TgAb) and preterm birth and gestational diabetes(16, 25, 31, 33). In case of all the non overlapping reviews the data from primary studies were extracted and meta-analysed without double counting and summary estimates were presented.

Data synthesis- Various pregnancy complications have been reported for each autoimmune condition and after carefully examining the overlap only the nonoverlapping results have been presented. After quantification of overlap between the review in form of CCA

1. CCA ≥10%=Two reviews with high overlap (CCA > 10%). One review was selected to present the results based on the criteria mentioned earlier.
2. CCA<10%=Few overlapping primary studies between the reviews. Data from the primary studies of the overlapping reviews was extracted and a meta-analysis (random effect) was performed without double counting the overlapping primary studies included in the reviews.
3. CCA=0%=No overlapping primary studies between the reviews. Data from the primary studies of these reviews was extracted and a meta-analysis (random effect) was performed to get the summary estimate.
4. The details of the overlapping association are presented in Table 7, 8 and 9. Leung et al present their results separately for cohort studies and registries for a few of the outcomes (low birth weight, preterm birth and small for gestational age), which were combined to present a summary estimate.
5. A further two reviews (Leung et al and Cornish et al) had non-overlapping associations reporting inflammatory bowel disease and low birth weight. As there was some missing data (no information of number of participants in the exposed population group or the control group in the manuscript or supplementary material) odds ratio from the studies were combined.

The results were presented in the form of odds ratios in the forest plots. In the reviews where summary estimates were reported as either a risk ratio or hazards ratio, we used an appropriate method of conversion to calculate the odds ratio (36).

When presenting association of thyroid autoimmunity,thyroid autoimmunity(all) refers to presence of both thyroglobulin antibodies(TgAb) and thyroid peroxidase antibodies(TPO), where as thyroid autoimmunity(TPO) suggests only presence of thyroid autoantibodies has been studies.

When presenting the association of thyroid autoimmunity, ‘thyroid autoimmunity (all)’ refers to the presence of both thyroglobulin antibodies (TgAb) and thyroid peroxidase antibodies (TPO). Whereas ‘thyroid autoimmunity (TPO)’ indicates only the presence of thyroid peroxidase autoantibodies has been studied. The details of the overlapping association are presented in Table 7, 8 and 9. Leung et al present their results separately for cohort studies and registries for few outcomes (low birth weight, preterm birth and small for gestational age) which were combined to present a summary estimate. Further two reviews (leung et al and Cornish et al) had non-overlapping association reporting low birth weight in Inflammatory bowel disease, due to missing data odds ration from the studies are combined.

**Update of reviews**

The reviews were carefully examined following guidelines from Garner et al to identify whether the reviews needed to be updated (9). A systematic review with meta-analysis was considered out of date if a newly published study led to a change of the result by at least 50%. To evaluate the need to update the reviews, the RAND and OTTAWA methods were used (37, 38). Details of the methods are presented in Table 10. Most of the reviews included were published in the last 10 years (2014-2021). To identify the need and possibility of updating the older included reviews, a scoping search was conducted to identify new primary studies for the given exposures and outcome. A few new studies were identified for some exposures such as IBD or coeliac disease. However, none of the study results were contradictory to findings of existing included reviews or would change the conclusion by 50% or more if included in the umbrella review. After consultation with experts, it was concluded that the reviews at this stage are not required to be updated.

**Heterogeneity**

All the included meta-analysis reported statistical heterogeneity between the included studies. Most calculated the heterogeneity among the studies using I^2^ and values ranged from 0-98%. Most of the reviews have taken into account the level of heterogeneity while reporting the results. Three meta-analysis reported Q test with P values ranging from 0.0001-0.03 (33, 39, 40). The meta-analysis performed in this umbrella review to combine the non-overlapping reviews reported I^2^ statistics ranging from 0-98%. Details in table 6.

**Publication bias**

Most of the included meta-analyses included funnel plots, Egger’s linear regression, and Begg’s rank correlation test to evaluate potential publication bias qualitatively or quantitatively (41, 42). The studies reported variable publication bias, but seven meta-analysis did not report publication bias (10, 15, 22, 28, 34, 43, 44). There were 13 meta-analysis conducted in this review, out of which 6 meta-analysis included less than 10 studies and so publication bias could not be assessed (45). For the remaining 6 meta-analysis the potential publication bias was assessed using Egger’s test via funnel plot asymmetry and has a low risk of publication bias (table 9 along with the forest plots).

# Table 1 PRIOR checklist(Preferred Reporting Items for Overviews of Reviews)

| Section |  | |  |  |
| --- | --- | --- | --- | --- |
| topic | Item No | | Item | Location where item is reported |
| **Title** |  | |  |  |
| Title | 1 | | Identify the report as an overview of reviews. | Line 1 |
| **Abstract** |  | |  |  |
| Abstract | 2 | | Provide a comprehensive and accurate summary of the purpose, methods, and results of the overview of reviews. | Line 16 to 45 |
| **Introduction** |  | |  |  |
| Rationale | 3 | | Describe the rationale for conducting the overview of reviews in the context of existing knowledge. | Line 46  background |
| Objectives | 4 | | Provide an explicit statement of the objective(s) or question(s) addressed by the overview of reviews. | Line 83-93 |
| Methods |  | |  |  |
| Eligibility criteria | 5a | | Specify the inclusion and exclusion criteria for the overview of reviews. If supplemental primary studies were included, this should be stated, with a rationale. | Inclusion and exclusion criteria  Line 103-133 |
|  | 5b | | Specify the definition of “systematic review” as used in the inclusion criteria for the overview of reviews. | Line 124-126 |
| Information sources | 6 | | Specify all databases, registers, websites, organisations, reference lists, and other sources searched or consulted to identify systematic reviews and supplemental primary studies (if included). Specify the date when each source was last searched or consulted. | Search strategy  Line 134-141 |
| Search strategy | 7 | | Present the full search strategies for all databases, registers and websites, such that they could be reproduced. Describe any search filters and limits applied. | Search strategy  Line 134-141  Additional file table 3 |
| Selection process | 8a | | Describe the methods used to decide whether a systematic review or supplemental primary study (if included) met the inclusion criteria of the overview of reviews. | Study selection  Line 142-153 |
|  | 8b | | Describe how overlap in the populations, interventions, comparators, and/or outcomes of systematic reviews was identified and managed during study selection. | Line 178-186 and  Additional file text 1 |
| Data collection process | 9a | | Describe the methods used to collect data from reports. | Data extraction  Line 154-164 |
|  | 9b | | If applicable, describe the methods used to identify and manage primary study overlap at the level of the comparison and outcome during data collection. For each outcome, specify the method used to illustrate and/or quantify the degree of primary study overlap across systematic reviews. | Line 178-186  Additional file text 1, table 7 and 8 |
|  | 9c | | If applicable, specify the methods used to manage discrepant data across systematic reviews during data collection. | Data extraction  Line 154-164 |
| Data items | 10 | | List and define all variables and outcomes for which data were sought. Describe any assumptions made and/or measures taken to identify and clarify missing or unclear information. | Data extraction  Line 154-164  And Additional file table 5 |
| Risk of bias assessment | 11a | | Describe the methods used to assess risk of bias or methodological quality of the included systematic reviews. | Quality assessment  Line 167 |
|  | 11b | | Describe the methods used to collect data on (from the systematic reviews) and/or assess the risk of bias of the primary studies included in the systematic reviews. Provide a justification for instances where flawed, incomplete, or missing assessments are identified but not reassessed. | Quality assessment  Line 165-177 |
|  | 11c | | Describe the methods used to assess the risk of bias of supplemental primary studies (if included). | NA |
| Synthesis methods | 12a | | Describe the methods used to summarise or synthesise results and provide a rationale for the choice(s). | Data synthesis  Line 206-224 |
|  | 12b | | Describe any methods used to explore possible causes of heterogeneity among results. | Data synthesis  Line 206-224 |
|  | 12c | | Describe any sensitivity analyses conducted to assess the robustness of the synthesised results. | NA |
| Reporting bias assessment | 13 | | Describe the methods used to collect data on (from the systematic reviews) and/or assess the risk of bias due to missing results in a summary or synthesis (arising from reporting biases at the levels of the systematic reviews, primary studies, and supplemental primary studies, if included). | Data synthesis  Line 187-202 |
| Certainty assessment | 14 | | Describe the methods used to collect data on (from the systematic reviews) and/or assess certainty (or confidence) in the body of evidence for an outcome. | Data synthesis  Line 206-224 |
| Results |  |  | |  |
| Systematic review and supplemental primary study selection | 15a | Describe the results of the search and selection process, including the number of records screened, assessed for eligibility, and included in the overview of reviews, ideally with a flow diagram. | | Results  Line 209 onwards and  PRISMA flowchart |
|  | 15b | Provide a list of studies that might appear to meet the inclusion criteria, but were excluded, with the main reason for exclusion. | | Additional file table 4 |
| Characteristics of systematic reviews and supplemental primary studies | 16 | Cite each included systematic review and supplemental primary study (if included) and present its characteristics. | | Table 2 main document and Additional file table 11,12 ,13 |
| Primary study overlap | 17 | Describe the extent of primary study overlap across the included systematic reviews. | | Line 178-186  Additional file text 1, table 7 and 8 |
| Risk of bias in systematic reviews, primary studies, and supplemental primary studies | 18a | Present assessments of risk of bias or methodological quality for each included systematic review. | | Quality assessment  Line 165-177 Additional file table 6 |
|  | 18b | Present assessments (collected from systematic reviews or assessed anew) of the risk of bias of the primary studies included in the systematic reviews. | | Additional file table 11 |
|  | 18c | Present assessments of the risk of bias of supplemental primary studies (if included). | | NA |
| Summary or synthesis of results | 19a | For all outcomes, summarise the evidence from the systematic reviews and supplemental primary studies (if included). If meta-analyses were done, present for each the summary estimate and its precision and measures of statistical heterogeneity. If comparing groups, describe the direction of the effect. | | Summary of the results  Line 282 onwards  Figures 2 and 3 and table 3 |
|  | 19b | If meta-analyses were done, present results of all investigations of possible causes of heterogeneity. | | Additional file text 1 |
|  | 19c | If meta-analyses were done, present results of all sensitivity analyses conducted to assess the robustness of synthesised results. | | NA |
| Reporting biases | 20 | Present assessments (collected from systematic reviews and/or assessed anew) of the risk of bias due to missing primary studies, analyses, or results in a summary or synthesis (arising from reporting biases at the levels of the systematic reviews, primary studies, and supplemental primary studies, if included) for each summary or synthesis assessed. | | Line 466-474  Additional file text 1 |
| Certainty of evidence | 21 | Present assessments (collected or assessed anew) of certainty (or confidence) in the body of evidence for each outcome. | | Strengths and limitations  Line 449 |
| **Discussion** |  |  | |  |
| Discussion | 22a | Summarise the main findings, including any discrepancies in findings across the included systematic reviews and supplemental primary studies (if included). | | Line 448-458  Table 3 |
|  | 22b | Provide a general interpretation of the results in the context of other evidence. | | Conclusion  Line 514-417 |
|  | 22c | Discuss any limitations of the evidence from systematic reviews, their primary studies, and supplemental primary studies (if included) included in the overview of reviews. Discuss any limitations of the overview of reviews methods used. | | Strength and limitation  Line 449 |
|  | 22d | Discuss implications for practice, policy, and future research (both systematic reviews and primary research). Consider the relevance of the findings to the end users of the overview of reviews, eg, healthcare providers, policymakers, patients, among others. | | Line 497-513 |
| **Other information** |  |  | |  |
| Registration and protocol | 23a | Provide registration information for the overview of reviews, including register name and registration number, or state that the overview of reviews was not registered. | | Methods line 101 |
|  | 23b | Indicate where the overview of reviews protocol can be accessed, or state that a protocol was not prepared. | | Methods line 101 |
|  | 23c | Describe and explain any amendments to information provided at registration or in the protocol. Indicate the stage of the overview of reviews at which amendments were made. | | Additional file table 2 |
| Support | 24 | Describe sources of financial or non-financial support for the overview of reviews, and the role of the funders or sponsors in the overview of reviews. | | Line 525 |
| Competing interests | 25 | Declare any competing interests of the overview of reviews' authors. | | Line 530 |
| Author information | 26a | Provide contact information for the corresponding author. | | Line 12 all |
|  | 26b | Describe the contributions of individual authors and identify the guarantor of the overview of reviews. | | Line 519-523 |
| Availability of data and other materials | 27 | Report which of the following are available, where they can be found, and under which conditions they may be accessed: template data collection forms; data collected from included systematic reviews and supplemental primary studies; analytic code; any other materials used in the overview of reviews. | | Additional file |

Table 2 Deviations from the protocol

| **1** | AxSpA and thyroid autoimmunity were added to the list of included exposure conditions. Thyroid autoimmunity additionally included the presence of thyroglobulin antibodies (TgAb) or thyroid peroxidase antibodies (TPOab) |
| --- | --- |
| **2** | Instead of the preferred reporting items for systematic review and meta-analysis (PRISMA) checklist, the PRIOR checklist was used as it is specific for the reporting of umbrella reviews(46, 47). |

# Table 3 Search Strategy OVID MEDLINE

| Ovid MEDLINE(R) ALL | |
| --- | --- |
|  |  |
| 1 | exp Autoimmune Diseases/ |
| 2 | (auto?immune adj2 (disease* or condition*)).mp. |
| 3 | exp Psoriasis/ |
| 4 | psoria$.mp. |
| 5 | exp Vitiligo/ |
| 6 | vitiligo.mp. |
| 7 | (leucoderma or leukoderma).mp. |
| 8 | exp Alopecia Areata/ |
| 9 | alopecia areata.mp. |
| 10 | ((alopecia adj totalis) or (alopecia adj universalis)).mp. |
| 11 | exp Lupus Erythematosus, Systemic/ |
| 12 | (systemic lupus or sle).mp. |
| 13 | arthritis psoriatic.mp. or exp Arthritis, Psoriatic/ |
| 14 | Ankylosing spondylitis.mp. or exp Spondylitis, Ankylosing/ |
| 15 | Spondylarthritis/ |
| 16 | (axial adj2 spondylarthritis).tw. |
| 17 | (axial adj2 spa).tw. |
| 18 | (ankylos$ or spondyl$).tw. |
| 19 | (bekhterev$ or bechterew$).tw. |
| 20 | (Marie adj struempell$).tw. |
| 21 | (AS or axSPA).tw. |
| 22 | Sacroiliitis/ |
| 23 | ((axial or spin$ or peripheral or vertebral or enthesitis) adj3 (joint$ or spondyloarthritis or arthritis or ankylosing)).tw. |
| 24 | exp Arthritis, Rheumatoid/ |
| 25 | ((rheumatoid or reumatoid or revmatoid or rheumatic or reumatic or revmatic or rheumat$ or reumat$ or revmarthrit$) adj3 (arthrit$ or artrit$ or diseas$ or condition$ or nodule$)).mp. |
| 26 | sjogren syndrome.mp. or exp Sjogren's Syndrome/ |
| 27 | exp Celiac Disease/ |
| 28 | (c?eliac adj disease*).mp. |
| 29 | inflammatory bowel disease.mp. or exp Inflammatory Bowel Diseases/ |
| 30 | ulcerative colitis.mp. or exp Colitis, Ulcerative/ |
| 31 | Crohn's disease.mp. or exp Crohn Disease/ |
| 32 | multiple sclerosis.mp. or exp Multiple Sclerosis/ |
| 33 | Systemic Sclerosis.mp. |
| 34 | exp Scleroderma, Systemic/ |
| 35 | ((systemic adj scler?) or sclero?).mp. |
| 36 | crest syndrome.mp. or exp CREST Syndrome/ |
| 37 | graves disease.mp. or exp Graves Disease/ |
| 38 | (grave? adj3 (diseas? or thyrotoxicos? or hyperthyr?)).mp. |
| 39 | exp Thyroiditis, Autoimmune/ |
| 40 | autoimmune thyroid.mp. |
| 41 | Hashimoto Disease.mp. |
| 42 | exp Hashimoto Disease/ |
| 43 | (thyroid adj autoantibodies).mp. [mp=title, book title, abstract, original title, name of substance word, subject heading word, floating sub-heading word, keyword heading word, organism supplementary concept word, protocol supplementary concept word, rare disease supplementary concept word, unique identifier, synonyms] |
| 44 | thyroid.mp. and exp autoantibodies/ |
| 45 | myasthenia gravis.mp. or exp Myasthenia Gravis/ |
| 46 | addison disease.mp. or exp Addison Disease/ |
| 47 | type 1 diabetes.mp. or exp Diabetes Mellitus, Type 1/ or ("typ? 1 diabet*" or "typ? I diabet*" or "typ?1 diabet*" or "typ?I diabet*").mp. or (("insulin* depend*" or "insulin?depend*") not ("non‐insulin* depend*" or "non insulindepend*")).mp. or (IDDM or T1DM or T1D).mp. or autoimmune diabetes.mp. |
| 48 | exp Pregnancy Complications/ |
| 49 | (pregnancy adj complication?).mp. |
| 50 | exp Abortion, Spontaneous/ |
| 51 | ((recurrent adj2 miscarr?) or miscarr?).mp. |
| 52 | (early adj3 pregnancy loss?).mp. |
| 53 | miscarriage.mp. |
| 54 | exp Stillbirth/ |
| 55 | (stillbirth or (still adj birth)).mp. |
| 56 | exp Fetal Death/ |
| 57 | ((f?etal adj death$) or (f?etal adj demise$)).mp. |
| 58 | exp Hypertension, Pregnancy-Induced/ |
| 59 | (gestational hypertension or (pregnancy adj3 hypertensi$)).mp. |
| 60 | (preeclampsia or pre-eclampsia).mp. |
| 61 | exp Pre-Eclampsia/ |
| 62 | (eclampsia or tox?emia).mp. |
| 63 | exp Eclampsia/ |
| 64 | hellp.mp. or exp HELLP Syndrome/ |
| 65 | exp Placenta Accreta/ |
| 66 | (placenta adj accreta).mp. |
| 67 | placenta percreta.mp. |
| 68 | placenta increta.mp. |
| 69 | (morbidly adj3 adherent placenta).mp. |
| 70 | abnormally invasive placenta.mp. |
| 71 | exp Abruptio Placentae/ |
| 72 | Placenta? abruption.mp. |
| 73 | exp Placenta Previa/ |
| 74 | Placenta pr?evia.mp. |
| 75 | low lying placenta.mp. |
| 76 | exp Hyperemesis Gravidarum/ |
| 77 | Hyperemesis Gravidarum.mp. |
| 78 | exp Morning Sickness/ |
| 79 | morning sickness.mp. |
| 80 | exp Diabetes, Gestational/ |
| 81 | GDM.mp. |
| 82 | (((pregnan$ or gestation$ or prenatal$ or antenatal$ or pre-natal$ or ante-natal$ or maternal$) adj2 diabet$) or gestational diabetes).mp. |
| 83 | ((tub$$ adj3 pregnanc$) or (cornual adj3 pregnanc$) or (heterotopic adj3 pregnanc$) or (abdomin$ adj3 pregnanc$) or (extrauterine adj3 pregnanc$) or (interstitial adj3 pregnanc$) or (cervi$ adj3 pregnanc$) or (ovar$ adj3 pregnanc$) or (cesarean scar adj3 pregnanc$)).mp. |
| 84 | exp Pregnancy, Ectopic/ |
| 85 | ectopic pregnancy.mp. |
| 86 | exp Gestational Trophoblastic Disease/ |
| 87 | gestational trophoblastic.mp. |
| 88 | exp Hydatidiform Mole/ |
| 89 | ((hydatid? adj2 mole?) or (molar adj2 pregnanc?)).mp. |
| 90 | exp Choriocarcinoma/ |
| 91 | choriocarcinoma.mp. |
| 92 | exp Pregnancy, Multiple/ |
| 93 | ((pregnanc* or gestation*) adj (twin* or triplet* or quadruplet* or quintuplet* or multiple or multi?f?et*)).mp. |
| 94 | (Monochorionic or dichorionic).mp. |
| 95 | exp Postpartum Hemorrhage/ |
| 96 | (postpartum hemorrhage or post partum hemorrhage or postpartum haemorrhage or post partum haemorrhage).mp. |
| 97 | obstetric haemorrhage.mp. |
| 98 | obstetric labor, premature.mp. |
| 99 | exp Obstetric Labor, Premature/ |
| 100 | (premature labor or premature labour or preterm labor or preterm labour or preterm birth).mp. |
| 101 | exp Cesarean Section/ |
| 102 | exp Cesarean Section, Repeat/ |
| 103 | (caesarean or cesarean or caesarian or cesarian or cesarien or caesarien or c-section or c section).mp. |
| 104 | cesarean.mp. |
| 105 | exp Extraction, Obstetrical/ |
| 106 | exp Obstetrical Forceps/ |
| 107 | ((operative or instrumental or assisted or forcep* or ventouse* or vacuum*) adj1 (deliver* or birth*)).mp. |
| 108 | exp Infant, Low Birth Weight/ |
| 109 | low birth weight.mp. |
| 110 | (low birth weight* adj4 very low birth weight*).mp. |
| 111 | exp Infant, Small for Gestational Age/ |
| 112 | (small adj3 gestational age).mp. |
| 113 | (intra?uterine growth adj2 (restriction* or retardation)).mp. |
| 114 | iugr.ti,ab. |
| 115 | exp Fetal Growth Retardation/ |
| 116 | (fetal growth adj2 (restriction? or retardation)).mp. |
| 117 | exp Depression, Postpartum/ |
| 118 | postpartum depression.mp. |
| 119 | ((postpartum* or post partum* or post-partum* or postnatal* or post natal* or post-natal* or perinatal* or peri natal* or peri-natal* or puerp*) and (depress* or dysthymi* or adjustment disorder* or mood disorder* or affective disorder*)).mp. |
| 120 | ((postpartum* or post partum* or post-partum* or postnatal* or post natal* or post-natal or perinatal* or peri natal* or peri-natal* or puerp*) and (psychos#s or psychotic)).mp. |
| 121 | (psychosis adj3 after childbirth).mp. |
| 122 | (((third or fourth or 3rd or 4th) adj degree) and tear*).mp. |
| 123 | (((anal near adj2 sphincter) or (rectal adj mucosa) or rectum or (anal adj epithelium) or anus or (recto?vaginal adj2 fistulae) or (anorectal adj mucosa) or (anal adj skin)) and (tear* or injur* or damage* or lacerat* or rupture* or trauma)).mp. |
| 124 | ((obstetric* and anal and sphincter and injur*) or (anal and sphincter and injur*)).mp. |
| 125 | (exp Pregnancy/ or exp Obstetrics/ or (pregnan* or obstetric*).mp.) and (exp Cholestasis/ or exp Cholestasis, Intrahepatic/) |
| 126 | pelvic girdle pain.mp. or exp Pelvic Girdle Pain/ |
| 127 | (symphysis pubis adj3 (pain$ or dysfunction$)).mp. |
| 128 | Pubic Symphysis Diastasis.mp. or exp Pubic Symphysis Diastasis/ |
| 129 | Sacroiliac joint dysfunction.mp. |
| 130 | PGP.mp. |
| 131 | pregnancy outcome.mp. or exp Pregnancy Outcome/ |
| 132 | 1 or 2 or 3 or 4 or 5 or 6 or 7 or 8 or 9 or 10 or 11 or 12 or 13 or 14 or 15 or 16 or 17 or 18 or 19 or 20 or 21 or 22 or 23 or 24 or 25 or 26 or 27 or 28 or 29 or 30 or 31 or 32 or 33 or 34 or 35 or 36 or 37 or 39 or 40 or 41 or 42 or 43 or 44 or 45 or 46 or 47 |
| 133 | 48 or 49 or 50 or 51 or 52 or 53 or 54 or 55 or 56 or 57 or 58 or 59 or 60 or 61 or 62 or 63 or 64 or 65 or 66 or 67 or 68 or 69 or 70 or 71 or 72 or 73 or 74 or 75 or 76 or 77 or 78 or 79 or 80 or 81 or 82 or 83 or 84 or 85 or 86 or 87 or 88 or 89 or 90 or 91 or 92 or 93 or 94 or 95 or 96 or 97 or 98 or 99 or 100 or 101 or 102 or 103 or 104 or 105 or 106 or 107 or 108 or 109 or 110 or 111 or 112 or 113 or 114 or 115 or 116 or 117 or 118 or 119 or 120 or 121 or 122 or 123 or 124 or 125 or 126 or 127 or 128 or 129 or 130 |
| 134 | 132 and 133 |
| 135 | (systematic$ adj2 (review$ or overview)).ti,ab. |
| 136 | (systematic$ adj5 review$).tw,sh. |
| 137 | meta-analysis.mp. or exp meta-analysis/ |
| 138 | 135 or 136 or 137 |
| 139 | 134 and 138 |

# Table 4 List of excluded studies

| **No** | **Year and Author** | **Year** | **Title** | **Reason for exclusion** |
| --- | --- | --- | --- | --- |
|  | Anderson | 2023 | Andersen ML, Jølving LR, Stenager E, Knudsen T, Nørgård BM. Maternal Multiple Sclerosis and Health Outcomes Among the Children: A Systematic Review. Clin Epidemiol. 2023;15:375-89. | Low quality |
|  | Balsells | 2009 | Balsells M, Garcia-Patterson A, Gich I, Corcoy R. Maternal and fetal outcome in women with type 2 versus type 1 diabetes mellitus: a systematic review and metaanalysis. Journal of Clinical Endocrinology & Metabolism. 2009;94(11):4284-91. | Comparator group different |
| 1. 13 | Bansal | 2020 | Pregnancy and lupus nephritis in developing countries: A systematic review | No comparator group |
|  | Bobotis | 2016 | Bobotsis R, Gulliver W, Monaghan K, Lynde C, Fleming P. Psoriasis and adverse pregnancy outcomes: a systematic review of observational studies. British Journal of Dermatology. 2016;175(3):464-72. | High overlap |
|  | Bundhun | 2018 | Bundhun PK, Soogund MZS, Huang F. Arterial/venous thrombosis, fetal loss and stillbirth in pregnant women with systemic lupus erythematosus versus primary and secondary antiphospholipid syndrome: a systematic review and meta-analysis. BMC Pregnancy & Childbirth. 2018;18(1):212. | Comparator group different |
| 1. 14 | Cellini | 2020 | Recurrent Pregnancy Loss in Women with Hashimoto's Thyroiditis with Concurrent Non-Endocrine Autoimmune Disorders | Not a systematic review |
|  | Chaudhary | 2019 | Pregnancy outcomes in a long-term follow-up cohort of pediatric onset systemic lupus erythematosus (PSLE) at a tertiary care center in North-West India | Study design |
|  | Crawford | 2016 | Thyroid Autoimmunity and Reproductive Function | Not a systematic review |
| 1. 43 | Dama | 2016 | Dama M, Steiner M, Lieshout RV. Thyroid peroxidase autoantibodies and perinatal depression risk: A systematic review. Journal of Affective Disorders. 2016;198:108-21. | High overlap |
| 1. 15 | Derakhshan | 2020 | Association of maternal thyroid function with birthweight: a systematic review and individual-participant data meta-analysis | Not required exposure |
| 1. 44 | Dong | 2019 | Dong Y, Dai Z, Wang Z, Wang H, Yuan F, Zhu Y, et al. Risk of gestational diabetes mellitus in systemic lupus erythematosus pregnancy: a systematic review and meta-analysis. BMC Pregnancy & Childbirth. 2019;19(1):179. | High overlap |
| 1. 3 | Essouma | 2020 | Outcomes of systemic lupus erythematosus pregnancies and associated factors in sub-Saharan Africa: A systematic scoping review | Not a systematic review |
| 1. 41 | Finkelsztejn | 2011 | Finkelsztejn A, Brooks JB, Paschoal FM, Jr., Fragoso YD. What can we really tell women with multiple sclerosis regarding pregnancy? A systematic review and meta-analysis of the literature. BJOG: An International Journal of Obstetrics & Gynaecology. 2011;118(7):790-7. | Critically low study quality |
| 1. 16 | Foulon |  | Defining the Most Appropriate Delivery Mode in Women with Inflammatory Bowel Disease: A Systematic Review | Outcome not of interest |
| 1. 17 | Gizzo, S. | 2013 | An update on diabetic women obstetrical outcomes linked to preconception and pregnancy glycemic profile: a systematic literature review | Outcome not of interest |
| 1. 18 | Gonzalez-Blanco, | 2010 | Glycaemic control and pregin women with type 1 diabetes: A systematic review and meta-analysis comparison between lispro and regular insulin | Outcome not of interest |
| 1. 4 | Grygiel-Gorniak | 2011 | Rheumatic Diseases in Reproductive Age-the Possibilities and the Risks | Not a systematic review |
| 1. 19 | Hage, M. P. | 2010 | The link between thyroid function and depression | Population not pregnant women |
| 1. 39 | Hamroun | 2020 | Hamroun S, Hamroun A, Bigna JJ, Allado E, Forger F, Molto A. Fertility and pregnancy outcomes in women with spondyloarthritis: A systematic review and meta-analysis. Annals of the Rheumatic Diseases. 2020;79(SUPPL 1):742-3. | High overlap |
| 1. 5 | Hashash | 2015 | Pregnancy and inflammatory bowel disease | Not a systematic review |
| 1. 6 | Hayslett | 1992 | The effect of systemic lupus erythematosus on pregnancy and pregnancy outcome | Not a systematic review |
| 1. 21 | He | 2015 | Effect of thyroid autoantibodies per se on pregnancy outcomes in euthyroid women undergoing IVF/ICSI | Population not pregnant women |
| 1. 20 | He, H. | 2016 | Effect of thyroid autoimmunity per se on assisted reproduction treatment outcomes: A meta-analysis | Population not pregnant women |
| 1. 22 | Houtchens | 2016 | A review of observational studies of women with MS and pregnancy | Not a systematic review |
|  | Huang | 2022 | Huang W, Wu T, Jin T, Zhang Y, Wang J, Qi J, et al. Maternal and fetal outcomes in pregnant women with rheumatoid arthritis: a systematic review and meta-analysis. Clinical Rheumatology. 2022:1-16. | High overlap |
| 1. 7 | Ideguchi | 2013 | Pregnancy outcomes in Japanese patients with SLE: retrospective review of 55 pregnancies at a university hospital | Study design |
| 1. 23 | Jaffar | 2021 | Type 1 Diabetes in Pregnancy: A Review of Complications and Management | Not a systematic review |
| 1. 24 | Jia, M | 2019 | Meta-analysis of the association between maternal subclinical hypothyroidism and gestational diabetes mellitus | Exposure not of interest |
| 1. 8 | Kane.S | 2003 | Inflammatory bowel disease in pregnancy | Not a systematic review |
| 1. 25 | Kent | 2021 | Is the link between elevated TSH and gestational diabetes mellitus dependant on diagnostic criteria and thyroid antibody status: a systematic review and meta-analysis | Exposure not of interest |
|  | Kim | 2021 | Kim MA, Kim YH, Chun J, Lee HS, Park SJ, Cheon JH, et al. The Influence of Disease Activity on Pregnancy Outcomes in Women With Inflammatory Bowel Disease: A Systematic Review and Meta-Analysis. Journal of Crohn's & colitis. 2021;15(5):719-32. | Exposure not of interest |
| 1. 9 | Lamah | 2002 | Inflammatory bowel disease and pregnancy | Not a systematic review |
| 1. 26 | Laube | 2021 | Assisted Reproductive Technology in Crohn's Disease and Ulcerative Colitis: A Systematic Review and Meta-Analysis | Population not pregnant women |
|  | Levia | 2017 | Leiva P, Schwarze JE, Vasquez P, Ortega C, Villa S, Crosby J, et al. There is no association between the presence of anti-thyroid antibodies and increased reproductive loss in pregnant women after ART: a systematic review and meta-analysis. JBRA Assisted Reproduction. 2017;21(4):361-5. | Population group not appropriate |
| 1. 49 | Li M Wang | 2016 | Li M, Wang SW, Huang S, Mao Y. Relationship between the thyroid autoimmunity and the risk of preterm birth in pregnant women: a meta-analysis. [Chinese]. Zhonghua fu chan ke za zhi. 2016;51(5):339-44. | High overlap |
| 1. 27 | Lopezeon | 2020 | A systematic review and meta-analyses of pregnancy and fetal outcomes in women with multiple sclerosis. IMI2 conception | Exposure not of interest |
| 1. 11 | McDonald | 2018 | Monitoring of Systemic Lupus Erythematosus Pregnancies: A Systematic Literature Review | Not a systematic review |
|  | Meissner | 2021 | Meissner Y, Rudi T, Fischer-Betz R, Strangfeld A. Pregnancy in women with psoriatic arthritis: A systematic literature review of disease activity and adverse pregnancy outcomes. Seminars in Arthritis & Rheumatism. 2021;51(3):530-8. | Comparator group different |
| 1. 28 | Mintziori | 2012 | Thyroid diseases and female reproduction | Not a systematic review |
| 1. 29 | Mintziori | 2016 | The impact of thyroid autoimmunity on IVF/ICSI outcome: Re-evaluation of the findings | Not a systematic review |
| 1. 12 | Mokbel | 2021 | Pregnancy outcomes in women with ankylosing spondylitis: a scoping literature and methodological review | Not a systematic review |
| 1. 30 | Moroni | 2022 | The impact of preeclampsia in lupus nephritis | Not a systematic review |
| 1. 31 | Munoz Munoz | 2022 | Comparing pregnancy outcomes in patients with criteria and non-criteria autoimmune disease: A systematic review | Exposure not of interest |
| 1. 36 | Nazarpur | 2016 | Nazarpour S, Ramezani Tehrani F, Simbar M, Azizi F. Thyroid autoantibodies and the effect on pregnancy outcomes. Journal of Obstetrics & Gynaecology. 2016;36(1):3-9. | Critically low quality- |
|  | Negro | 2011 | Negro R. Thyroid autoimmunity and pre-term delivery: Brief review and meta-analysis. Journal of Endocrinological Investigation. 2011;34(2):155-8. | High overlap |
| 1. 10 | Ogallar | 2018 | Myasthenia gravis and pregnancy. [Spanish] | Not a systematic review |
| 1. 32 | Pacu | 2013 | Thyroid antibodies and risk of Preterm delivery: A meta-analysis of prospective cohort studies | Full text not found |
| 1. 33 | Park | 2016 | The influence of disease activity on birth outcomes in patients with inflammatory bowel disease: Meta-analysis | Duplicate of included study |
| 1. 34 | Petri | 1998 | Pregnancy in SLE | Not a sysytematic review |
| 1. 35 | Piccoli | 2013 | Type 1 diabetes, diabetic nephropathy, and pregnancy: a systematic review and meta-study | Not a sysytematic review |
| 1. 39 | Picconi | 2020 | Piccioni MG, Tabacco S, Giannini A, Deroma M, Logoteta A, Monti M. Myasthaenia gravis in pregnancy, delivery and newborn. Minerva Ginecologica. 2020;72(1):30-5. | Critically low study quality |
| 1. 37 | Prummel | 2004 | Prummel MF, Wiersinga WM. Thyroid autoimmunity and miscarriage. European Journal of Endocrinology. 2004;150(6):751-5. | Critically low quality- |
| 1. 42 | Schmidt | 2022 | Schmidt PMS, Longoni A, Pinheiro RT, Assis AM. Postpartum depression in maternal thyroidal changes. Thyroid Research. 2022;15(1) (no pagination). | High overlap |
|  | Sim (44) | 2023 | Sim BL, Daniel RS, Hong SS, Matar RH, Ganiel I, Nakanishi H, et al. Pregnancy Outcomes in Women With Rheumatoid Arthritis: A Systematic Review and Meta-analysis. JCR: Journal of Clinical Rheumatology. 2023;29(1):36-42. | High overlap |
|  | Smyth | 2010 | Smyth A, Oliveira GH, Lahr BD, Bailey KR, Norby SM, Garovic VD. A systematic review and meta-analysis of pregnancy outcomes in patients with systemic lupus erythematosus and lupus nephritis. Clinical Journal of The American Society of Nephrology: CJASN. 2010;5(11):2060-8. | Comparator group different |
|  | Tian | 2023 | Tian L, Zhang Z, Mao Y, Zong M. Association between pregnant women with rheumatoid arthritis and preeclampsia: A systematic review and meta-analysis. Medicine (Baltimore). 2023;102(26):e34131. | High overlap |
|  | Toulis | 2010 | Toulis KA, Goulis DG, Venetis CA, Kolibianakis EM, Negro R, Tarlatzis BC, et al. Risk of spontaneous miscarriage in euthyroid women with thyroid autoimmunity undergoing IVF: a meta-analysis. European Journal of Endocrinology. 2010;162(4):643-52. | Population group different |
|  | Wang | 2012 | Wang P, Wang Z, He X, Xu D, Wang B. Thyroid antibodies and risk of preterm delivery: A meta-analysis of prospective cohort studies. European Journal of Endocrinology. 2012;167(4):455-64. | High overlap |
|  | Wu | 2015 | Wu H, Hong T, Gao H, Wang H. [Effects of thyroid autoimmunity on pregnancy outcomes in euthyroid women receiving in vitro fertilization: a meta-analysis]. Chung-Hua i Hsueh Tsa Chih [Chinese Medical Journal]. 2015;95(46):3770-4. | Population group different |
|  | Yang | 2015 | Yang Y, Li Q, Wang Q, Ma X. Thyroid antibodies and gestational diabetes mellitus: a meta-analysis. Fertility & Sterility. 2015;104(3):665-71.e3. | High overlap |

# Table 5 Data extraction form

| Study ID |
| --- |
| PDF available |
| Authors |
| Year |
| Geographical area |
| Review aims and objectives |
| Databases searched |
| Additional information source (e.g grey literature) |
| Search period |
| Population |
| Health care setting |
| Exposure |
| Comparator |
| Covariates |
| Defination of exposure |
| Definition of outcomes |
| Data synthesis methods |
| Quality assesment tool |
| Publication year range for included studie |
| Countries included |
| Study design |
| Number of studies included in qualitative analysis (narrative synthesis where meta-analysis was not done / possible by the review authors |
| Number of meta-analysis |
| Outcomes |
| Summar estimates |
| Number of studies included in each meta-analysis |
| Number exposed |
| Events exposed |
| Number unexposed |
| Events unexposed |
| Number of participants (if reported) |
| Author's conclusion |

# Table 6 Quality Assessment of included studies using AMSTAR 2 Tool

|  | 1 | 2 | 3 | 4 | 5 | 6 | 7 | 8 | 9 | 10 | 11 | 12 | 13 | 14 | 15 | 16 |  |
| --- | --- | --- | --- | --- | --- | --- | --- | --- | --- | --- | --- | --- | --- | --- | --- | --- | --- |
| 1. Arafa 2021 | Yes | Yes | Yes | Yes | Yes | Yes | Yes | Yes | Yes | No | Yes | Yes | Yes | Yes | Yes | Yes | Moderate |
| 1. Arvanitakis 2023 | Yes | Yes | Yes | Yes | Yes | Partial Yes | Yes | Yes | Yes | No | Yes | Yes | Yes | Yes | Yes | Yes | Moderate |
| 1. Banner 2022 | No | Yes | Yes | Yes | Yes | No | Yes | Yes | Yes | No | No MA | No MA | No | Yes | No MA | Yes | Low |
| 1. Blagojevic 2020 | Yes | Partial Yes | Yes | Yes | Yes | Yes | Yes | No | Yes | No | Yes | No | No | No | Yes | No | Low |
| 1. Bundhun 2017 | No | No | No | Yes | Yes | Yes | Yes | Yes | Yes | No | Yes | No | Yes | No | Yes | Yes | Low |
| 1. Chen 2011 | Yes | No | Yes | Partial Yes | No | Yes | No | Partial Yes | No | No | Yes | Yes | Yes | No | Yes | Yes | Low |
| 1. Cornish,2007 | Yes | Partial Yes | Yes | Yes | Yes | Yes | No | Yes | Yes | Yes | Yes | Yes | Yes | Yes | Yes | Yes | Moderate |
| 1. Dong 2020 | Yes | Partial Yes | No | Yes | Yes | Yes | Yes | Yes | Yes | No | Yes | Yes | Yes | Yes | Yes | Yes | Moderate |
| 1. Dong,2020 | Yes | No | Yes | Yes | Yes | Yes | Yes | Yes | Yes | Yes | Yes | Yes | Yes | Yes | No | Yes | Low |
| 1. Geng 2022 | Yes | Yes | Yes | Yes | Yes | Yes | Yes | Yes | Yes | Yes | Yes | Yes | Yes | Yes | Yes | Yes | Moderate |
| 1. Hew 2020 | Yes | No | No | Yes | Yes | Yes | Yes | Yes | Yes | No | Yes | Yes | Yes | No | Yes | No | Low |
| 1. He X 2012 | Yes | Yes | Yes | Partial Yes | Yes | Yes | No | Yes | Yes | Yes | Yes | Yes | No | Yes | Yes | Yes | Moderate |
| 1. Korevar 2020 | Yes | Yes | Yes | Yes | No | Yes | Partial Yes | Yes | Yes | Yes | Yes | Yes | Yes | Yes | Yes | Yes | Moderate |
| 1. Leung 2021 | Yes | Yes | Yes | Yes | Yes | Yes | Yes | Yes | Yes | Yes | Yes | Yes | Yes | Yes | Yes | Yes | Moderate |
| 1. Li M 2016 | Yes | Yes | Yes | Partial Yes | No | Yes | Partial Yes | Yes | Yes | No | Yes | Yes | Yes | Yes | Yes | Yes | Moderate |
| 1. Lou J 2021 | Yes | Yes | No | Partial Yes | No | Yes | No | Yes | Yes | No | Yes | Yes | Yes | Yes | Yes | Yes | Low |
| 1. Maguire 2020 | Yes | Yes | Yes | Yes | Yes | Yes | Yes | Yes | Yes | Yes | Yes | Yes | Yes | Yes | Yes | Yes | Moderate |
| 1. Milandi 2020 | Yes | Yes | Yes | Yes | Yes | No | Partial Yes | Yes | Yes | Yes | Yes | Yes | Yes | Yes | Yes | Yes | Low |
| 1. Modrego 2021 | Yes | Yes | Yes | Yes | Yes | Yes | No | Partial Yes | Yes | Yes | No  MA | c | Yes | Yes | No MA | Yes | Low |
| 1. O Toole 2015 | Yes | Yes | Yes | Yes | Yes | No | Yes | Yes | Yes | No | Yes | Yes | Yes | Yes | Yes | Yes | Moderate |
| 1. Saccone 2016 | Yes | Yes | Yes | Yes | Yes | Yes | Yes | Yes | Yes | No | Yes | Yes | No | Yes | Yes | No | Low |
| 1. Talvera 2021 | Yes | Yes | Yes | Yes | Yes | Yes | Yes | Yes | Yes | No | Yes | Yes | No | Yes | No | Yes | Moderate |
| 1. Tandon 2020 | Yes | Yes | Yes | Yes | Yes | Yes | Yes | Yes | Yes | No | Yes | Yes | Yes | Yes | No | Yes | Moderate |
| 1. Tersingini 2014 | Yes | No | No | Yes | Yes | Yes | Yes | No | No | No | Yes | No | No | Yes | Yes | Yes | Low |
| 1. Thangaratinam | Yes | Partial Yes | Yes | Yes | Yes | Yes | Partial Yes | Yes | Yes | Yes | Yes | Yes | Yes | Yes | No | Yes | Moderate |
| 1. Tong 2016 | Yes | Yes | Yes | Partial Yes | No | No | Partial Yes | Yes | Yes | Yes | Yes | Yes | Yes | Yes | Yes | Yes | Moderate |
| 1. Upala 2015 | Yes | Yes | Yes | Partial Yes | No | No | Partial Yes | Yes | Yes | Yes | Yes | Yes | Yes | Yes | Yes | Yes | Moderate |
| 1. Wei 2017 | Yes | Yes | Yes | Yes | Yes | Yes | Yes | Yes | Yes | Yes | Yes | Yes | Yes | Yes | Yes | Yes | Moderate |
| 1. Xie 2021 | Yes | Yes | No | Yes | Yes | Yes | No | Yes | Yes | Yes | Yes | Yes | Yes | Yes | Yes | Yes | Moderate |
| 1. Yu 2016 | Yes | No | Yes | Yes | Yes | Yes | Yes | Yes | Yes | No | Yes | Yes | Yes | No | Yes | Yes | Low |
| 1. Zhang 2017 | Yes | No | Yes | Yes | Yes | No | Yes | Yes | Yes | No | Yes | No | Yes | Yes | Yes | No | Low |
| 1. Jiamin 2023 | Yes | Yes | Yes | Yes | Yes | Yes | Yes | Yes | Yes | Yes | Yes | Yes | Yes | Yes | Yes | Yes | Moderate |
| **Overlapping Excluded Reviews** | | | | | | | | | | | | | | | | | |
| 1. Bobotis,2016 | Yes | Yes | Yes | Yes | Yes | No | No | Partial Yes | Partial Yes | Yes | No  MA | No  MA | No | No | No  MA | Yes | Low |
| 1. Dama, 2016 | Yes | No | Yes | Yes | Yes | Yes | No | Yes | Yes | Yes | No  MA | No  MA | No | Yes | No | No | Low |
| 1. Dong, 2019 | Yes | No | Yes | Yes | Yes | Yes | No | Yes | Yes | Yes | Yes | Yes | Yes | Yes | Yes | Yes | Low |
| 1. Hamroun, 2020 | Yes | No | Yes | Partial Yes | Yes | Yes | No | Yes | Yes | Yes | Yes | Yes | Yes | Yes | Yes | Yes | Low |
| 1. Li M,2016 | Yes | No | Yes | Partial Yes | Yes | Yes | No | Yes | Yes | No | Yes | Yes | No | Yes | Yes | Yes | Low |
| 1. Negro, 2011 | Yes | Partial Yes | No | Partial Yes | No | No | No | Partial Yes | Partial Yes | No | Yes | No | No | No | No | No | Low |
| 1. Schmidt, 2022 | Yes | Yes | Yes | Yes | Yes | No | No | Partial Yes | Partial Yes | Yes | No  MA | No  MA | No | No | No  MA | Yes | Low |
| 1. Wang, 2012 | Yes | No | Yes | Yes | Yes | Yes | No | Yes | Yes | Yes | No  MA | No  MA | No | Yes | No  MA | No | Low |
| 1. Yang 2015 | Yes | No | Yes | Yes | Yes | Yes | No | Yes | Yes | Yes | Yes | Yes | Yes | Yes | Yes | Yes | Low |
| 1. Huang 2022 | Yes | Yes | Yes | Partial Yes | Yes | Yes | Partial Yes | Yes | Yes | Yes | Yes | Yes | Yes | Yes | Yes | Yes | Moderate |
| 1. Sim 2023 | Yes | Yes | Yes | Yes | No | No | Yes | Yes | Yes | No | Yes | Yes | No | Yes | No | No | Low |
| 1. Tian 2023 | Yes | Yes | Yes | Yes | No | No | Yes | Yes | Yes | No | Yes | Yes | No | Yes | No | No | Low |
| **Excluded systematic reviews** | | | | | | | | | | | | | | | | | |
| 1. Finkelsztejn,2011 | Yes | No | No | No | No | Yes | No | No | No | No | No | No | No | No | No | Yes | V.low |
| 1. Nazarpour,2016 | Yes | No | No | No | Yes | Yes | No | No | No | No | No  MA | No  MA | No | No | No  MA | No | V.low |
| 1. Piccioni,2020 | Yes | No | No | No | Yes | Yes | No | No | No | No | No  MA | No  MA | No | No | No  MA | No | V.low |
| 1. Prummel 2004 | Yes | No | No | No | No | Yes | No | No | No | No | No | No | No | No | No | Yes | V.low |
| 1. Anderson 2023 | Yes | No | No | No | No | Yes | No | No | No | No | No | No | No | No | No | Yes | V.low |

No MA= No meta-analysis. Item 1: inclusion of PICO elements? Item 2: review methods established before conduct of review? Item 3: explanation for selection of study designs to be included in review? Item 4: use of a comprehensive search strategy? Item 5: selection of studies in duplicate? Item 6: data extraction in duplicate? Item 7: provision of list of excluded studies with justification for exclusion? Item 8: description of included studies in adequate detail? Item 9: satisfactory technique for risk of bias? Item 10: sources of funding for included studies reported? Item 11: proper methods for metaanalysis? Item 12: potential risk of bias in included studies discussed? Item 13: risk of bias accounted for in interpreting results? Item 14: heterogeneity discussed? Item 15: if meta-analysis conducted was publication bias discussed? Item 16: disclosure of funding or conflict of interest?

Table 7 Overall study citation matrix showing the areas of overlap between autoimmune conditions and pregnancy outcomes.

|  | **AxSpA** | **Coeliac disease** | **IBD** | **Psoriasis** | **Psoriatic arthritis** | **SLE** | **Rheumatoid arthritis** | **Sjögren’s syndrome** | **Thyroid autoimmunity**  **(all)** | **Thyroid autoimmunity (TPO)** |
| --- | --- | --- | --- | --- | --- | --- | --- | --- | --- | --- |
| _Miscarriage_ |  | Arvanitakis 2022  Tersingini 2014  22% |  | Bobotis 2016  Xie 2021  37.5% |  |  | Sim 2023  Jiamin 2023  37.5% |  | Chen 2011  Vanden 2011  25.9% |  |
| _Recurrent miscarriage_ |  |  |  |  |  |  |  |  | Dong 2020  Vanden 2011  21% |  |
| _Pre-eclampsia_ | Maguire2020  Hamroun2020  57% | Sacoone 2016  Tersingini 2014  Arvanitakis 2022  28.5% |  |  | Xie 2021  Hamrounn 2020  20% |  | Huang 2022  Sim 2023  Jiamin 2023  Tian 2023  31% |  |  |  |
| _Gestational diabetes mellitus_ |  |  |  |  |  | Hew 2020  Dong 2019  33% | Huang 2022  Sim 2023  Jiamin 2023  Tian 2023  20% |  | Yang 2015  Lou 2021  20% |  |
| _Caesarean section_ | Maguire2020  Hamroun2020  54% |  | Cornish 2007  Tandon 2020  7% | Bobotis 2016  Xie 2021  36.3% | Xie 2021  Hamrounn 2020  30% | He W2020  Bundhun 2017  0% | Huang 2022  Sim 2023  Jiamin 2023  Tian 2023  37% |  |  |  |
| _Preterm birth_ | Maguire2020  Hamroun2020  30.7% | Sacoone 2014  Tersingini 2016  Arvanitakis 2022  25% | Cornish 2007  Leung 2021  O’Toole 2015  20.9% | Bobotis 2016  Xie 2021  15% | Xie 2021  Hamrounn 2020  20% | He W2020  Wei 2017  Bundhun 2017  3.1% | Huang 2022  Sim 2023  Jiamin 2023  Tian 2023  23.2% | Upala 2016  Geng 2022  16% | HeX 2012  LiM 2014  Wang 2016  41% | HeX 2012 Zhang 2017  Korevar 2020 Thang.2011  Negro 2011  LiM 2014 13% |
| _Intra uterine growth ret._ |  | Sacoone 2014  Tersingini 2016  Arvanitakis 2022  29% |  |  |  |  |  |  |  |  |
| _Small for gestational age_ | Maguire2020  Hamroun2020  30% | Sacoone 2014  Tersingini 2016  0% | Cornish 2007  Leung 2021  O’Toole 2015  22% |  | Xie 2021  Hamroun 2020  14.2% | He W2020  Bundhun 2017  0% | Huang 2022  Sim 2023  Jiamin 2023  Tian 2023  32.3% |  |  |  |
| _Stillbirth_ |  | Sacoone 2014  Tersingini 2016  Arvanitakis 2022  42.8% | Cornish 2007  Tandon 2020  18% |  |  |  | Sim 2023  Jiamin 2023  20% |  |  |  |
| _Low birth weight_ |  | Sacoone 2014  Tersingini 2016  20% | Cornish 2007  Leung 2021  7% | Bobotis 2016  Xie 2021  14.2% |  | He W2020  Bundhun 2017  0% | Sim 2023  Jiamin 2023  30.7% |  |  |  |
|  | High CCA and high overlap (CCA >10%) one of the reviews chosen based on the criteria | | | | | | | | | |
|  | Low CCA(CCA<10%), non -overlapping association | | | | | | | | | |
|  | No overlapping reviews | | | | | | | | | |

CCA = Corrected covered area. Calculation = CCA (%) = N-r/ rc-r: Where N = number of included publications (sum of checked boxes), r = number of rows (primary studies), c = number of columns (number of systematic review

**Table 7.1- Overlapping association between studies looking at association of axial spondyloarthropathy and pre-eclampsia, Caesarean section, small for gestational age and preterm birth**

| Axial spondyloarthropathy | | | |  |
| --- | --- | --- | --- | --- |
| Axial spondyloarthropathy and pre-eclampsia | Maguire 2020 | Hamroun2020 | |  |
| Outcome | Pre-eclampsia | Pre-eclampsia | |  |
| 1. Jakobsson GL, 2016 | X | X | |  |
| 1. Mork S, Voss A 2019 | X |  | |  |
| 1. Park EH 2019 |  | X | |  |
| 1. Park 2019 | X | X | |  |
| 1. Zbinden A 2018 | X | X | |  |
| 1. Smith CJF 2020 | X | X | |  |
| 1. Ostensen M 1983 |  | X | |  |
|  | 5 | 6 | |  |
| CCA-57% |  |  | |  |
| Axial spondyloarthropathy and Caesarean section |  |  | |  |
|  | Caesarean section | Caesarean section | |  |
| 1. Jakobsson GL 2016 | X | X | |  |
| 1. Kristjansdottir SR 2019 | X |  | |  |
| 1. Mork S, Voss A 2019 | X |  | |  |
| 1. Ostensen 1998 | X |  | |  |
| 1. Ostensen 2004 | X |  | |  |
| 1. Ostensen M 1983 | X | X | |  |
| 1. Park EH 2019 | X | X | |  |
| 1. Smith CJF 2020 | X | X | |  |
| 1. Timur H 2016 | X | X | |  |
| 1. Zbinden A 2018 | X | X | |  |
| 1. Zhou 2012 | X |  | |  |
|  | 11 | 6 | |  |
| CCA-54.5% |  |  | |  |
| Outcome | Small for gestational age | | Small for gestational age | |
| 1. Jakobsson GL, 2016 | X | | X | |
| 1. Mork S, Voss A 2019 | X | |  | |
| 1. Park 2019 | X | | X | |
| 1. Strouse 2019 | X | |  | |
| 1. Timur H 2016 |  | | X | |
| 1. Zbinden A 2018 | X | | X | |
|  | 5 | | 4 | |
| CCA-66.6% |  | |  | |
| Axial spondyloarthropathy and Preterm birth |  |  |  |  |
|  | Preterm birth | | Preterm birth | |
| 1. Conor 1980 | X | |  | |
| 1. Jakobsson GL 2016 | X | | X | |
| 1. Kristjansdottir SR 2019 | X | |  | |
| 1. Mork S Voss A 2019 | X | |  | |
| 1. Ostensen 1998 | X | |  | |
| 1. Ostensen M 1983 | X | | X | |
| 1. Park EH 2019 | X | | X | |
| 1. Smith CJF 2020 | X | | X | |
| 1. Strouse J 2019 | X | |  | |
| 1. Timur 2016 |  | |  | |
| 1. Timur H 2016 | X | | X | |
| 1. Zbinden A 2018 | X | | X | |
| 1. Zhou 2012 | X | |  | |
|  | 11 | | 6 | |
| CCA-30.7% |  | |  | |

**Table 7.2- Overlapping association between studies looking at association of Coeliac disease and Pre-eclampsia, Misscarriage, intra-uterine growth restriction, stillbirth, small for gestational age and preterm birth and low birth weight**

| Coeliac disease and miscarriage | | |
| --- | --- | --- |
| Primary studies | Tersingini 2014 | Arvanitakis 2022 |
| 1. Greco 2004 | X | X |
| 1. Grode 2018 |  | X |
| 1. Kotze 2020 |  | X |
| 1. Martinelli 2000 | X | X |
| 1. Moleski 2015 |  | X |
| 1. Molteni 1990 |  | X |
| 1. Moleski 2019 |  | X |
| 1. Sher 1996 | X |  |
| 1. Tata 2005 | X |  |
| CCA=22% | 4 | 7 |

| Coeliac disease and Pre-eclampsia | | | |  |
| --- | --- | --- | --- | --- |
| Primary studies | | Sacoone 2016 | Tersingini 2014 | Arvanitakis 2022 |
| Outcome | | Pre-eclampsia | Pre-eclampsia | Pre-eclampsia |
| 1. Abdul sultan 2014 | | X |  |  |
| 1. Greco 2004 | | X |  | X |
| 1. Martinelli 2000 | | X |  |  |
| 1. Sheiner 2006 | | X | X |  |
| 1. Tata 2005 | | X | X | X |
| 1. Celdir 2021 | |  |  | X |
| 1. Elliott 2019 | |  |  | X |
|  | | 5 | 2 | 4 |
| CCA-28.5% | |  |  |  |
| Coeliac disease and intra-uterine growth retardation(IUGR) | | | |  |
| Outcome | Intrauterine growth restriction | | Intrauterine growth restriction | Intrauterine growth restriction |
| 1. Abdul Sultan A,2014 | X | |  |  |
| 1. Gasbarrini 2000 |  | | X |  |
| 1. Greco L 2004 | X | | X | X |
| 1. Kumar 2011 |  | | X | X |
| 1. Ludvigsson JF 2005 |  | | X |  |
| 1. Martinelli P 2000 | X | | X |  |
| 1. Martinrlli 2010 | X | |  |  |
| 1. Norgard B 1999 |  | | X | X |
| 1. Sharma 2007 |  | | X |  |
| 1. Sheiner E 2006 | X | | X | X |
| 1. Tata LJ 2005 | X | |  |  |
| 1. Elliott 2019 |  | |  | X |
|  | 6 | | 8 | 5 |
| CCA-29% | | | |  |
| Coeliac disease and Stillbirth | |  |  |  |
|  | |  |  |  |
| Outcome | | Stillbirth | Stillbirth | Stillbirth |
| 1. Abdul Sultan A 2014 | | X |  | X |
| 1. Greco L 2004 | | X |  |  |
| 1. Martinelli P 2000 | | X | X | X |
| 1. Sher KS 1994 | | X |  | X |
| 1. Tata LJ 2005 | | X | X | X |
| 1. Grode 2018 | |  |  | X |
| 1. Pogacar 2019 | |  |  | X |
| N | | 5 | 2 | 6 |
| CCA-42.8% | |  |  |  |
|  | |  |  |  |
| Coeliac disease and Small for gestational age (SGA) | | | |  |
| Primary studies | |  |  |  |
| Outcome | | Small for gestational age | Small for gestational age |  |
| 1. Greco 2004 | | X |  |  |
| 1. Khashan 2010 | | X |  |  |
| 1. Marterrili 2000 | | X |  |  |
| 1. McCarty 2009 | | X |  |  |
| 1. Salvatore 2007 | |  | X |  |
| 1. Wolf 2008 | |  | X |  |
|  | | 4 | 2 |  |
| CCA 0% | |  |  |  |

| Coeliac disease and Preterm birth | | | | |  | |
| --- | --- | --- | --- | --- | --- | --- |
|  |  | |  | |  | |
| Outcome | Preterm birth | | Preterm birth | | Preterm birth | |
| 1. Abdul Sultan A 2014 | X | |  | | X | |
| 1. Greco L, 2004 | X | |  | |  | |
| 1. Khashan AS, 2010 | X | | X | | X | |
| 1. Ludvigsson JF 2005 | X | |  | | X | |
| 1. Ludvigsson JF 2001 |  | | X | |  | |
| 1. Martinelli P 2000 | X | | X | | X | |
| 1. Salvatore 2007 |  | | X | |  | |
| 1. Elliott 2019 |  | |  | | X | |
| 1. Celdir 2021 |  | |  | | X | |
| 1. Moleski 2015 |  | |  | | X | |
| 1. Nargard 1999 |  | |  | | X | |
| 1. Abecassis 2019 |  | |  | | X | |
|  | 5 | | 4 | | 9 | |
| CCA-25% |  | |  | |  | |
| Coeliac disease and Low birth weight | | | |  | |  |
| Primary studies |  |  | |  | |  |
| Outcome | Low birth weight | Low birth weight | |  | |  |
| 1. Addul sultan 2014 | X |  | |  | |  |
| 1. Ciacci 1996 |  | X | |  | |  |
| 1. Ludvigsson 2005 | X | X | |  | |  |
| 1. Martinelli 2012 | X |  | |  | |  |
| 1. Ozgor 2000 |  | X | |  | |  |
| CCA-20% | 3 | 3 | |  | |  |

**Table 7.3 Overlapping association between studies looking at association of inflammatory bowel disease and, Caesarean section, small for gestational age, still birth, low birth weight, preterm birth**

| Primary studies | | | | Cornish 2007 | | | Tandon 2020 | | |  |  |
| --- | --- | --- | --- | --- | --- | --- | --- | --- | --- | --- | --- |
| Outcome | | | | Caesarean section | | | Caesarean section | | |  |  |
| 1. Bortoli A 2007 | | | |  | | | X | | |  |  |
| 1. Bortoli A 2011 | | | |  | | | X | | |  |  |
| 1. Bush MC 2004 | | | | X | | |  | | |  |  |
| 1. Dominitz JA 2002 | | | | X | | |  | | |  |  |
| 1. Duricova2019 | | | |  | | | X | | |  |  |
| 1. Elbaz G, 2005 | | | | X | | | X | | |  |  |
| 1. Fedorkow DM 1989 | | | | X | | |  | | |  |  |
| 1. Kornfeld D 1997 | | | | X | | |  | | |  |  |
| 1. Leung YP 2015 | | | |  | | | X | | |  |  |
| 1. Moser MA 2000 | | | |  | | | X | | |  |  |
| 1. Oron G 2012 | | | |  | | | X | | |  |  |
| 1. Porter RJ 1986 | | | | X | | |  | | |  |  |
| 1. Yokoyama 2017 | | | |  | | | X | | |  |  |
|  | | | | 6 | | | 8 | | |  |  |
| CCA-7.6% | | | |  | | |  | | |  |  |
| Inflammatory bowel disease and Stillbirth | | | | | | | | | | | |
| Primary studies | | | Cornish 2007 | | | | | O toole 2015 | | | |
| Outcome | | | Stillbirth | | | | | Stillbirth | | | |
| 1. Dominitz JA 2002 | | | X | | | | |  | | | |
| 1. Elbaz G 2005 | | | X | | | | |  | | | |
| 1. Fedorkow DM 1989 | | | X | | | | | X | | | |
| 1. Fonager K 1998 | | |  | | | | | X | | | |
| 1. Kornfeld D 1997 | | | X | | | | | X | | | |
| 1. Mahadevan U 2017 | | |  | | | | | X | | | |
| 1. Nguyen GC 2009 | | |  | | | | | X | | | |
| 1. Norgard B 2000 | | |  | | | | | X | | | |
| 1. Porter 2008 | | |  | | | | | X | | | |
| 1. Stephansson O 2010 | | |  | | | | | X | | | |
| 1. Stephansson O 2011 | | |  | | | | | X | | | |
|  | | | 4 | | | | | 9 | | | |
| CCA-18% | | |  | | | | |  | | | |
| Inflammatory bowel disease and small for gestational age | | | | | | | | | | | |
| IBD and SGA | Cornish 2007 | | | | Leung 2021 | | | O toole 2015 | | |  |
| 1. Cleary 2011 |  | | | |  | | | X | | |  |
| 1. Dominitz JA 2002 | X | | | | X | | | X | | |  |
| 1. Elbaz G 2005 | X | | | | X | | | X | | |  |
| 1. Fedorkov 1989 |  | | | |  | | | X | | |  |
| 1. Freud 2016 |  | | | | X | | |  | | |  |
| 1. Kornfeld D 1997 | X | | | |  | | | X | | |  |
| 1. Leung YP 2015 |  | | | | X | | |  | | |  |
| 1. Lin 2010 |  | | | | X | | | X | | |  |
| 1. Ludvigsson JF 2002 | X | | | |  | | |  | | |  |
| 1. Mahadevan U 2017 |  | | | |  | | | X | | |  |
| 1. Moser MA 2000 | X | | | | X | | |  | | |  |
| 1. Nguyen GC 2009 |  | | | |  | | | X | | |  |
| 1. Oron G 2010 |  | | | | X | | |  | | |  |
| 1. Oron G 2012 |  | | | |  | | | X | | |  |
| 1. Raatikainan 2011 |  | | | | X | | | X | | |  |
| 1. Shand 2016 |  | | | | X | | |  | | |  |
| 1. Stephansson O 2010 |  | | | |  | | | X | | |  |
| 1. Stephansson O 2011 |  | | | |  | | | X | | |  |
| N | 5 | | | | 9 | | | 12 | | |  |
| CCA 22.2% |  | | | |  | | |  | | |  |
| c | | | | | | | | | | | |
| Inflammatory bowel disease and preterm birth | | | | | | | | | | | |
| Primary studies | | Cornish 2007 | | | | Leung 2021 | | | O toole 2015 | | |
|  | | Preterm birth | | | | Preterm birth | | | Preterm birth | | |
| 1. Kornfeld D 1997 | | X | | | |  | | | X | | |
| 1. Fonager K 1998 | |  | | | |  | | | X | | |
| 1. Norgard B 2000 | | X | | | |  | | | X | | |
| 1. Larzilliere 1998 | | X | | | |  | | |  | | |
| 1. Baird DD 1990 | |  | | | |  | | | X | | |
| 1. Bush MC 2004 | |  | | | | X | | | X | | |
| 1. Dominitz JA 2002 | | X | | | | X | | | X | | |
| 1. Elbaz G 2005 | | X | | | | X | | | X | | |
| 1. Ludvigsson JF 2002 | | X | | | |  | | | X | | |
| 1. Moser MA 2000 | |  | | | | X | | |  | | |
| 1. Porter RJ 1986 | | X | | | |  | | | X | | |
| 1. Bortlik M 2013 | |  | | | | X | | |  | | |
| 1. Bortoli A 2011 | |  | | | | X | | | X | | |
| 1. Bortoli A 2007 | |  | | | | X | | |  | | |
| 1. Freud 2016 | |  | | | | X | | |  | | |
| 1. Lin 2010 | |  | | | | X | | |  | | |
| 1. Leung YP 2015 | |  | | | | X | | |  | | |
| 1. Raatikainan 2011 | |  | | | | X | | | X | | |
| 1. Oron G 2012 | |  | | | | X | | | X | | |
| 1. Langaagergarrd 2007 | |  | | | |  | | | X | | |
| 1. Schnitizer 2011 | |  | | | | X | | |  | | |
| 1. Duricova D 2019 | |  | | | | X | | |  | | |
| 1. Shand 2016 | |  | | | | X | | |  | | |
| 1. Sultan 2016 | |  | | | | X | | |  | | |
| 1. Mahadevan U 2017 | |  | | | |  | | | X | | |
| 1. Fedorkov 1989 | |  | | | |  | | | X | | |
| 1. Cleary 2011 | |  | | | |  | | | X | | |
| 1. Mayberry JF 1986 | |  | | | |  | | | X | | |
| 1. Nguyen GC, 2009 | |  | | | |  | | | X | | |
| 1. Norgard B 2003 | |  | | | |  | | | X | | |
| 1. Stephansson O 2010 | |  | | | |  | | | X | | |
| N | | 7 | | | | 16 | | | 21 | | |
| CAA- 20.9% | |  | | | |  | | |  | | |
|  | | | | | | | | | | | |
| Primary studies | | | Cornish 2007 | | | | | Leung 2021 | | | |
| outcome | | | low birth weight | | | | | low birth weight | | | |
| 1. Baird DD 1990 | | | X | | | | |  | | | |
| 1. Dominitz JA 2002 | | |  | | | | | X | | | |
| 1. Duricova D 2019 | | |  | | | | | X | | | |
| 1. Elbaz G 2005 | | | X | | | | | X | | | |
| 1. Emerson 2013 | | |  | | | | | X | | | |
| 1. Freud 2016 | | |  | | | | | X | | | |
| 1. Kornfeld D 1997 | | | X | | | | |  | | | |
| 1. Larzilliere I 1998 | | | X | | | | |  | | | |
| 1. Leung YP 2015 | | |  | | | | | X | | | |
| 1. Lin 2010 | | |  | | | | | X | | | |
| 1. Raatikainan 2011 | | |  | | | | | X | | | |
| 1. Schnitizer 2011 | | |  | | | | | X | | | |
| 1. Sultan 2016 | | |  | | | | | X | | | |
| 1. Yokoyama | | |  | | | | | X | | | |
|  | | | 4 | | | | | 11 | | | |
| CCA-7.1% | | |  | | | | |  | | | |

**Table 7.4 Overlapping association between studies looking at association of Psoriasis and miscarriage, caesarean section, preterm birth, low birth weight**

| Psoriasis and miscarriage | | |
| --- | --- | --- |
| Primary studies | Bobotsis 2016 | Xei 2021 |
| Outcome | Miscarriage | Miscarriage |
| 1. Ben-David G 2008. | X |  |
| 1. Cohen-Barak E 2011 | X | X |
| 1. Kimball AB 2014 | X |  |
| 1. Lima XT 2013 | X | X |
| 1. Polachek A 2017 |  | X |
| 1. Schaufelberg BW 2014 | X |  |
| 1. Seeger JD 2007. | X | X |
| 1. Yurkon K 2014 | X |  |
|  | 7 | 4 |
| CCA-37.5% |  |  |
| Psoriasis and Caesarean section | | |
| **Outcome** | Caesarean section | Caesarean section |
| 1. Amiri 2016 |  | X |
| 1. Bandoli G 2020 |  | X |
| 1. Ben-David G 2008 | X | X |
| 1. Broms G 2018 |  | X |
| 1. Chiou 2017 |  | X |
| 1. Cohen-Barak E 2011 | X | X |
| 1. Lima XT 2013 | X | X |
| 1. Polachek A 2017 |  | X |
| 1. Remaeus K 2019 |  | X |
| 1. Smith CJF 2019 |  | X |
| 1. Yang YW, 2010 | X | X |
|  | 4 | 11 |
| CCA-36.3% | | |
| Psoriasis and Preterm birth | | |
|  | Preterm birth | Preterm birth |
| 1. Amiri 2016 |  | X |
| 1. Bandoli G 2020 |  | X |
| 1. Bandoll and chambers 2017 |  | X |
| 1. Ben-David G 2008 | X |  |
| 1. Broms G 2018 |  | X |
| 1. Cohen-Barak E 2011 | X |  |
| 1. Kimball AB 2014 | X |  |
| 1. Lambe M 2020 |  | X |
| 1. Lima XT 2013 | X | X |
| 1. Remaeus 2019 |  | X |
| 1. Smith CJF 2019 |  | X |
| 1. Strouse J 2019 |  | X |
| 1. Yang YW 2010 | X | X |
| N | 5 | 10 |
| CCA-15.3% |  |  |
| Psoriasis low birth weight | Low birth weight | Low birth weight |
| 1. Ben 2006 |  | X |
| 1. Broms 2018 |  | X |
| 1. Chiou 2017 |  | X |
| 1. Cohen,2017 | X |  |
| 1. Lima 2011 |  | X |
| 1. Smith 2019 |  | X |
| 1. Yang 2011 | X | X |
| CCA 14.2% | 6 | 2 |

**Table 7.5 Overlapping association between studies looking at association of psoriatic arthritis with pre-eclampsia and preterm birth, Caesarean section, small for gestational age**

| Psoriatic arthritis and Pre-eclampsia | | |
| --- | --- | --- |
| Primary studies | Xei 2021 | Hamroun 2020 |
| Outcome | Pre-eclampsia | Pre-eclampsia |
| 1. Amiri 2016 | X |  |
| 1. Bandoli 2017 | X |  |
| 1. Bandoli 2020 | X |  |
| 1. Bro 2018 | X |  |
| 1. Chiou 2017 | X |  |
| 1. Lambe 2020 | X |  |
| 1. Polacheck 2019 |  | X |
| 1. Remaeus 2019 | X | X |
| 1. Smith 2019 | X | X |
| 1. Zbinden 2018 |  | X |
|  | 8 | 4 |
| CCA=20% |  |  |
|  | Caesarean section | Caesarean section |
| 1. Amiri 2016 | X |  |
| 1. Bandoli 2020 |  | X |
| 1. Ben David 2008 | X |  |
| 1. Broms 2018 | X |  |
| 1. Chiou 2017 | X |  |
| 1. Cohen Barak 2010 | X |  |
| 1. Lima 2011 | X |  |
| 1. Polachek 2019 | X | X |
| 1. Remaeus 2019 | X | X |
| 1. Smith 2019 | X | X |
|  | 10 | 3 |
| CCA 30% |  |  |
|  |  |  |
|  | Preterm birth | Preterm birth |
| 1. Amiri 2016 | X |  |
| 1. Bandoli and Chambers 2017 | X |  |
| 1. Bandoli 2020 | X |  |
| 1. Bro 2018 | X |  |
| 1. Lambe 2020 | X |  |
| 1. Lima 2011 | X |  |
| 1. Remaeus 2019 | X | X |
| 1. Smith 2019 | X | X |
| 1. Strouse 2019 | X |  |
| 1. Yang 2011 | X |  |
| CCA 20% | 10 | 2 |
|  |  |  |
|  | Small for gestational age | Small for gestational age |
| 1. Amiri 2016 | X |  |
| 1. Bandoli 2020 | X |  |
| 1. Broms 2017 | X |  |
| 1. Chiou 2017 | X |  |
| 1. Lambe 2020 | X |  |
| 1. Ramaeus 2019 | X | X |
| 1. Strouse 2019 | X |  |
|  | 7 | 1 |
| **CCA=14.2%** |  |  |
|  |  |  |

**Table 7.6 Overlapping association between studies looking at association of Rheumatoid arthritis and pre-eclampsia, gestational diabetes, Caesarean section, small for gestational age and preterm birth**

| Primary studies | Huang  2022 | Sim  2023 | Tian  2023 | Jiamin 2023 |
| --- | --- | --- | --- | --- |
| Outcome | Pre-eclampsia | Pre-eclampsia |  |  |
| 1. Smith 2020 | X |  |  | x |
| 1. Wiliams 2019 | X |  |  |  |
| 1. Aljiary 2018 | X | X | X | X |
| 1. Wallenius2014 | X | X | X |  |
| 1. Norgard 2010 | X | X |  | X |
| 1. Lin 2010 | X | X |  | X |
| 1. Reed 2006 | X | X | X | X |
| 1. Abdulrahman |  | X |  | X |
| 1. Al RayAes |  | X |  | X |
| 1. Bandoli and Chambers 2017 |  | X | X | X |
| 1. Heng-Ching 2009 |  |  | X |  |
| 1. Jogernsen 2009 |  |  | X |  |
| 1. Nogarrd 2010 |  |  | X | X |
| 1. Barnabe 2011 |  |  | X | X |
| 1. Eudy 2018 |  |  |  | X |
| 1. Yang 2021 |  |  |  | x |
| 1. Zbinden 2018 |  |  |  | X |
| 1. Park 2022 |  |  |  | X |
| 1. Singh 2023 |  |  |  |  |
| CCA=31% | 7 | 8 | 8 | 14 |
|  | Gestational diabetes | Gestational diabetes |  | Gestational diabetes |
| 1. Keeling 2020 | X |  |  | X |
| 1. Smith 2020 | X |  |  | X |
| 1. Aljary 2018 | X | X |  | X |
| 1. Norgard 2010 | X |  |  |  |
| 1. Abdulrahman 2020 |  | x |  | X |
| 1. Bandoli and Chambers |  | x |  | X |
| 1. Psofai 2015 |  |  |  | X |
| 1. Eudy 2018 |  |  |  | X |
| 1. Galappatthy 2017 |  |  |  | X |
| 1. Norgard 2010 |  |  |  | X |
| 1. Singh 2023 |  |  |  | X |
| CCA=20% | 4 | 3 |  | 10 |
|  | Caesarean section | Caesarean section |  |  |
| 1. Hellgren 2021 | X |  |  |  |
| 1. Keeling 2020 | X |  |  | X |
| 1. Smith 2020 | X |  |  | X |
| 1. Williams 2019 | X |  |  |  |
| 1. Kishore 2019 | X |  |  |  |
| 1. Zbinden 2018 | X |  |  | X |
| 1. Wallenius 2014 | X | X |  |  |
| 1. Barnabe 2010 | X |  |  | X |
| 1. Norgard 2010 | X | X |  | X |
| 1. Lin 2010 | X | X |  | X |
| 1. Reed 2006 | X | X |  | X |
| 1. Eudy 2005 |  | X |  | X |
| 1. Aljary 2006 |  | X |  | X |
| 1. Al Rayes 2008 |  | X |  | X |
| 1. Abdulrahman 2007 |  | X |  | X |
| 1. Jolving 2018 |  |  |  | X |
| 1. Park 2022 |  |  |  | X |
| 1. Tarplin 2022 |  |  |  | X |
| 1. Singh 2013 |  |  |  | X |
| 1. Bobrica 2023 |  |  |  | X |
| CCA=37.5% | 11 | 8 |  | 16 |
|  | Small for gestational age | Small for gestational age |  |  |
| 1. Hellgren 2021 | X |  |  |  |
| 1. Keeling 2020 | X |  |  | X |
| 1. Williams 2019 | X |  |  |  |
| 1. Strouse 2019 | X |  |  | X |
| 1. Aljary 2018 | X |  |  | X |
| 1. Zbinden 2018 | X |  |  | X |
| 1. Wallenius 2014 | X |  |  |  |
| 1. Ma 2014 | X |  |  | X |
| 1. Barnabe 2011 | X |  |  | X |
| 1. Norgard 2010 | X | X |  | X |
| 1. Lin 2010 | X | X |  | X |
| 1. Reed 2006 | X | X |  | X |
| 1. Jacobsen 2003 | X |  |  |  |
| 1. Jolving 2018 |  |  |  | X |
| 1. Tsai 2022 |  |  |  | X |
| 1. Singh 2022 |  |  |  | X |
| 1. Bobrica 2023 |  |  |  | X |
| CCA=32.3% | 13 | 3 |  | 13 |
|  | Preterm birth | Preterm birth |  | Preterm birth |
| 1. Hellgren 2021 | X |  |  |  |
| 1. Keeeling 2020 | X |  |  | X |
| 1. Smith 2020 | X |  |  | X |
| 1. Williams 2019 | X |  |  |  |
| 1. Strouse 2019 | X |  |  |  |
| 1. Kishore 2019 | X |  |  |  |
| 1. Zbinden 2018 | X |  |  | X |
| 1. Wallenius 2014 | X | X |  |  |
| 1. Rom 2014 | X |  |  |  |
| 1. Ma 2014 | X |  |  | X |
| 1. Barnabe 2011 | X |  |  | X |
| 1. Lin 2010 | X |  |  | X |
| 1. Reed 2006 | X |  |  | X |
| 1. AL Rayes 2005 |  | X |  | X |
| 1. Aljary 2004 |  | X |  | X |
| 1. Bandoli and Chambers 2017 |  | X |  | X |
| 1. Eudy 2005 |  | X |  | X |
| 1. Norgard 2009 |  | X |  | X |
| 1. Strouse 2019 |  |  |  | X |
| 1. Posfai 2015 |  |  |  | X |
| 1. Jolving 2018 |  |  |  | X |
| 1. Knudsen 2020 |  |  |  | X |
| 1. Knudsen 2021 |  |  |  | X |
| 1. Park 2022 |  |  |  | X |
| 1. Tarplin 2022 |  |  |  | X |
| 1. Tsai 2022 |  |  |  | X |
| 1. Singh 2022 |  |  |  | X |
| 1. Bobrica 2023 |  |  |  | x |
|  |  |  |  |  |
| CCA=23.2% | 13 | 6 |  | 22 |
|  |  |  |  |  |
|  | Stillbirth | Stillbirth |  | Stillbirth |
|  |  |  |  |  |
| 1. Al Rayes 2021 |  | X |  | X |
| 1. Eudy |  | X |  |  |
| 1. Norgard 2010 | X | X |  | X |
| 1. Galappatthy 2017 |  |  |  | X |
| 1. Rom 2016 |  |  |  | X |
| 1. Wallenius 2015 | X |  |  | X |
| 1. Tarplin 2022 |  |  |  | x |
| 1. Keeling 2020 | X |  |  |  |
| 1. Strouse 2019 | X |  |  |  |
| 1. Ajiary 2018 | X |  |  |  |
| CCA 20% | 5 | 3 |  | 6 |
|  |  |  |  |  |
|  | Miscarriage |  |  | Miscarriage |
|  |  |  |  |  |
| 1. Wallenius 2015 | X |  |  | X |
| 1. Nathan 1984 | X |  |  | X |
| 1. AL Rayes 2021 |  |  |  | x |
| 1. Eudy 2018 |  |  |  | X |
| 1. Galappatthy 2017 |  |  |  | X |
| 1. Park 2022 |  |  |  | X |
| 1. Tarplin 2022 |  |  |  |  |
| CCA= 14% | 2 |  |  | 6 |
|  |  |  |  |  |
|  | Gestational HT |  |  | Gestational HT |
| 1. Keeling 2020 | X |  |  | X |
| 1. Smith 2019 | X |  |  | x |
| 1. Kishore 2019 | X |  |  |  |
| 1. Barnabe 2010 | X |  |  |  |
| 1. Posfai 2015 |  |  |  | x |
| 1. Galappatthy 2017 |  |  |  | X |
| 1. Rom 2018 |  |  |  | X |
| 1. Aijary 2018 |  |  |  | x |
| CCA=25% | 4 |  |  | 6 |
|  |  |  |  |  |
|  | LBW |  |  |  |
| 1. Strouse 2019 | X |  |  | X |
| 1. Wallenius 2014 | X |  |  |  |
| 1. Ma 2014 | X |  |  | X |
| 1. Lin 2010 | X |  |  | X |
| 1. Reed 2006 | X |  |  | X |
| 1. Posfai 2015 |  |  |  | X |
| 1. Abdulrahman 2020 |  |  |  | X |
| 1. Galappatthy 2017 |  |  |  | X |
| 1. Knudsen 2021 |  |  |  | X |
| 1. Park 2022 |  |  |  | x |
| 1. Tsai 2022 |  |  |  | X |
| 1. Singh 2023 |  |  |  | X |
| 1. Bobrica 2023 |  |  |  | X |
| CCA 30.7% | 5 |  |  | 12 |

Table 7.7 Overlapping association between studies looking at association of Sjögren’s syndrome and preterm birth

| Primary studies | Upala 2016 | Geng 2022 |
| --- | --- | --- |
| 1. Carolis 2014 | X | X |
| 1. Takaya 2011 | X |  |
| 1. Siamopoulou Mavridou 2010 | X |  |
| 1. Skopouli 2008 | X |  |
| 1. Hussein et al 2011 | X | X |
| 1. C Ballester 2016 |  | X |
| 1. L Li 2019 |  | X |
| 1. R Priori 2013 |  | X |
| 1. B Elliott 2019 |  | X |
| 1. JJ Yang 2021 |  | X |
| 1. HJ Haga 2005 |  | X |
| 1. JF Xu 2019 |  | X |
| CCA=16.6% | 5 | 9 |

Table 7.8 Overlapping association between studies looking at association of systemic lupus erythematosus (SLE) and gestational diabetes mellitus (GDM),Preterm birth,Low birth weight,Caesarean section,small for gestational age

| SLE and Gestational diabetes mellitus(GDM) | | |  |
| --- | --- | --- | --- |
|  | He W 2020 | Dong 2019 |  |
|  | Gestational diabetes mellitus | Gestational diabetes mellitus |  |
| 1. Abdwani 2018 | X | X |  |
| 1. Bandoli G 2019 | X |  |  |
| 1. Gallappatthy 2017 |  | X |  |
| 1. Phansenee S 2018 | X | X |  |
| 1. Wu J 2018 |  | X |  |
| 1. Yuen 2008 |  | X |  |
|  | 3 | 5 |  |
| CCA 33% |  |  |  |
| SLE and Preterm birth (PTB) | | | |
|  | He W 2020 | Wei 2017 | Bundhun 2017 |
| Outcome | Preterm birth | Preterm birth | Preterm birth |
| 1. Abdwani 2018 | X |  |  |
| 1. Arkema 2016 |  |  | X |
| 1. Bandoli G 2019 | X |  |  |
| 1. Bernardo 2014 |  | X | X |
| 1. Georgiio 2000 |  | X |  |
| 1. Gnacio 2018 | X |  |  |
| 1. Hamed 2013 |  | X |  |
| 1. Julkunen 1993 |  | X |  |
| 1. Ling 2018 | X |  |  |
| 1. Molokhia 2007 |  | X |  |
| 1. Phansenee S 2018 | X |  |  |
| 1. Wu J 2018 | X |  |  |
| 1. Yan Yuen 2008 |  | X | X |
| 1. Barnabe 2011 |  |  |  |
| 1. Jackobsen 2015 |  |  | X |
| 1. Wallenius 2014 |  |  | X |
|  | 6 | 6 | 5 |
| CCA-3.1% |  |  |  |
| SLE and Low birth weight (LBW) |  |  |  |
|  | Bundhun 2017 | He W 2020 |  |
|  | Low birth weight | Low birth weight |  |
| 1. Arkema 2016 | X |  |  |
| 1. Walleinus 2014 | X |  |  |
| 1. Abdwani 2017 |  | X |  |
| 1. Gnacio 2018 |  | X |  |
| 1. Phasenee 2017 |  | X |  |
| CCA -0% | 2 | 3 |  |
| SLE and Caesarean section |  |  |  |
|  | Bundhun 2017 | He W  2020 |  |
|  |  |  |  |
| 1. Aekema 2016 | X |  |  |
| 1. Bamabe 2011 | X |  |  |
| 1. Nili 2013 | X |  |  |
| 1. Walleinus 2014 | X |  |  |
| 1. Bandoli 2019 |  | X |  |
| 1. Gnacio 2018 |  | X |  |
| 1. Phansenee 2017 |  | X |  |
| 1. Wu 2017 |  | X |  |
|  | 4 | 4 |  |
| CCA 0% |  |  |  |
| SLE and small for gestational age SGA | Bundhun 2017 | HeW  2020 |  |
| 1. Bandoli 2019 |  | X |  |
| 1. Barnabe 2011 | X |  |  |
| 1. Chen 2010 | X |  |  |
| 1. Gnacio 2018 |  | X |  |
| 1. Nili 2013 | X |  |  |
| 1. Phensenee 2017 |  | X |  |
| 1. Wallenius 2014 | X |  |  |
| 1. Wu 2017 |  | X |  |
|  | 4 | 4 |  |
| CCA 0% |  |  |  |

Table 7. 9 Overlapping association between studies looking at association of thyroid autoantibodies(all) and recurrent pregnancy loss, miscarriage, gestational diabetes melitus, postpartum depression, preterm birth

| Thyroid Autoimmunity and recurrent pregnancy loss | | |
| --- | --- | --- |
| Primary studies | Dong 2020 | Vanden 2011 |
| 1. Ashrafi 2007 | X |  |
| 1. Bagis 2001 | X |  |
| 1. Bellver 2008 |  | X |
| 1. Bussen 1997 | X | X |
| 1. Dendrinos 2000 | X | X |
| 1. Esplin 1998 | X |  |
| 1. Iravani 2008 | X | X |
| 1. Kutteh 1999 | X | X |
| 1. Laba 2013 | X |  |
| 1. Lakshmi 2016 | X |  |
| 1. Mecacci 2000 | X | X |
| 1. Motak 2013 | X |  |
| 1. Motak Pochrzest 2013 | X |  |
| 1. Pratt 1993 | X |  |
| 1. Raye Green 2011 | X |  |
| 1. Roberts 1966 | X | X |
| 1. Shoenfield 2008 |  | X |
| 1. Ticconi 2011 | X |  |
| 1. Tulppala 1993 | X |  |
|  | 15 | 8 |
| **CCA-21%** |  |  |
| Thyroid autoimmunity (all) and miscarriage | | |
|  | Chen 2011 | Vanden 2011 |
| 1. Bagis 2007 | X |  |
| 1. Benhadi 2009 |  | X |
| 1. Bussen 1997 | X |  |
| 1. Dendrinos 2000 | X |  |
| 1. Esplin 1998 | X |  |
| 1. Glinoer 1991 | X |  |
| 1. Glinoer 1994 | X |  |
| 1. Iijama 1997 | X | X |
| 1. Iravani 2008 | X |  |
| 1. Kilic 2008 | X |  |
| 1. Kutteh 1999 | X |  |
| 1. Lejeune 1993 | X | X |
| 1. Marai 2004 | X |  |
| 1. Muller 1999 | X |  |
| 1. Negro 2005 | X | X |
| 1. Negro 2006 | X | X |
| 1. Poppe 2004 |  | X |
| 1. Poppe 2003 | X |  |
| 1. Pratt 1993 | X |  |
| 1. Pratt 1993 | X |  |
| 1. Roberts 1998 |  | X |
| 1. Rushworth 2000 |  | X |
| 1. Sezer 2009 | X | X |
| 1. Sieiro 2004 | X | X |
| 1. Singh 1995 |  | X |
| 1. Stagnaro-Green 1990 | X | X |
| 1. Todorova 2008 | X |  |
| CCA-25.9% | 22 | 12 |
| Thyroid autoimmunity(all) and gestational diabetes mellitus(GDM) | | |
|  | Yang 2015 | Lou 2021 |
|  | GDM | GDM |
| 1. Agarwal 2006 | X |  |
| 1. Bhat 2018 |  | X |
| 1. Chen 2014 | X | X |
| 1. Corrales 2014 | X | X |
| 1. Haddow 2016 |  | X |
| 1. Huang 2019 |  | X |
| 1. Kang 2014 | X |  |
| 1. Karakosta 2012 | X | X |
| 1. Kumru 2015 |  | X |
| 1. Li 2013 | X |  |
| 1. Li 2020 |  | X |
| 1. Lin 2014 | X |  |
| 1. Mannisto 2010 | X | X |
| 1. Mannisto 2011 | X |  |
| 1. Montaner 2008 | X | X |
| 1. Negro 2011 | X | X |
| 1. Nokavo 2010 | X |  |
| 1. Oleveri 2000 | X |  |
| 1. Oliveri 1997 | X |  |
| 1. Ortega-Gonazales 2000 | X |  |
| 1. Pradhan 2013 |  | X |
| 1. Sert 2020 |  | X |
| 1. Tang 2013 | X |  |
| 1. Wang 2014 | X |  |
| 1. Wang H2011 | X |  |
| 1. Wang XM 2011 | X |  |
| 1. Ying 2016 |  | X |
| 1. Yuan 2020 |  | X |
| 1. Zhang 2013 | X |  |
| 1. Zhao 2013 | X |  |
| CCA 20% | 21 | 15 |

| Thyroid autoimmunity and postpartum depression | | | | | | |
| --- | --- | --- | --- | --- | --- | --- |
|  | | Dama 2016 | | Schmidt 2018 | | Milandi 2020 |
| 1. Albacar 2010 | | X | | X | | X |
| 1. Bergink 2011 | |  | | X | |  |
| 1. Bunevicius 2009 | | X | |  | |  |
| 1. Groer and Vaughan 2013 | | X | | X | |  |
| 1. Harris 1993 | | X | | X | | X |
| 1. Kent 1999 | |  | | X | |  |
| 1. Kon 1999 | | X | |  | |  |
| 1. Kuijpens 2001 | | X | | X | | X |
| 1. Lambrinoudaki 2010 | |  | | X | |  |
| 1. Lazarus 1996 | | X | | X | |  |
| 1. Le Donne 2012 | | X | | X | |  |
| 1. Oretti 1997 | | X | |  | |  |
| 1. Pedersen 2016 | |  | | X | |  |
| 1. POP 1993 | | X | | X | |  |
| 1. Pop 2006 | | X | |  | | X |
| 1. Ruschi 2008 | |  | | X | |  |
| 1. Sylven 2013 | |  | | X | |  |
| 1. Wesseloo 2018 | |  | | X | | X |
| 1. Zhang 2019 | |  | | X | |  |
|  | | **11** | | 15 | | **5** |
| CCA-31.5% | |  | |  | |  |
| Thyroid autoimmunity (all) and preterm birth | | | | | | |
| Primary studies | He X 2012 | | Li M 2016 | | Li M Wang 2014 | |
| Outcome | preterm birth | | preterm birth | | preterm birth | |
| 1. Ashoor G 2011 | X | | X | |  | |
| 1. Ashoor G 2011-TPOAb | X | |  | |  | |
| 1. Bhattacharya 2016 | X | |  | |  | |
| 1. Chen X 2015 | X | | X | |  | |
| 1. Ghafoor F 2006 | X | | X | | X | |
| 1. Glinoer D 1994 | X | | X | | X | |
| 1. Haddow JE 2010 | X | | X | | X | |
| 1. Iijima T 1997 TGAb | X | | X | |  | |
| 1. Iijima T 1997 TPOAb | X | |  | |  | |
| 1. Jiang YY 2011 | X | |  | |  | |
| 1. Karakosta 2012 |  | |  | | X | |
| 1. Korevar2013 |  | | X | | X | |
| 1. Lan Y 2013 |  | | X | | X | |
| 1. Liu X 2012 |  | | X | | X | |
| 1. Mannisto T 2009-TGAb | X | | X | |  | |
| 1. Mannisto T 2009-TPOAb | X | |  | |  | |
| 1. Nambiar V 2011 | X | | X | | X | |
| 1. Negro R 2006 | X | |  | | X | |
| 1. Negro R 2011 | X | |  | | X | |
| 1. Stagnaro-Green A 1990 | X | | X | |  | |
| 1. Bhattacharya 2016 |  | | X | |  | |
| 1. Saki 2014 |  | | X | |  | |
| 1. Cleary 2008 |  | | X | |  | |
| CCA 41% | 16 | | 16 | | 10 | |

**Table 7.10 Overlapping association between studies looking at association of thyroid peroxidase antibodies (TPO) and preterm birth**

| Thyroid autoimmunity (Thyroid peroxidase antibody) and preterm birth | | | | | | |
| --- | --- | --- | --- | --- | --- | --- |
| Primary studies  TPO and Preterm birth | HeX 2012 | Korevaar 2020 | Negro 2011 | Li M 2014 | Thangaratinam 2011 | Zhang 2017 |
| 1. ABCD |  | X |  |  |  |  |
| 1. ALSPAC |  | X |  |  |  |  |
| 1. Ashnoor G 2011 | X |  |  |  |  | X |
| 1. BIddal |  | X |  |  |  |  |
| 1. Chen |  | X |  |  |  |  |
| 1. Chinese name |  |  |  | X |  |  |
| 1. Chinese names |  |  |  | X |  |  |
| 1. EFSOCH |  | X |  |  |  |  |
| 1. Generation R |  | X |  |  |  |  |
| 1. Ghafoor | X | X | X | X | X |  |
| 1. Girona |  | X |  |  |  |  |
| 1. Glinoer 1994 | X |  | X | X | X |  |
| 1. Haddow |  |  | X | X | X |  |
| 1. HAppy |  | X |  |  |  |  |
| 1. Jiang 2011 | X |  |  |  |  |  |
| 1. Karakosta |  |  |  | X |  |  |
| 1. Korevar |  |  |  | X |  |  |
| 1. Lijima 1997 | X |  | X |  | X |  |
| 1. Mannisto 2009 | X |  | X |  |  |  |
| 1. Mosso |  | X |  |  |  |  |
| 1. Nambiar 2011 | X |  |  | X |  |  |
| 1. Negro 2006 | X |  | X | X | X |  |
| 1. Negro R 2011 | X |  | X | X |  |  |
| 1. NFBC |  | X |  |  |  |  |
| 1. POPOVA |  | X |  |  |  | X |
| 1. Rhea |  | X |  |  |  |  |
| 1. Viva |  | X |  |  |  |  |
| 1. Western Australia |  | X |  |  |  |  |
| 1. Xu 2012 |  |  |  |  |  | X |
| CCA=13.7% | 9 | 15 | 7 | 10 | 5 | 3 |

**Table 7.11 Overlapping association between studies looking at association of preterm birth and ulcerative colitis and Crohn’s disease**

| Preterm birth in ulcerative colitis | Cornish 2007 | Otoole 2015 |
| --- | --- | --- |
| 1. Baird 1990 | X | X |
| 1. Bush 2004 | X | X |
| 1. Dominitz 2002 | X | X |
| 1. Elbaz 2005 | X | X |
| 1. Larzille 2002 | X |  |
| 1. Ludvigson 2002 | X | X |
| 1. Norgard 2000 | X | X |
| 1. Porter 1986 | X | X |
| 1. Norgard 2003 |  | X |
| 1. Langagergarrd 2007 |  | X |
| 1. Nguyen 2009 |  | X |
| 1. Stephansson 2010 |  | X |
| 1. Raatikainen 2011 |  | X |
| 1. Bartolli 2011 |  | X |
| 1. Stephannson 2011 |  | X |
| CCA 53% | 8 | 15 |

| Preterm birth in Crohn’s disease | **Cornish** | **Otoole** |
| --- | --- | --- |
| 1. Baird 1990 | X | X |
| 1. Bush 2004 | X | X |
| 1. Dominitz 2002 | X | X |
| 1. Elbaz 2005 | X | X |
| 1. Larzille | X |  |
| 1. Ludvigson 2002 | X | X |
| 1. Moser 2001 | X |  |
| 1. Fonager 1988 | X | X |
| 1. Porter 1986 | X | X |
| 1. Mayberry 1986 |  | X |
| 1. Norgard 2003 |  | X |
| 1. Langagergarrd 2007 |  | X |
| 1. Nguyen 2009 |  | X |
| 1. Stephansson 2010 |  | X |
| 1. Raatikainen 2011 |  | X |
| 1. Bartolli 2011 |  | X |
| CCA=50% | 9 | 15 |

Table 8-Overlapping and non overlapping associations by corrected covered area (CCA) calculations

| Index | Author,Year | Quality | AI condition | Outcome | Data analysis | CCA | Decision |
| --- | --- | --- | --- | --- | --- | --- | --- |
| 1 | Maguire 2020 | Moderate | Axial spondyloarthropathy | Pre-eclampsia | MA (5) | 57% | Yes |
|  | Hamroun 2020 | Low |  |  | MA (6) |  | No |
| 2 | Maguire 2020 | Moderate | Axial spondyloarthropathy | Caesarean section | MA (11) | 54% | Yes |
|  | Hamroun 2020 | Low |  |  | MA (6) |  | No |
| 3 | Maguire 2020 | Moderate | Axial spondyloarthropathy | Small for gestational age | MA (5) | 66% | Yes |
|  | Hamroun 2020 | Low |  |  | MA (4) |  | No |
| 4 | Maguire 2020 | Moderate | Axial spondyloarthropathy | Preterm birth | MA (11) | 30.7% | Yes |
|  | Hamroun 2020 | Low |  |  | MA (6) |  | No |
|  |  |  |  |  |  |  |  |
| 5 | Tersingini 2014 | Low | Coeliac disease | Miscarriage | MA (4) | 22% | No |
|  | Arvanitakis s 2022 | Moderate |  |  | MA (7) |  | Yes |
| 6 | Saccoone 2016 | Low | Coeliac disease | Pre-eclampsia | MA (5) | 28.5% | No |
|  | Tersingini 2014 | Low |  |  | MA (2) |  | No |
|  | Arvanitakis s 2022 | Moderate |  |  | MA (4) |  | Yes |
| 7 | Saccoone 2016 | Low | Coeliac disease | Intrauterine growth restriction | MA (6) | 29% | No |
|  | Tersingini 2014 | Low |  |  | MA (8) |  | No |
|  | Arvanitakis s 2022 | Moderate |  |  | MA (5) |  | Yes |
| 8 | Saccoone 2016 | Low | Coeliac disease | Small for gestational age | MA (4) | 0% | Yes |
|  | Tersingini 2014 | Low |  |  | MA (2) |  | Yes |
| 9 | Saccoone 2016 | Low | Coeliac disease | Stillbirth | MA (5) | 42.8% | No |
|  | Tersingini 2014 | Low |  |  | MA (2) |  | No |
|  | Arvanitakis s 2022 | Moderate |  |  | MA (6) |  | Yes |
| 10 | Saccoone 2016 | Low | Coeliac disease | Preterm birth | MA (5) | 25% | No |
|  | Tersingini 2014 | Low |  |  | MA (4) |  | No |
|  | Arvanitakis 2022 | Moderate |  |  | MA (9) |  | Yes |
| 11 | Saccoone 2016 | Low | Coeliac disease | Low birth weight | MA (3) | 12% | Yes |
|  | Tersingini 2014 | Low |  |  | MA (3) |  | No |
|  |  |  |  |  |  |  |  |
| 12 | Cornish 2007 | Moderate | Inflammatory bowel disease | Caesarean section | MA (6) | 7.1% | Yes |
|  | Tandon 2020 | Moderate |  |  | MA (8) |  | Yes |
| 13 | Cornish 2007 | Moderate | Inflammatory bowel disease | Stillbirth | MA (4) | 18% | No |
|  | O toole 2015 | Moderate |  |  | MA (9) |  | Yes |
| 14 | Cornish 2007 | Moderate | Inflammatory bowel disease | Small for gestational age | MA (5) | 22% | No |
|  | Leung 2021 | Moderate |  |  | MA (9) |  | Yes |
|  | O toole 2015 | Moderate |  |  | MA  (12) |  | No |
| 15 | Cornish 2007 | Moderate | Inflammatory bowel disease | Preterm birth | MA (7) | 20.9% | No |
|  | Leung 2021 | Moderate |  |  | MA  (16) |  | Yes |
|  | O toole 2015 | Moderate |  |  | MA  (21) |  | No |
| 16 | Cornish 2007 | Moderate | Inflammatory bowel disease | Low birth weight | MA(4) | 14.3% | Yes |
|  | Leung 2021 | Moderate |  |  | MA  (11) |  | Yes |
|  |  |  |  |  |  |  |  |
| 17 | Bobotis 2016 | Low | Psoriasis | Miscarriage | NA  (7) | 37.5% | No |
|  | Xei 2021 | Moderate |  |  | MA  (4) |  | Yes |
| 18 | Bobotis 2016 | Low | Psoriasis | Caesarean section | NA  (5) | 36.3% | No |
|  | Xei 2021 | Moderate |  |  | MA  (10) |  | Yes |
| 19 | Bobotis 2016 | Low | Psoriasis | Preterm birth | NA  (5) | 15% | No |
|  | Xei 2021 | Moderate |  |  | MA  (10) |  | Yes |
| 20 | Bobotis 2016 | Low | Psoriasis | Low birth weight | NA (2) | 14.2% | No |
|  | Xei 2021 | Moderate |  |  | MA (6) |  | Yes |
|  |  |  |  |  |  |  |  |
| 21 | Xei 2021 | Moderate | Psoriasis Arthritis | Pre -eclampsia | MA (8) | 20% | Yes |
|  | Hamroun 2020 | Low |  |  | MA (4) |  | No |
| 22 | Xei 2021 | Moderate | Psoriasis Arthritis | Caesarean section | MA (10) | 30% | Yes |
|  | Hamroun 2020 | Low |  |  | MA (4) |  | No |
| 23 | Xei 2021 | Moderate | Psoriasis A | Preterm birth | MA (10) | 20% | Yes |
|  | Hamroun 2020 | Low |  |  | MA (2) |  | No |
| 24 | Xei 2021 | Moderate | Psoriasis A | Small for gestational age | MA (7) | 14.2% | Yes |
|  | Hamroun 2020 | Low |  |  | MA (1) |  | No |
|  |  |  |  |  |  |  |  |
| 25 | Huang 2022 | Moderate | Rheumatoid arthritis | Pre-eclampsia | MA (7) | 37.5% | Yes |
|  | Sim 2023 | Low |  |  | MA (8) |  | No |
|  | Tian 2023 | Moderate |  |  | MA(8) |  | No |
|  | Jiamin 2023 | Moderate |  |  | MA(14) |  | Yes |
| 26 | Huang 2022 | Moderate | Rheumatoid arthritis | Gestational diabetes mellitus | MA (4) | 20% | No |
|  | Sim 2023 | Low |  |  | MA (3) |  | No |
|  | Jiamin 2023 | Moderate |  |  | MA(10) |  | Yes |
| 27 | Huang 2022 | Moderate | Rheumatoid arthritis | Caesarean section | MA (11) | 37.5% | No |
|  | Sim 2023 | Low |  |  | MA(8) |  | No |
|  | Jiamin 2023 | Moderate |  |  | MA(16) |  | Yes |
| 28 | Huang 2022 | Moderate | Rheumatoid arthritis | Small for gestational age | MA (13) | 23.3% | No |
|  | Sim 2023 | Low |  |  | MA (3) |  | No |
|  | Jiamin 2023 | Moderate |  |  | MA(13) |  | Yes |
| 29 | Huang 2022 | Moderate | Rheumatoid arthritis | Preterm birth | MA(13) | 23.2% | No |
|  | Sim 2023 | Low |  |  | MA(6) |  | No |
|  | Jiamin 2023 | Moderate |  |  | MA(22) |  | Yes |
| 30 | Huang 2022 | Moderate | Rheumatoid arthritis | Gestational hypertension | MA(4) | 25% | No |
|  | Jiamin 2023 | Moderate |  |  | MA() |  | Yes |
| 31 | Jiamin 2023 | Moderate |  | Miscarriage | MA(2) | 14% | Yes |
|  | Huang 2022 | Moderate | Rheumatoid arthritis |  | MA(6) |  | No |
| 32 | Jiamin 2023 | Moderate |  | Low birth weight | MA(12) | 14% | Yes |
|  | Sim 2023 | Low |  |  | MA(5 |  | No |
|  | Huang 2022 | Moderate | Rheumatoid arthritis |  | MA(5) |  | No |
| 33 | Jiamin 2023 | Moderate |  | Stillbirth | MA(5) | 14% | Yes |
|  | Sim 2023 | Low |  |  | MA(3) |  | No |
|  | Huang 2022 | Moderate | Rheumatoid arthritis |  | MA(6) |  | No |
|  |  |  |  |  |  |  |  |
| 34 | Upala 2016 | Moderate | Sjögren’s syndrome | Preterm birth | MA (5) | 16.6% | No |
|  | Geng 2022 | Moderate |  |  | MA (9) |  | Yes |
|  |  |  |  |  |  |  |  |
| 35 | HeW 2020 | Low | SLE | Gestational diabetes mellitus | MA(3)  13,883 | 33% | Yes |
|  | Dong 2019 | Low |  |  | MA (5)  3432 |  | No |
| 36 | Hew 2020 | Low | SLE | Preterm birth | MA(6) | 3.1% | Yes |
|  | Wei 2017 | Low |  |  | MA (6) |  | Yes |
|  | Bundhun 2017 | Low |  |  | MA (5) |  | Yes |
| 37 | Bundhun 2017 | Low | SLE | Caesarean section | MA (4) | 0% | Yes |
|  | HeW 2020 | Low |  |  | MA (4) |  | Yes |
| 38 | Bundhun 2017 | Low | SLE | Small for gestational age | MA (4) | 0% | Yes |
|  | HeW 2020 | Low |  |  | MA (4) |  | Yes |
| 39 | Bundhun 2017 | Low | SLE | Low birth weight | MA (2) | 0% | Yes |
|  | HeW 2020 | Low | SLE |  | MA (3) |  | Yes |
|  |  |  |  |  |  |  |  |
| 40 | Dong | Low | TAA(all) | Miscarriage | MA (15) | 25.9% | Yes |
|  | Vanden 2011 | low |  |  | MA (8) |  | No |
| 41 | Chen 2011 | Moderate | TAA(all) | Recurrent miscarriage | MA (22) | 21% | Yes |
|  | Van den 2011 | Low |  |  | MA (12) |  | No |
| 42 | Yang 2015 | Low | TAA(all) | Gestational diabetes mellitus | MA (21) | 20% | No |
|  | Lou 2021 | Low |  |  | MA (15) |  | Yes |
| 43 | Dama 2016 | Low | TAA(all) | Postpartum depression | Na (11) | 31% | No |
|  | Schmidt 2018 | Low |  |  | NA (15) |  | No |
|  | Minaldi 2020 | Low |  |  | MA (5) |  | Yes |
| 44 | Li M 2016 | Low | TAA(all) | Preterm birth | MA (16) | 41% | Yes |
|  | LiM W 2012 | Low |  |  | MA (10) |  | No |
|  | Hex 2012 | Moderate |  |  | MA (16) |  | No |
| 45 | Negro 2011 | Low | TPO | Preterm birth | NA (7) | 13% | No |
|  | Korevar 2020 | Moderate |  |  | MA (15) |  | Yes |
|  | Thangaratinam 2011 | Moderate |  |  | MA (5) |  | No |
|  | Zhang 2017 | Low |  |  | MA (3) |  | No |
|  | Li M 2014 | Low |  |  | MA (10) |  | No |

Table 9-Forest plots of the meta-analysis performed in the study

**Table 9.1 Coeliac disease**

**Table 10.1.1 Meta-analysis of Sacoone and Tersingini -Looking at associaltion of SGA and Coeliac disease No overlap as Sacoone 2016 included cohort studies and Tersingini 2014 included case control studies.**


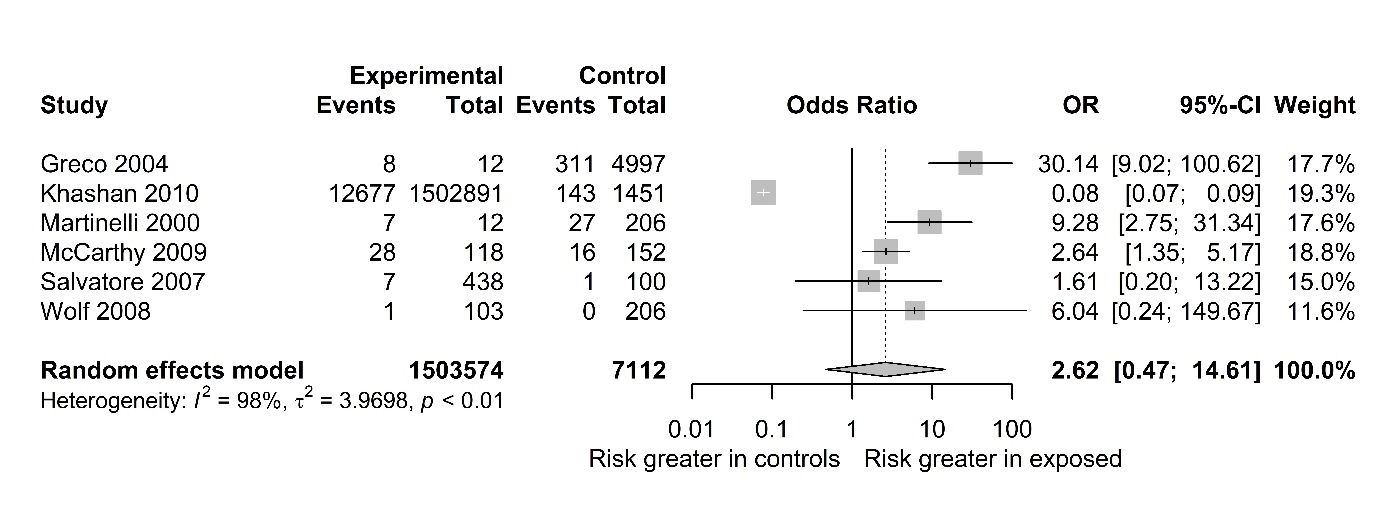


**Table 9.2-IBD**

**Table 10.2.1 Meta-analysis of Leung et al -cohort studies and registries -Looking at associaltion of low birth weight and IBD**


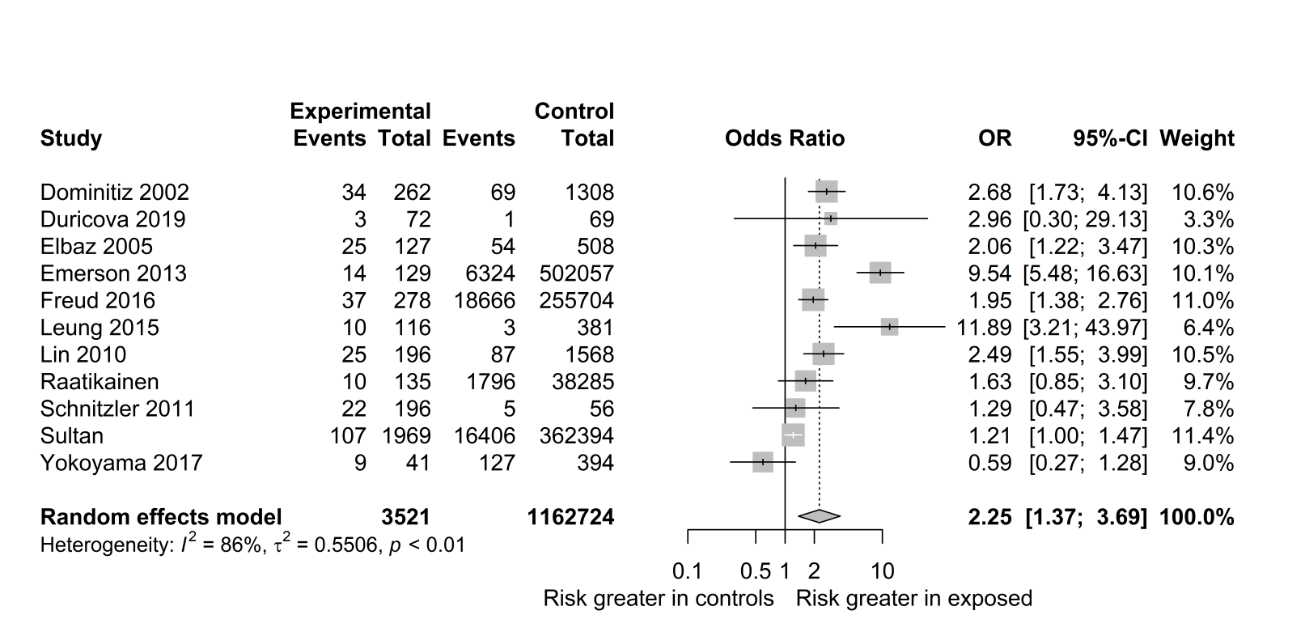


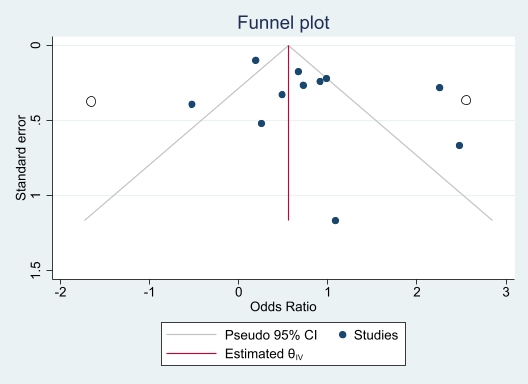


Funnel plot to access the publication bias

**Table 9.2.2 Meta-analysis of Leung et al and Cornish further combined-Looking at associaltion of low birth weight and IBD**


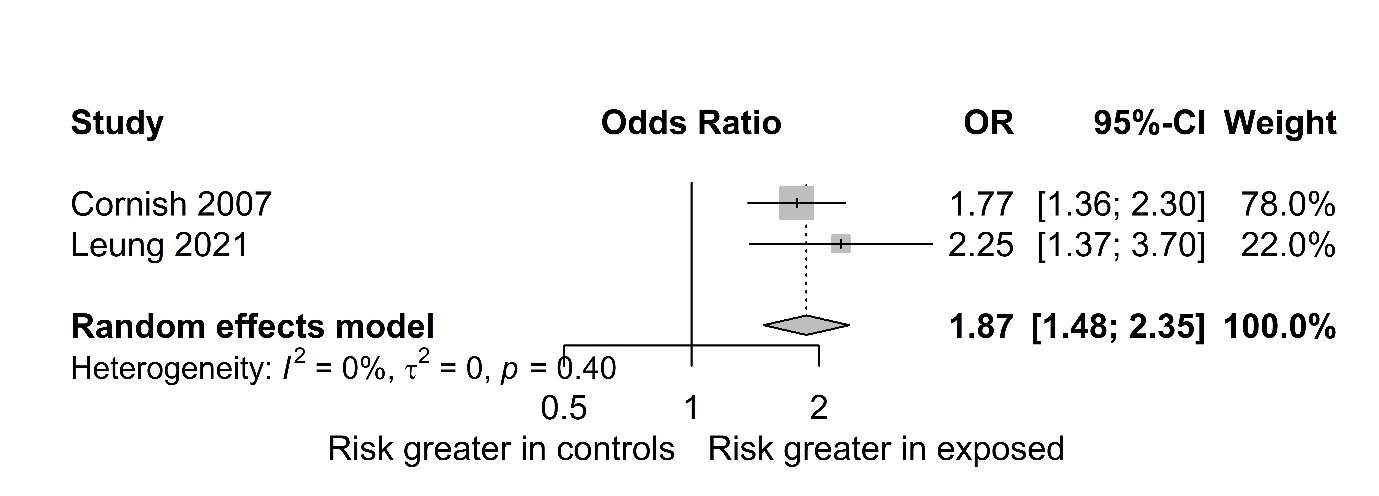


**Table 9.2.3 Meta-analysis of Leung et al -cohort studies and registries -Looking at associaltion of Preterm Birth and IBD**


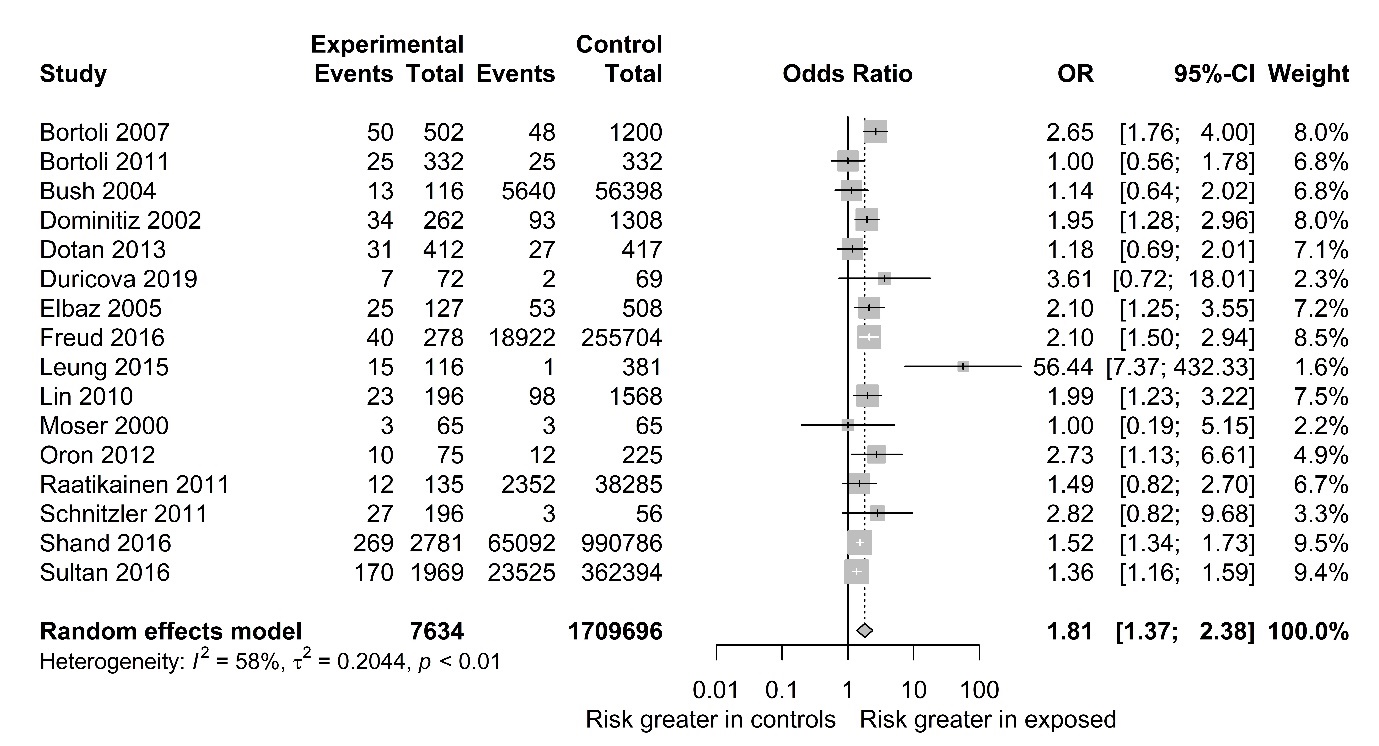


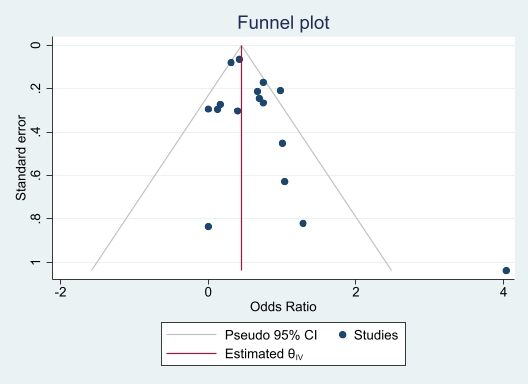


Funnel plot to access the publication bias

**Table 9.2.4 Meta-analysis of Cornish and Tandon Looking at the association of Caesarean section and IBD Slight overlap as Cornish 2007 and Tandon 2020 ,Elbaz 2005 only overlapping study**


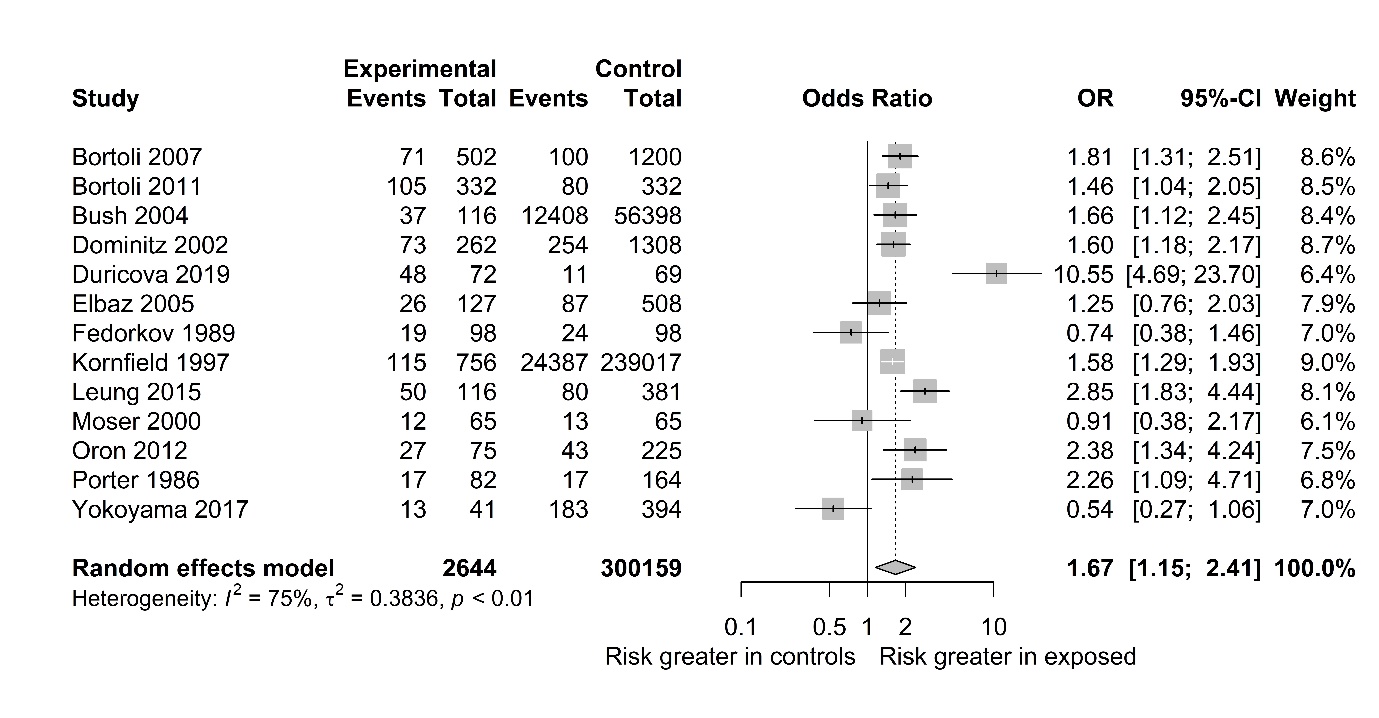


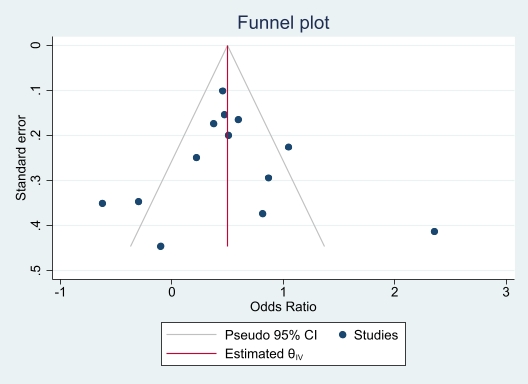


**Table 9.2.5 -Meta-analysis of cohort and case control studies Leung looking at association of SGA and IBD**


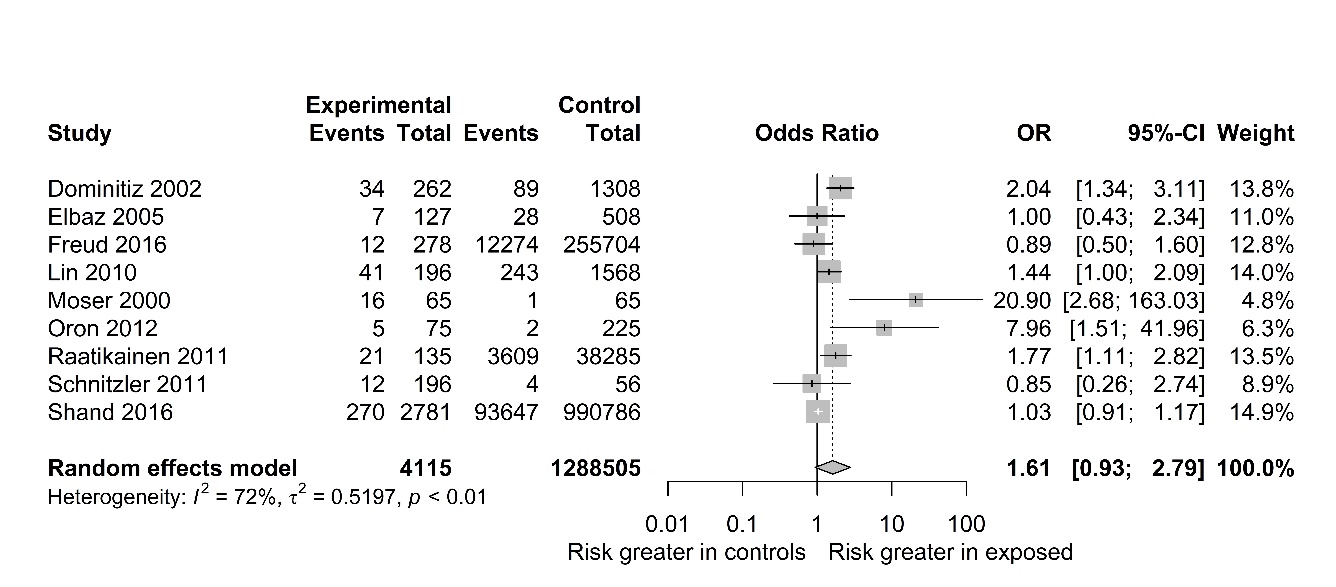


**Table 9.3 forest plot SLE**

**Table 9.3.1 Meta-analysis of Bundhun and HeW Looking at the association of SGA and SLE. No overlap as Bundhun included studies only 2001-2016 and Hew included newer studies**


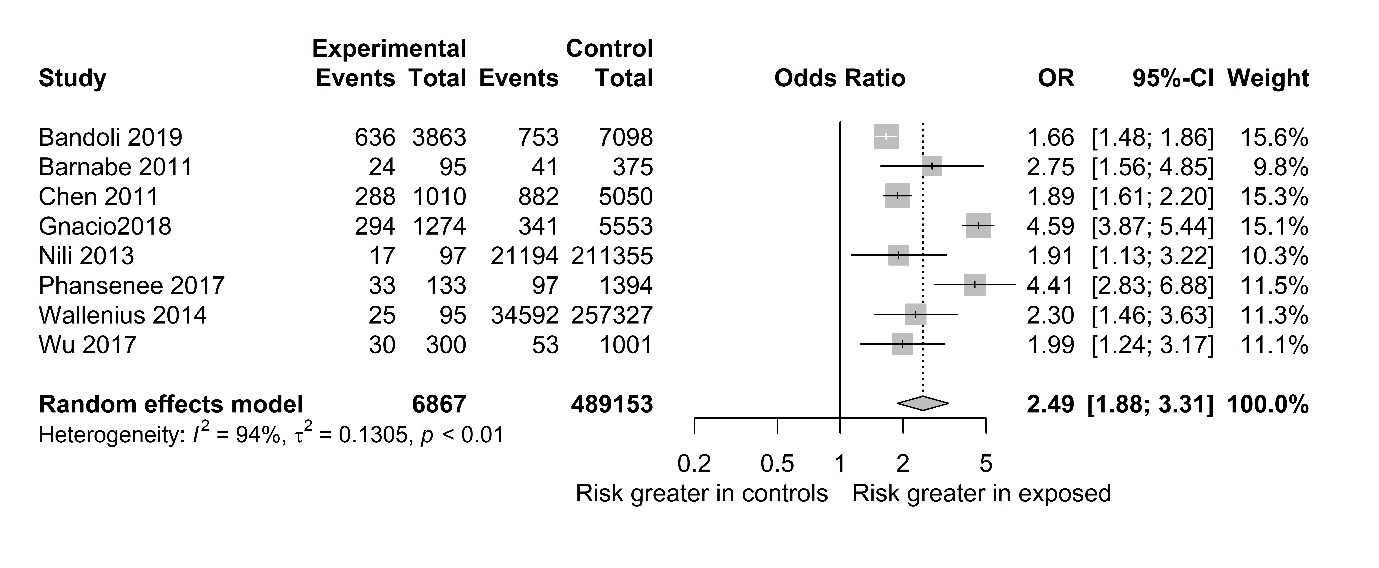


**Table 9.3.2-Meta-analysis of Bundhun and HeW Looking at the association of Caesarean section and SLE No overlap as Bundhun included studies only 2001-2016 and Hew included newer studies**


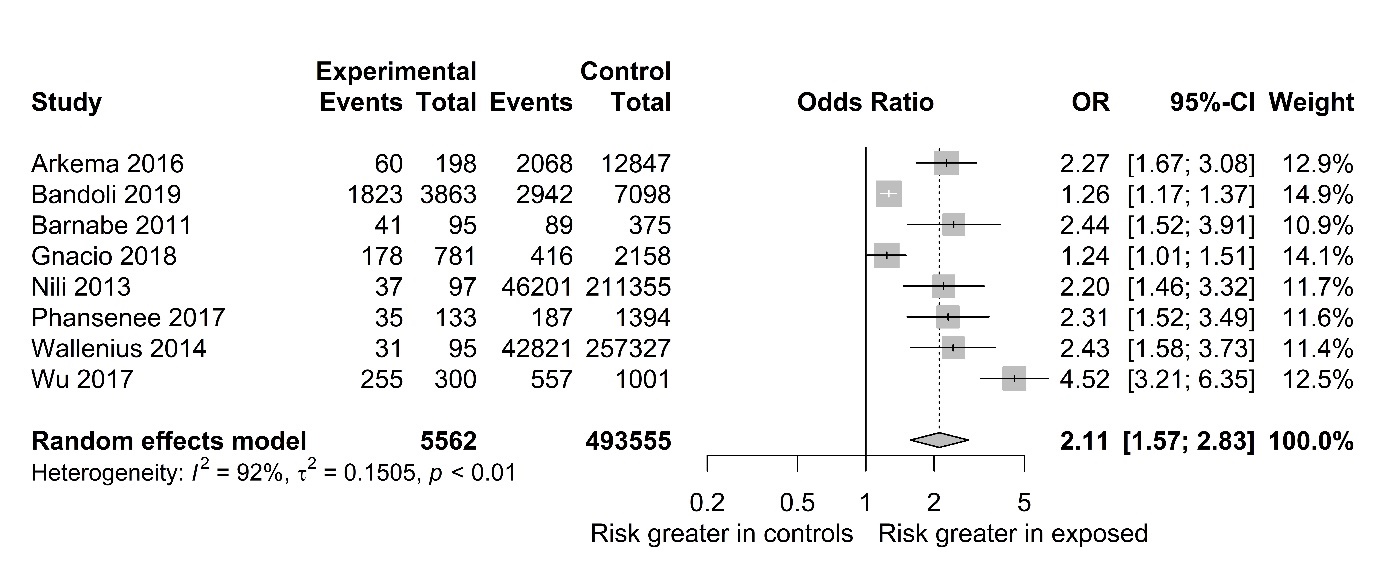


**Table 9.3.3-Meta-analysis of Hew ,Bundhun,Wei association pf PTB and SLE-less overlap as He w searched for studies after 2016,where as Bundhun 2001-2016 and Wei before 2016 and searched different databases,Wei also was looking at other outcomes**

**Bernardo 2014 and Yuen overlapp studies**


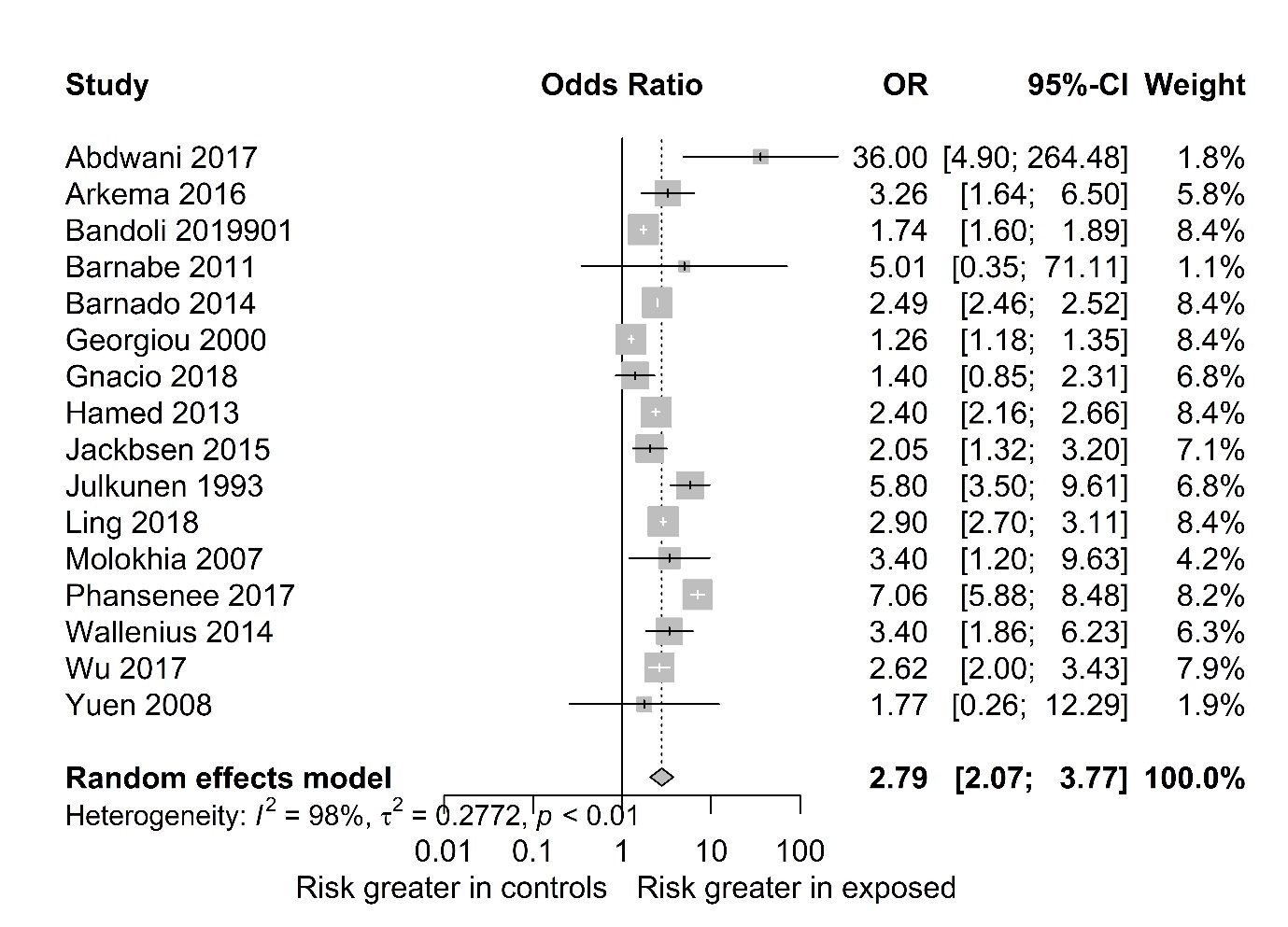


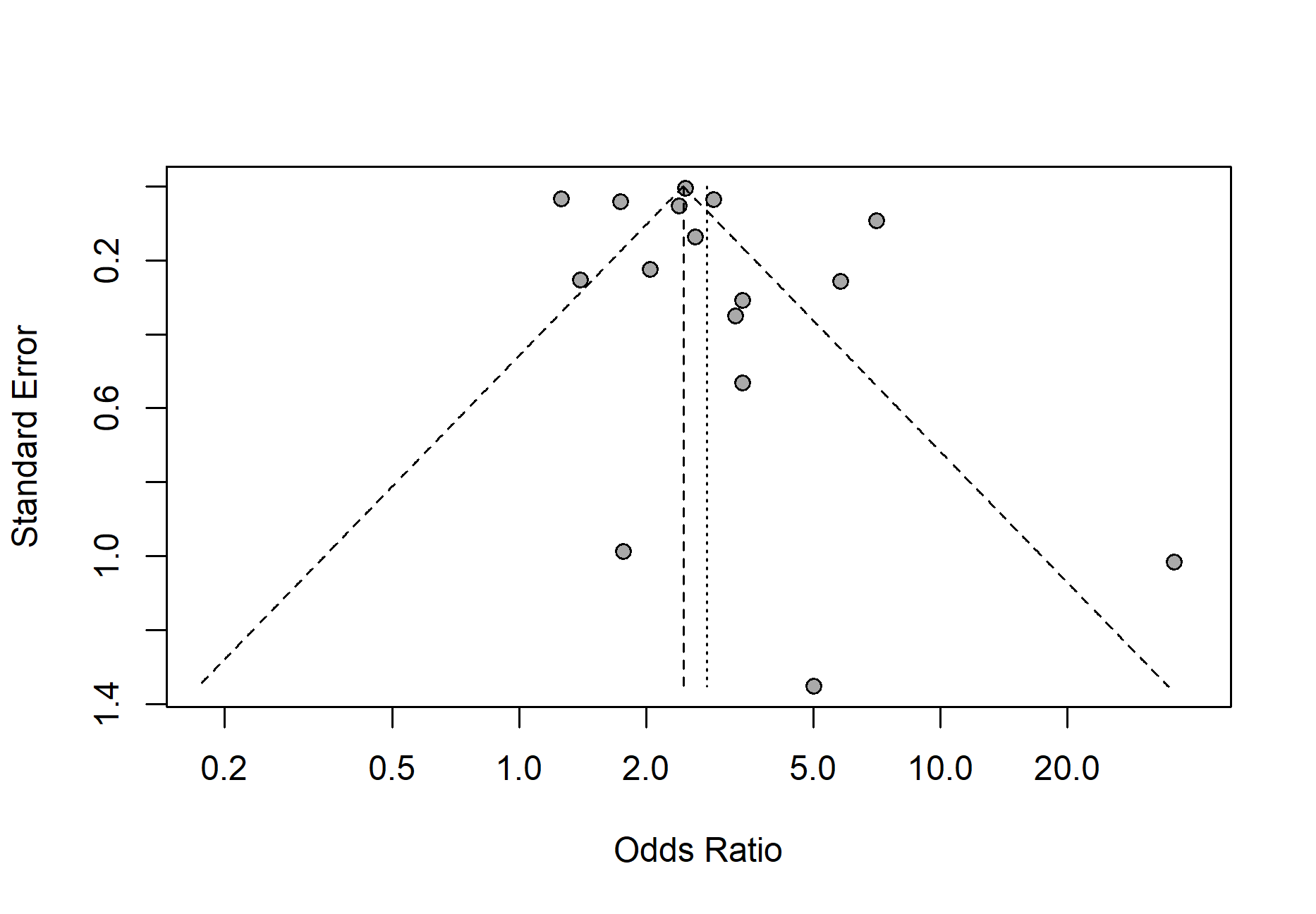


Table 9.4 SLE LBW HEW AND BUNDHUN less overlap as He w searched for studies after 2016,where as Bundhun 2001-2016


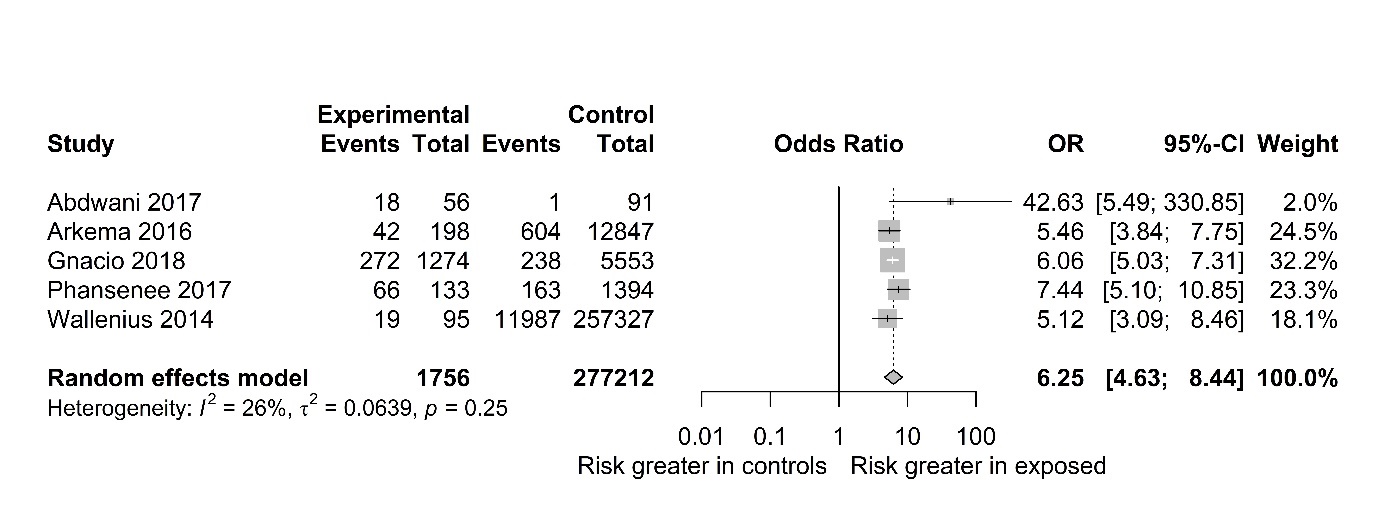


**Table 9.4 Forest plot Thyroid autoimmunity**

**Table 9.4.1 Meta-analysis of cohort and case control studies looking at association of miscarriage and thyroid autimmunity Thangiratnam**


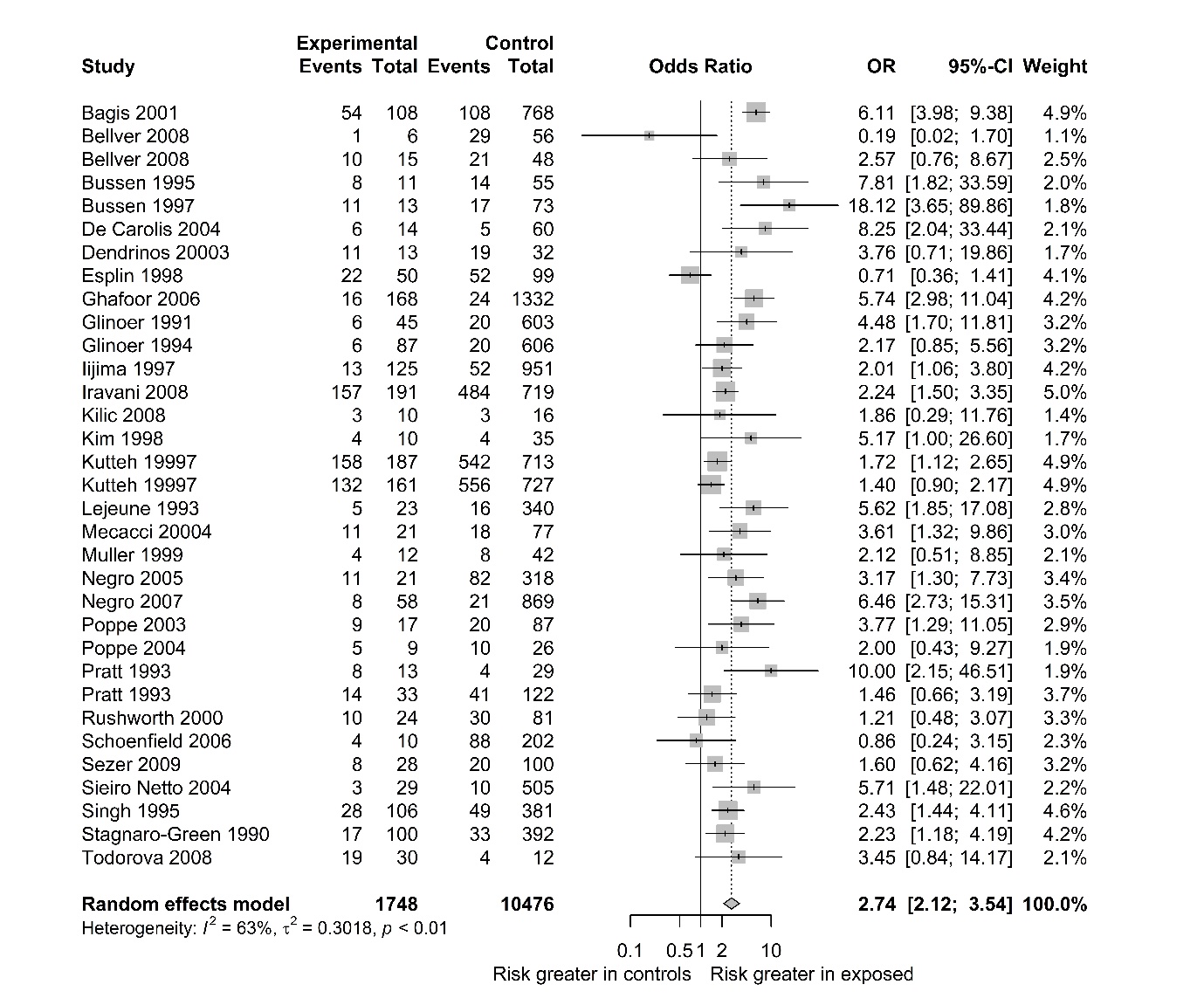


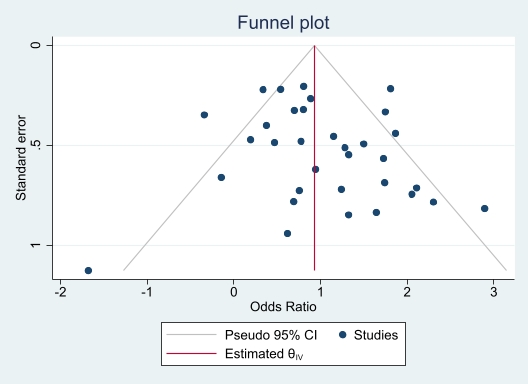


**Table 9.4.2 Meta-analysis of cohort and case control studies Chen 2011 looking at association TAA and Miscarriage**


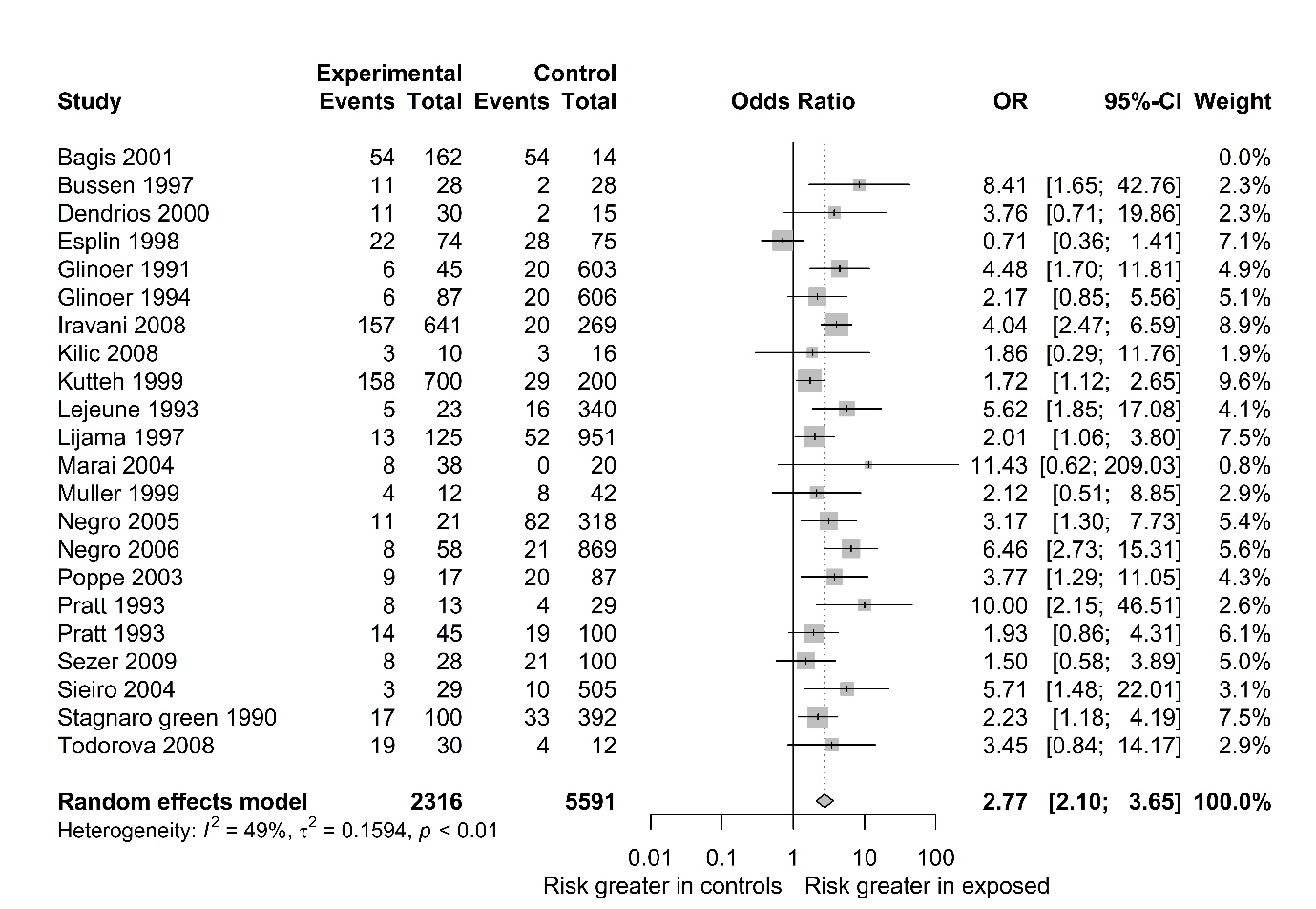


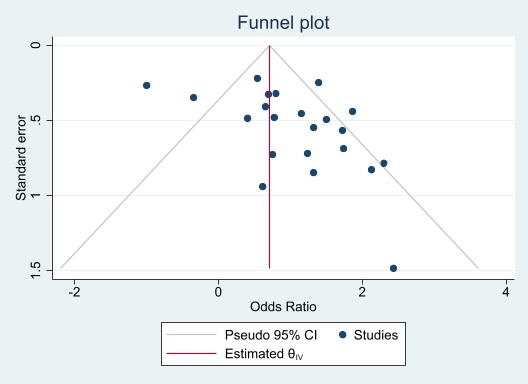


Table 10 Evalution for need to update (48)


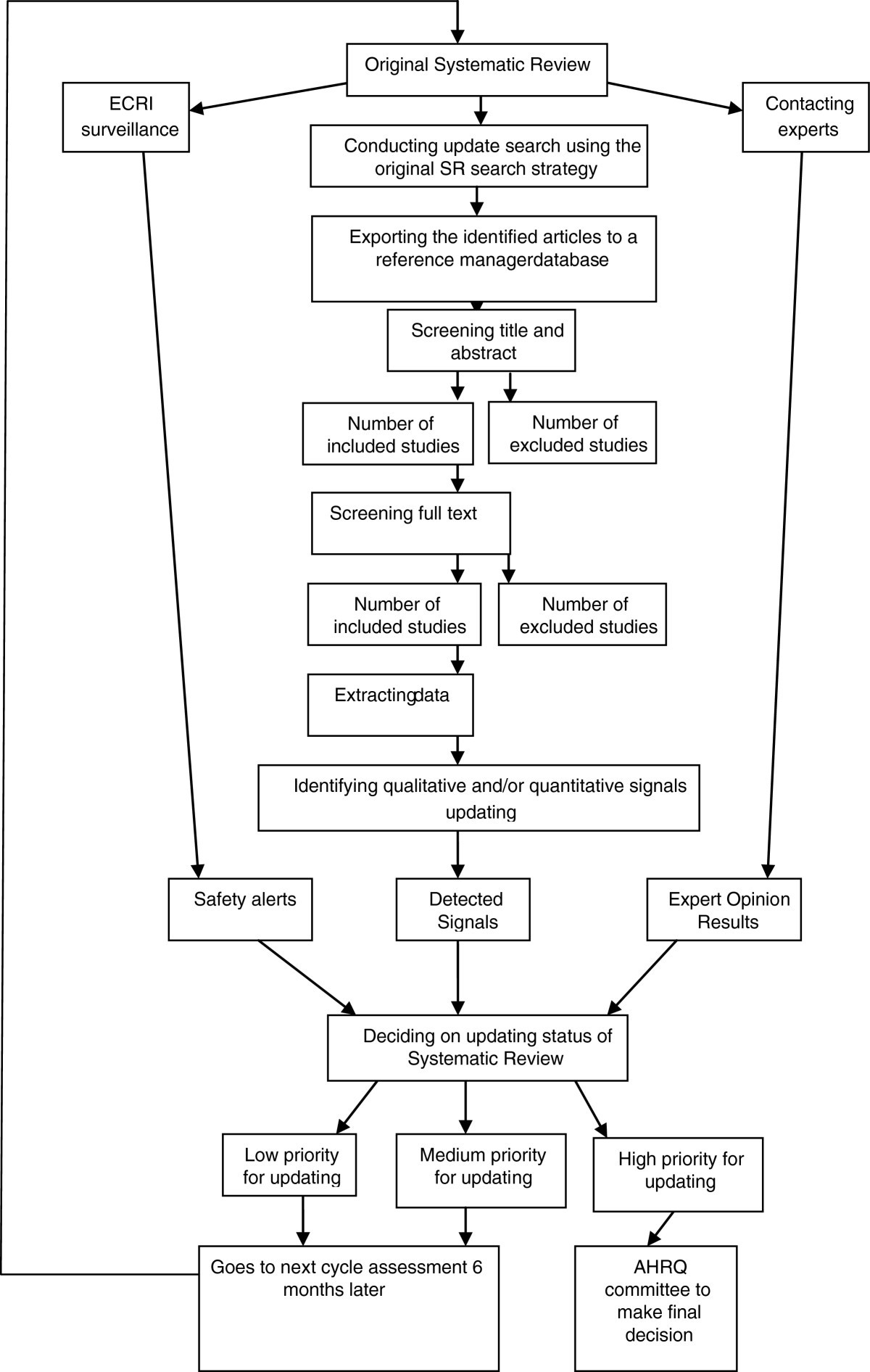


| **Ottawa’s label** | **Ottawa method** |
| --- | --- |
|  | **Qualitative criteria for potentially invalidating signals** |
| A1 | Opposing findings: a pivotal^*^ trial or systematic review (or guidelines) including at least one new trial that characterized the treatment in terms opposite to those used earlier |
| A2 | Substantial harm: a pivotal trial or systematic review (or guidelines) whose results called into question the use of the treatment based on evidence of harm or that did not proscribe use entirely but did potentially affect clinical decision-making |
| A3 | A superior new treatment: a pivotal trial or systematic review (or guidelines) whose results identified another treatment as significantly superior to the one evaluated in the original review, based on efficacy or harm |
|  | **Qualitative criteria for signals of major changes** |
| A4 | Important changes in effectiveness short of ‘opposing findings’ |
| A5 | Clinically important expansion of treatment |
| A6 | Clinically important caveat |
| A7 | Opposing findings from discordant meta-analysis or non-pivotal trial |
|  | **Quantitative criteria signals of changes in evidence** |
| B1 | A change in statistical significance (from nonsignificant to significant) |
| B2 | A change in relative effect size of at least 50 percent |
| **RAND’s label** | **RAND method indications for the need for an update** |
| 1 | Original conclusion is still valid and this portion of the original report does not need updating. This conclusion was reached if we found no new evidence or only confirmatory evidence and all responding experts assessed the CER conclusion as still valid, we classified the CER conclusion as still valid |
| 2 | Original conclusion is possibly out-of-date and this portion of the original report may need updating. This conclusion was reached if we found some new evidence that might change the CER conclusion, and/or a minority of responding experts assessed the CER conclusion as having new evidence that might change the conclusion, then we classified the CER conclusion as possibly out-of-date |
| 3 | Original conclusion is probably out-of-date and this portion of the original report may need updating. This conclusion was reached if we found substantial new evidence that might change the CER conclusion, and/or a majority of responding experts assessed the CER conclusion as having new evidence that might change the conclusion, then we classified the CER conclusion as probably out-of-date |
| 4 | Original conclusion is out-of-date. This conclusion was reached if we found new evidence that rendered the CER conclusion out-of-date or no longer applicable; we classified the CER conclusion as out-of-date. Recognizing that our literature searches were limited, we reserved this category only for situations where a limited search would produce *prima facie* evidence that a conclusion was out-of-date, such as the withdrawal of a drug or surgical device from the market, a black box warning from FDA, and so on |

| **Author**  **Year** | **Geographical area** | **Countries included** | **Database searched** | **search period** | **Studies included year range** | **Number of studies included** | **Study design** | **Exposure** | **Outcome** | **Data synthesis method** | **Quality assessment tool** | **Quality of the primary studies** | **Funding** | **Methods of assessing publication bias#** |
| --- | --- | --- | --- | --- | --- | --- | --- | --- | --- | --- | --- | --- | --- | --- |
| Arafa 2021. | Egypt | US, Finland,  Turkey, England | MEDLINE (PubMed), Cochrane Library, and Google Scholar | inception-Oct.- 2020 | 2002-2019 | 8 | Retrospective and prospective cohort studies | Multiple sclerosis | PE, | meta-analysis | NOS | All studies were reported as moderate to high quality | Not reported | Funnel plot |
| Arvanitakis  2022 | Greece | Slovenia, USA, Italy, Israel,  Denmark,  Sweden,UK, Brazil | Medline, Cochrane  Library and Scopus | inception till  12th April 2022, | 1990-2021 | 18 | Cohort and case control studies | Coeliac disease | Miscarriage, IUGR, PE, stillbirth,CS,, PPH | meta-analysis | NOS | Two studies rated as high quality, six studies as moderate, one study as poor quality. | Not reported | Funnel plot |
| Banner 2021 | Canada | Norway,Portugal,Brazil,Taiwan,Mexico,Turkey,Tunisia,China,France,China,switzerland,US,Yugoslavia,Poland,Canada,Spain,Croatia,canada | Ovid MEDLINE, (2) Ovid EMBASE, (3) Ovid EBM Reviews – Cochrane Central Register of Controlled Trials, (4) PubMed (Non-Medline records), (5) Web of Science and (6) LILACS (Latin American & Caribbean Health Science Literature). | inception-July 2020 | 1968-2020 | 32 | randomized controlled trials, prospective or retrospective cohort studies and case series o | Myasthenia gravis | CS,PTB | Descriptive | Quality Assessment Tool for Case Series | All studies were reported either moderate or low in quality. | The authors received no financial support for the research, authorship and/ or publication of this article | Funnel plot |
| Blagojevic 2020 | USA | Italy, Egypt, USA | PubMed, Cochrane, EMBASE, and Web of Science. | 1950 and February 1, 2018, | 1989-2017 | 16 | randomized clinical trials, retrospective or observational studies, registries, case series with and without controls, and case reports with ≥ 10 pregnancies. | Systemic sclerosis | PE, LBW, PTB, IUGR, Miscarriage | Meta-analysis | NOS | One study reported to be of high quality, four were of medium quality, and three were of low quality | Not reported | Funnel plot and Egger’s test |
| Bundhun 2017. | China | Sweden, Canada, USA,  Taiwan, Denmark, USA, Trinidad, Iran, Norway, Canada, Quebec | Cochrane Database of Randomized Controlled Trials, EMBASE (www.sciencedirect.com) database, and Medline data base: | 2001-2016 | 2001-2016 | 11 | Case control,Cohort studies | SLE | PE, EP, CS, miscarriage, SGA, LBW, PTB | Meta-analysis | Not reported | No information | This research was supported by National Natural Science Foundation of China (No. 81560046), Scientific Project of Guangxi  Higher Education (No. KY2015ZD028) and the Youth Science  Foundation of Guangxi Medical University (No. GXMUYSF201308) | Funnel plot |
| Chen 2011. | China | Brazil ,Belgium, USA, Japan, Netherlands, Italy ,Bulgaria, Turkey | Medline, PubMed, EMBASE and the Cochrane library | inception-2010 | 2005-2010 | 22 | Case control studies and cohort studies | TAA(a | Miscarriage | Meta-analysis | Not reported | No information | Not reported | Funnel plot and Egger’s test |
| Cornish 2007 | UK | NA | Medline | 1980 and 2006 | 1986-2005 | 13 | case–control studies and cohort studies | IBD | PTB,SGA,SB,CS, | MA | Quality of  Reporting of Meta-analyses (QUORUM) guidelines. | Out of 12 studies six were reported to be high quality | Not reported | Funnel plot |
| Dong  2020 | USA | NA | The PubMed, EMBASE, Web of Science, and CENTRAL databases | inception-2018 | 1996-2008 | 17 | case control and cohort study,observational,case series | TAA(all) | Recurrent miscarriage | MA | NOS | No information | Not reported | Not assessed |
| Dong  2020 | China | America, China ,Oman, Thailand, Sweden, Egypt, Danish, Norway, Canada | Web of Science, Cochrane Library, and PubMed | inception to June 30, 2018 | 2011-2018 | 10 | Cohort and case control | SLE | PE | MA | NOS | All studies were reported high in quality | Not reported | Not assessed |
| Geng 2022 | China | NA | CNKI, Wanfang Database, Cqvip Database,  PubMed, Web of Science, Embase and Cochrane Library | Inception-March,2022 | 2005 to March 2022 | 9 | Cohort studies | Sjogren € ′s syndrome | Miscarriage PTB, LBW | MA | NOS | Out of nine studies four reported to be high quality | No funding was received. | Funnel plot, Egger’s test and the Begg-Mazumdar test |
| He W  2020 | China | NA | MEDLINE (PubMed), Cochrane Central, Web of Science, EMBASE, and Google Scholar | 2017-2019 | 2017-2019 | 6 |  | SLE | PE, SB, Fetal loss, Miscarriage,  ,CS  ,IUGR,  GDM,PTB,SGA,  LBW | MA | Grade | All studies reported to be moderate quality | None | Not assessed |
| HeX  2012 | China | USA,Japan,Belgium,Finland,China,Europe,India | PubMed, Embase, and Wangfang | inception -2012 | 2000-2012 | 11 | prospective cohort studies | TAA(all) | Preterm birth | MA | NOS | All studies reported to be high quality | None | Funnel plot and Egger’s test  P=0.40 |
| Huang  2022 | China | seden,Denmark,Canada,USA,switzerland | Medline (PubMed), EMBASE, and Web of Science | Inception -2021 | 2003-2021 | 18 | observational studies including cohort, case‒control, and cross-sectional studies | Rheumatoid arthritis | PE, GHT ,GDM, ,PTB,LBW,SGA,stillbirth/neonata/perinatal death, miscarriage | MA | NOS | Four studies were considered to be of moderate quality, and the rest were considered to be of high quality. | This study was funded by the Medical Health Science  and Technology Project of Zhejiang Provincial Health Commission  (2020KY429) | Not assessed |
| Korevaar 2020 | Netherlands | Netherland, Chile, Australia | Ovid MEDLINE, EMBASE, Web of Science, Cochrane Central Register of Controlled Trials, and Google Scholar databases | inception2018 | 1990-2018 | 19 | Cohort studies | TPO | PTB | Meta-analysis | NA | All studies reported to be high quality | This work was supported by  replication studies grant 401.16.020 from the  Netherlands Organization for Scientific Research. | Not assessed |
| Leung  2021 | Canada | UK, Spain ,Europe, Netherlands, Norway, Korea, Italy France, Israel, India, Finland, Portugal,  Australia  ,Canada | g Medline, Embase, and the Cochrane Central Register  of Controlled Trials | inception-2019 | 1990-2018 | 72 | Cohort and case control | IBD | PTB,LBW,SGA | Meta-analysis | NOS | Out of 62 studies, 36 studies reported to be moderate quality, and 23 studies  were high quality studies. | Not reported | Egger’s and Begg’s test |
| Li, M  2016 . | China | Asia, Europe | MEDLINE, Excerpta Medica, Wan Fang, China Biological Medicine disc, and China National Knowledge Infrastructure | inception-2016 | 1997-2016 | 18 | retrospective and prospective cohort studies | TAA(all) | PTB | MA | NOS | All studies were rated high quality studies | National Natural Science Foundation of China | Not assessed |
| Lou  2020 | China | Chile,China,Kuwait,Turkey,Finland ,USA,Japan,India,Pakistan,Ireland,Greece | PubMed, Embase, MEDLINE, and Cochrane | inception -2020 | 2008-2020 | 44 | Cohort and case control studies | TAA(all) | GDM | MA | Revman | No information | Natural Science Foundation of China | Begg’s test Egger’s test |
| Maguire 2020 | Ireland | Hungary,Norway,croatia,brazil,canada,china,sweden,turkry,iceland,korea,usa,denmark,switzerland | EMBASE, Medline (OVID), CINAHL, Cochrane library (central), Web of Science, and Maternity and Infant Care (MID IRS online) | inception-2019 | 1980-2019 | 18 | Case-control trials, observational studies, cross sectional studies and case series (with n >5) were considered for inclusion | AxSPa | CS,PE,PTB,GDM,IUGR,SB,LBW,SGA | MA | NOS | Ten studies reported to be high quality, Four moderate quality and four poor quality. | e Irish Society of Rheumatology on behalf of UCB as part of the Rheumatology Patient Improvement Fund for Ireland | Funnel plot |
| Milandi 2020 | Netherlands | Spain,UK,Netherlands, | MEDLINE, EMBASE, Web of Science, Cochrane Library, and CINAHL | March to October 2018 i | 1993-2019 | 5 | cohort studies | TPO | PPD | MA | NOS | All the included studies were considered as high quality. | This work was supported by Ministero dell’Università e della Ricerca Scientifca, Italy. | Funnel plot, Egger’s test and Begg’s rank correlation test |
| Modrego 2021 | spain | NA | PubMed, Cochrane Library and EMBASE | 1967 to October 2019 | 1981-2019 | 17 | cohort studies | Multiple sclerosis | PTB,LBW | MA | Not mentioned but quality of primary studies were analysed. | No information | None | Funnel plot |
| O'Toole 2015 | USA | NA | PubMed, EMBASE, the Cochrane Database of Systematic Reviews, and ClinicalTrials.gov | n 1980 and 2014 | 1986-2014 | 23 | case control and cohort studies | IBD | PTB,SGA,SB,CS | MA | NOS | All studies reported to be moderate to low study quality | Not reported | Funnel plot |
| Saccone 2016 | Italy | Italy, UK, Swede, Israel, Ireland, Northern Europe. | Medline, PROSPERO, Scopus, ClinicalTrials.gov, EMBASE, Science direct, the Cochrane Library) were searched | inception-2015 | 2000-2014 | 10 | cohort studies | Coeliac disease | PTB,IUGR,SB,LBW,SGA,PE | MA | ROB assessment tool | All studies were reported to be high quality studies | Not reported | Funnel plot |
| Sim  2023 | UK | NA | MEDLINE, EMBASE, Cochrane, and Scopus. | Inception- May 2022 | 2009-2020 | 13 | Cohort and case control studies | Rheumatoid arthritis | Stillbirth, PTB | MA | NA | Six studies were reported to be high quality and three were moderate quality | Not reported | Funnel plot |
| Talavera 2021 | USA | UK, Denmark, USA | MEDLINE, Web of Science, and CINAHL databases | inception-2020 | 2012-2019 | 5 | cohort, case–control, and cross-sectional studies | IBD | EP | MA | GRADE (Grading of Recommendations, AssessmentDevelopment and Evaluations) | All studies are categorized as moderate or low | None | Egger’s test |
| Tandon 2020 | Canada | NA | Medline, Embase and Cochrane Central Register of Controlled Trials | inception-2015 | 2007-2017 | 53 | Case control,case series ,cohort studies | IBD | CS,GDM,PE, miscarriage ,termination of preg | MA | NOS | 12 studies moderate quality and and 34  studies were high quality | None | Begg’s and Egger’s test |
| Tersigni 2014 | Italy | NA | Medline and Embase Current Contents databases | inception-2012 | 1996-2013 | 24 | Case control,case series,cohort studies | Coeliac disease | miscarriage, recurrent miscarriage, stillbirth, IUGR, LBW, SGA) | MA | NOS | No information | None | Begg’s test Egger’s test |
| Thangaratinam 2011 | UK | NA | s Medline, Embase, Cochrane Library, and SCISEARCH | inception-2011 | 1991-2011 | 36 | Case control,case series,cohort studies | TPO | Misscarriage ,PTB | MA | NOS | Nine studies reported high quality and ten reported moderate quality | None | Not assessed |
| Tong  2016 | China | NA | g PubMed, Embase, and Cochrane database | inceptin-2015 | 2009-2015 | 7 | Case control,case series,cohort studies | TPO | IUGR, , LBW, miscarriage | MA | NOS | All 13 studies had high quality | Not reported | funnel plots and Egger’s test |
| Upala  2015 | USA | NA | MEDLINE and EMBASE | inception-2016 | 2000-2015 | 7 | Case control,case series,cohort studies | Sjögren’s syndrome | PTB, SB, Miscarriage  ,LBW,  SGA, IUGR, | MA | NOS | One high quality study, 3 low quality studies and rest were evaluated as moderate quality | None | Funnel plot |
| Wei  2017 | China | Saudi arabia,USA,china,Spain,China,Greece,Egypt,Korea,Turkey,UK,Janpan,Mexico | PubMed, Embase, Medline and Cochrane Library | inception-2016 | 1990-2016 | 24 | Case control,case series,cohort studies | SLE | PTB | MA | Science Citation Index | All high quality studies | This work was supported by the National Natural Science Foundation of China (Grant Number 81301371), the Guangdong Natural Science Foundation | Not assessed |
| Xie 2021 | China | USA ,Israel, China, Denmark, Sweden ,Canada | PubMed, EMBASE and the Cochrane Library) | inception-2020 | 2007-2020 | 16 | Case control or cohort | Psoriasis and psoriatic arthritis | Psoriasis-CS,PTB,PE or PE,GDM,GTN,Miscarriage, ,APH/PPH,SGA,LBW,Neonatal mortality  ,SB,, PTB CS,PTB,PE, eclampsia, GDM, GTN | MA | NOS | No information | National Natural Science Foundation of China | Begg’s test and Egger’s test |
| Yu  2017 | China | NA | PubMed, ISI Web of Science,  Cochrane Library, Embase and SCOPUS | 1990-2017 | 1990-2017 | 100 | Case control or cohort | databases and 2 from reference lists. | PTB, LBW, SGA ,SB, GHT, PE,CS | type 1 DM |  | No information | Not reported | Funnel plots |
| Zhang  2016 | China | Belgium, Brunei, Spain, Italy, UK, China, India, | PubMed, Cochrane Library, Science Direct, Embase, Chinese Biomedicine and Wanfangdata | inception-2015 | 2003-2015 | 7 | Case control or cohort | TPO | adverse obstetric outcomes: miscarriage, PTB,GHT, placental abruption, IUGR | MA | NOS | No information | Not reported | Funnel plots |

1. *Abbreviation*: *CER* comparative effectiveness review, *FDA* Food and Drug Administration.
2. *a pivotal trial is defined as trial that is published in one of the top five general medical journals or a trial whose sample size is at least triple that of the largest trial in the original systematic review.
3. Legend: Table [1](https://systematicreviewsjournal.biomedcentral.com/articles/10.1186/2046-4053-2-104#Tab1) presents the criteria used to determine if a conclusion is out of date within an SR (here CER). Criteria A1 to B2 come from the Ottawa method and criteria 1 to 4 are based on the RAND method

Source (flow diagram and tables): Ahmadzai N, Newberry SJ, Maglione MA, Tsertsvadze A, Ansari MT, Hempel S, et al. A surveillance system to assess the need for updating systematic reviews. Syst Rev. 2013;2:104.

Table 11. General characteristics of systematic reviews included in the umbrella review

GHT-gestational hypertension, PE-pre-eclampsia, GDM gestational diabetes mellitus, CS-Caesarean section, IUGR-intrauterine growth restriction, SGA-small for gestational age, PTB-preterm birth, LBW-low birth weight, AxSPa-axial spondyloarthropathy, IBD-inflammatory bowel disease ,SLE-systemic lupus erythematosus, T1DM-type 1diabetes mellitus, NOS-Newcastle Ottawa scale for quality assessment, TPO thyroid peroxidase antibodies, TAA- thyroid antibodies all,

Table 12 Tabular presentation of findings: Meta-analysis

| **Autoimmune condition** | **Author**  **Year** | **Outcome** | **No**  **of studies** | **No. of**  **participants** | **Summary estimates** | **I^2^** |
| --- | --- | --- | --- | --- | --- | --- |
| Multiple sclerosis | Arafa  2021 | Pre-eclampsia | 8 | 10012075 | OR  0.99  (0.89-1.09) | **I^2=^**0% |
| Coeliac disease | Arvanitakis  2022 | Caesarean section | 9 | 17961825 | RR  1.10  (1.03-1.16) | **I^2=^**0% |
|  |  | Miscarriage | 7 | 86219 | RR  1.35  (1.10-1.65) | **I^2=^**62% |
|  |  | Postpartum haemorrhage | 4 | 15030472 | RR  1.11  (0.96-1.27) | **I^2=^**0% |
|  |  | Intrauterine  Growth  restriction | 5 | 17472005 | RR 1.68(1.34-2.10) | **I^2=^**39% |
|  |  | Pre-eclampsia | 4 | 14887501 | RR 1.04(0.88-1.23) | **I^2=^**0% |
|  |  | Stillbirth | 6 | 453365 | RR 1.57  (1.17-2.10) | **I^2=^**51% |
|  |  | Preterm birth | 9 | 19445871 | RR  1.29  (1.12-1.49) | **I^2=^**58% |
| Systemic sclerosis | Blagojevic  2020 | Miscarriage | 6 | 5623 | OR  1.6  (1.29-2.22) | P=0.006 |
|  |  | Intrauterine  growth  restriction | 3 | 11203959 | OR  3.2  (2.21-4.53) | P=0.48 |
|  |  | Preterm birth | 7 | 12195889 | OR  2.4  (1.14-4.86) | P=0 |
|  |  | Pre-eclampsia | 2 | 3959 | OR  2.2  (2.21-4.53) | P=0.10 |
|  |  | Low birth weight | 4 | 4577 | OR  3.8  (2.16-6.56) | P=0.80 |
|  |  | Gestational hypertension |  |  | OR  2.80  (2.28–3.39) | P=0.70 |
|  |  | Caesarean section |  |  | OR  2.30  (1.37-3.80) |  |
| SLE | Bundhun  2017 | Caesarean section | 4 | 482389 | RR  1.85  (1.63-2.10) | I **I^2^**=0% |
|  |  | Pre-eclampsia | 4 | 288306 | RR  1.91  (1.44-2.53) | **I^2^**=44% |
|  |  | Gestational hypertension | 4 | 498259 | RR  1.99  (1.54-2.56) | **I^2^**=38% |
|  |  | Miscarriage | 4 | 2974 | RR  1.51  (1.26-1.82) | **I^2^**=38% |
|  |  | Ectopic pregnancy | 2 | 1461 | RR  1.79  (0.57-5.59) | **I^2^**=0% |
| Thyroid autoimmunity (all) | Chen  2011 | Miscarriage | 14 | 5469 | OR  2.77  ( 2.10-3.65) | **I^2^**=49% |
| Inflammatory  bowel  disease | Cornish 2007 | Stillbirth | 4 | 242174 | OR  1.48  (0.89 - 2.47) | **I^2^**=11.3% |
|  |  | Caesarean section | 6 | 298934 | OR  1.50  (1.26 -1.79) | **I^2^**=6.9% |
|  |  | Preterm birth | 8 | 299821 | OR  1.87  (1.52 -2.31) | **I^2^**=9.4% |
| Thyroid autoimmunity (all) | Dong  2020 | Recurrent pregnancy loss- | 17 | 3600 | OR  1.94  (1.43-2.84) | I^2^=51% |
| SLE | Dong 2020 | Pre-eclampsia | 10 | 9456789 | RR  2.99  (2.31-3.88) | I^2^=76% |
| Sjogren′s syndrome | Geng 2022 | Miscarriage | 5 | - | RR  8.85  (3.10-25.26) | I^2^=53% |
|  |  | Preterm birth | 9 | - | RR  2.27  (1.46-3.52) | I^2^=63% |
|  |  | Low birth weight | 5 | - | RR  1.99  (1.34-2.97) | I^2^=42% |
| Systemis  Lupus  erythmatosus | He W  2020 | Preterm birth | 6 | 8797314 | RR  (2.33  (1.78-3.05) | I^2^=94% |
|  |  | Caesarean section | 4 | 11651 | RR  1.38  (1.11-1.70) | I^2^=95% |
|  |  | Gestational diabetes mellitus | 4 | 9603 | RR  0.97  (0.57-1.60) | I^2^=80% |
|  |  | Pre-eclampsia | 4 | 8789888 | RR  3.38  (3.15-3.62) | I^2^=0% |
|  |  | LBW | 3 | 8501 | RR  4.78  (3.65-6.26) | I^2^=59% |
|  |  | Stillbirth | 2 | 1499 | RR  16.49  (2.95-92.13) | I^2^=0% |
|  |  | IUGR | 2 | 1448 | RR  6.98  (0.33-147.02) | I^2^=79% |
|  |  | Miscarriage | 3 | 8792890 | RR  4.70  (3.02-7.29) | I^2^=66% |
| Thyroid autoimmunity (all) | He X  2012 | Preterm birth | 14 | Both  40609 | RR  1.41  (1.08-1.84) | I^2^=75% |
|  |  |  | 9 | Tgab  19191 | RR  0.88  (0.60–1.29) | I^2^=71% |
|  |  |  | 3 | TPOab  11108 | RR  1.69  (1.9–2.41) | I^2^=0% |
| Rheumatoid arthritis | Huang  2022 | Caesarean section | 11 | NA | OR  1.39  (1.24-1.55) | I^2^=85% |
|  |  | Pre-eclampsia | 7 | NA | OR  1.48  (1.19-1.83) | I^2^=81% |
|  |  | Gestational hypertension | 4 | NA | OR  1.34  (1.07-1.68) | I^2^=53% |
|  |  | Gestational diabetes mellitus | 4 | NA | OR  1.40  (0.94-2.09) | I^2^=82% |
|  |  | Miscarriage | 2 | NA | OR  1.16  (1.04-1.29) | I^2^=68% |
|  |  | Small for gestational age | 13 | NA | OR  1.49  (1.22-1.82) | I^2^=87% |
|  |  | Low birth weight | 5 | NA | OR  1.45  (1.30-1.63) | I^2^=0% |
|  |  | Preterm birth | 13 | NA | OR  1.58  (1.44-1.74) | I^2^=63% |
|  |  | Stillbirth/neonatal/perinatal death | 4 | NA | OR  1.38  (1.09-1.74) | I^2^=0% |
| Rheumatoid arthritis | Jiamin  2023 | Stillbirth | 3 | 872048 | OR  1.99  (1.12-3.54) | I^2^=0% |
|  |  | Preterm birth | 6 | 9919225 | OR  1.83  (1.55-2.17) | I^2^=67% |
| Thyroid autoimmunity (TPO) | Korevar  2020 | Preterm birth | 14 | 42608 | OR,  1.33  (1.15-1.56) | I^2^=0% |
| Inflammatory  Bowel  disease | Leung 2021 | Preterm birth  observational | 9 | 5150 | OR  2.13  (1.35-3.36) | I^2^=66% |
|  |  | Preterm birth registries | 7 | 1706443 | OR  1.57  (1.37-1.79 | I^2^=34% |
|  |  | Small for gestational age  observational | 4 | 854 | OR  2.77  (0.70-10.96) | I^2^=76% |
|  |  | Small for gestational age registries | 6 | 1287651 | OR  1.36  (0.99-1.86) | I^2^=74% |
|  |  | Low birth weight  observational | 5 | 1014 | OR  2.78  (1.16-6.66) | I^2^=60% |
|  |  | Low birth weight-registries | 6 | 1161316 | OR  2.45  (1.43-4.21) | I^2^=91% |
| Thyroid autoimmunity (TPO) | Li M  2016 | Preterm birth | 19 | 42733 | OR  1.88  (1.32-2.67) | I^2^=83% |
| Thyroid autoimmunity (all) | Lou J 2021 | Gestational diabetes mellitus  TPO | 13 | 31842 | OR  1.65  (1.13-2.40) | I^2^=74% |
|  |  | Gestational diabetes mellitus TgAb | 3 | 7284 | OR  1.88  (1.13-3.12) | I^2^=0% |
|  |  | Gestational diabetes mellitus both | 2 | 4445 | OR  0.69  (0.44-1.07) | I^2^=0% |
|  |  | Gestational diabetes mellitus All combined | 18 | 43571 | OR  1.49  (1.07-2.07) | I^2^=75% |
| Axial spondyloarthropathy | Maguire,  2020 | Caesarean section | 12 | 121396272 | OR  1.85 (1.462.30) | I^2^=72% |
|  |  | Preterm birth | 11 | 1215684 | OR  0.84  (0.39-1.81) | I^2^=93% |
|  |  | Pre-eclampsia | 7 | 1210607 | OR  1.74  (0.85-3.54) | I^2^=81% |
|  |  | Gestational diabetes Mellitus | 3 | 895 | OR  0.88  (0.24-3.21) | I^2^=45% |
|  |  | Intra-uterine growth retardation | 2 | 148 | OR  1.05  (0.24-4.49) | I^2^=0% |
|  |  | Low birth weight | 8 | 15842 | OR  1.47  (0.98-2.21) | I^2^=34% |
|  |  | Stillbirth | 5 | 2795 | OR  0.70  (0.26-1.85) | I^2^=0% |
|  |  | Small for gestational age | 5 | 1207386 | OR  1.66  (0.93-2.25) | I^2^=72% |
|  |  | Miscarriage | 1 | 56 | OR  0.75  (0.03-19.77) | I^2^=0% |
| Thyroid autoimmunity (TPO) | Milandi  2020 | Postpartum depression | 5 | 2483 | RR  1.49  (1.1-2.00) | I^2^=47% |
| IBD | O toole 2015 | Stillbirth | 10 | 2964983 | OR  1.57  (1.03-2.38) | I^2^=30 |
|  |  | Preterm birth | 33 | 4638932 | OR  1.85  (1.67-2.05) | I^2^=31% |
|  |  | Small for gestational age | 13 | 4132914 | OR  1.36  (1.16-1.60) | I^2^=56% |
| Coeliac disease | Saccone  2016 | Preterm birth-unadjusted | 5 | 4686461 | OR  1.40  (1.18-1.66) | I^2^=43% |
|  |  | Preterm birth- adjusted | 5 | - | OR  1.35  (1.09-1.66) | I^2^=0% |
|  |  | Intra-uterine growth retardation | 6 | 2903065 | OR  2.48  (1.32-4.67) | I^2^=77% |
|  |  | Small for gestational age | 4 | 6906 | OR  4.52  (1.02-20.08) | I^2^=95% |
|  |  | Low birth weight | 3 | 3177908 | OR  1.63  (1.06-2.51) | I^2^=75% |
|  |  | Stillbirth | 5 | 376032 | OR  4.84  (1.08-21.75 | I^2^=90% |
|  |  | Pre-eclampsia | 5 | 519627 | OR  2.45  (0.90-6.70) | I^2^=88% |
| Coeliac disease or  Inflammatory bowel disease(IBD) | Talvera  2021 | Ectopic pregnancy-IBD | 2 | 9276714 | OR  1.26(1.11-1.44 | I^2^=19% |
|  |  | Ectopic pregnancy Coeliac disease | 2 | 70898 | OR  1.21  (0.85-1.71) | I^2^=48% |
|  |  | Ectopic pregnancy ulcerative colitis | 2 | 9276714 | OR  1.03  (0.87-1.23) | I^2^=0% |
|  |  | Ectopic pregnancy Crohns disease | 2 | 9276714 | OR  1.51  (1.21-1.88) | I^2^=52% |
| Inflammatory bowel disease(IBD) | Tandon  2020 | Caesarean section | 7 | 3174 | OR  1.79  (1.16- 2.77) | I^2^=83% |
|  |  | Pre-eclampsia | 2 | 800 | OR  4.65  (0.76-28.35) | I^2^=0% |
|  |  | Miscarriage | 3 | 1700 | OR  1.63  (0.49-5.43) | I^2^=90% |
|  |  | Gestational diabetes mellitus | 3 | 927 | OR  2.96  (1.47-5.98) | I^2^=0% |
| Coeliac disease | Tersigni  2014 | Intra-uterine growth retardation | 3 | 400 | OR  8.73  (3.23-23.58) | I^2^=0% |
|  |  | Small for gestational age | 2 | 841 | OR  2.36  (0.40-14.13) | I^2^=0% |
|  |  | Recurrent miscarriage | 6 | 1164 | OR  5.82  (2.30-14.74) | I^2^=0% |
|  |  | Adverse pregnancy outcomes | 15 | 8983 | OR  4.97  (2.88-8.57) | I^2^=0% |
| Thyroid autoimmunity (all) | Thangaratinam,2011 | Preterm birth | 5 | 12566 | OR  2.07  (1.17-3.68) | I^2^=78% |
|  |  | Miscarriage-case control studies | 10 | 3604 | OR  1.80  (1.25- 2.60) | I^2^=56% |
|  |  | Miscarriage-cohort studies | 19 | 8522 | OR  3.90  (2.48 .12) | I^2^=81% |
| Thyroid autoimmunity (TPO) | Tong  2016 | Intra-uterine growth restriction | 7 | 15269 | OR  1.57  (0.77-3.18) | I^2^=82% |
| Sjogren syndrome | Upala  2015 | Stillbirth | 3 | NA | OR  1.05  (0.37–2.97) | I^2^=0% |
|  |  | Preterm birth | 5 | NA | OR  2.10  (0.59– 7.46) | I^2^=59 % |
|  |  | Fetal loss | 4 | NA | OR  1.77  (1.28–2.46) | I^2^=0% |
| Systemic lupus erythmatosus | Wei  2017 | Preterm birth | 6 | NA | RR  2.05  (1.27-3.32) | I^2^=66.5% |
| Psoriasis | Xei  2021 | Caesarean section | 5 | NA | OR  1.26  (1.05-1.51) | I^2^=92% |
|  |  | Preterm birth | 6 | NA | OR  1.22  (1.04-1.42) | I^2^=83.1% |
|  |  | Miscarriage | 2 | NA | OR  1.10  (1.01-1.20) | I^2^=0% |
|  |  | Small for gestational age | 4 | NA | OR  1.01  (0.92-1.11) | I^2^=49% |
|  |  | Stillbirth | 3 | NA | OR  1.17  (0.84-1.63) | I^2^=67% |
|  |  | Low birth weight | 4 | NA | OR  1.14  (0.87-1.49) | I^2^=81% |
|  |  | Gestational hypertension | 3 | NA | OR  1.29  (1.15-1.45) | I^2^=32% |
|  |  | Pre-eclampsia | 5 | NA | OR  1.25  (1.09-1.42) | I^2^=66% |
|  |  | Gestational diabetes mellitus | 3 | NA | OR  1.19  (1.09-1.30) | I^2^=0% |
| Type 1 diabetes mellitus | Yu  2017 |  |  |  |  |  |
|  |  | Preterm birth | 15 | NA | OR  4.36  (3.72-5.12) | NA |
|  |  | Small for gestational age 10^th^ percentile | 7 | NA | OR  0.68  (0.56-0.83) | NA |
|  |  | Stillbirth | 12 | NA | OR  3.97  (3.44-4.58) | NA |
|  |  | Gestational hypertension | 7 | NA | OR  2.68  (1.85-3.89) | NA |
|  |  | Pre-eclampsia | 10 | NA | OR  4.19  (3.08-5.71) | NA |
|  |  | Caesarean section | 17 | NA | OR  3.97  (3.31-4.77) | NA |
| Thyroid autoimmunity (TPO) | Zhang  2016 | Miscarriage | 9 | 13301 | OR  2.02  (1.13-3.62) | I^2^=65% |
|  |  | Gestational hypertension | 8 | 20599 | OR  1.29  (1.00-1.45) | I^2^=0% |
|  |  | Small for gestational age | 3 | 9161 | OR  1.61  (0.211.12) | I^2^=89% |
|  |  | placental abruption in pregnancy | 3 | 13394 | OR  0.42  (0.12-1.43) | I^2^=0% |
|  |  | Preterm birth | 9 | 20573 | OR  1.39  (1.11-1.76) | I^2^=0% |
| Psoriatic arthritis | Xei 2021 | Caesarean section | 4 | NA | OR  1.45  (1.27-1.66) | I^2^=0% |
|  |  | Preterm birth | 4 | NA | OR  1.48  (1.24-1.78) | I^2^=0% |
|  |  | Miscarriage | 1 | NA | OR  1.35  (0.79-2.32) | I^2^=0% |
|  |  | Small for gestational age | 3 | NA | OR  0.99  (0.72-1.36) | I^2^=8% |
|  |  | Stillbirth | 3 | NA | OR  1.06  (0.46-2.46) | I^2^=0% |
|  |  | Low birth weight | 2 | NA | OR  1.15  (0.84-1.58) | I^2^=0% |
|  |  | Gestational  hypertension | 2 | NA | OR  1.49  (1.09-2.06) | I^2^=0% |
|  |  | Pre-eclampsia | 3 | NA | OR  1.45  (1.13-1.85) | I^2^=0% |
|  |  | Gestational diabetes mellitus | 5 | NA | OR  1.26  (0.90-1.77) | I^2^=0% |

OR=odds ratio, RR=Risk ratio

Table 13.Tabular presentation of findings: Narrative syntheses

| Autoimmune condition | Authoryear | Outcome | No. of Studies  included | Sample size | Narrative summary | Authors conclusion | Quality of the review  (AMSTAR 2 rating) |
| --- | --- | --- | --- | --- | --- | --- | --- |
| Myasthenia gravis | Banner  2022 | Caesarean section | 31 | 824 | The overall rate of caesarean delivery done for MG was 25/824 (3.0%), with an additional 249/824 (30.2%) women having a caesarean delivery for an obstetric indication | Women with MG are at increased risk of requiring assisted vaginal delivery or caesarean delivery compared to the general population | Low |
|  |  | Preterm birth, less than 34 weeks  Preterm birth, less than 37 weeks | 19  25 |  | PTB less than 34 and 37 weeks’ gestation occurred in 14/327 (4.3%) and 69/579 (11.9%) pregnancies, respectively. | 14/327 (4.3%) and 69/579 (11.9%) pregnancies, respectively |  |
|  |  | Small for gestational age | 12 | 250 | gestational age, 55/ 390 (14.1%) neonates were smaller than the 10th percentile. |  |  |
| Multiple sclerosis | Modrego 2021 | Preterm birth  Or  Low birthweight | 17 | NA | Seventeen studies focused specifically on the effects of MS on pregnancy with peripartum outcomes; 14 were retrospective and three were prospective | In 12 of them, there were no significant differences in preterm deliveries, In two retrospective cohorts, there was an increased number of infections and preterm deliveries an increased number of preterm deliveries and low birth weight, low birth weight. | Low |
